# Chemically Induced Mismatch of Rings and Stations in [3]Rotaxanes

**DOI:** 10.1021/jacs.1c02230

**Published:** 2021-04-29

**Authors:** Massimiliano Curcio, Federico Nicoli, Erica Paltrinieri, Ettore Fois, Gloria Tabacchi, Luigi Cavallo, Serena Silvi, Massimo Baroncini, Alberto Credi

**Affiliations:** ‡ Dipartimento di Chimica Industriale “Toso Montanari”, Università di Bologna, Bologna 40136, Italy; § Center for Light Activated Nanostructures, Istituto ISOF-CNR, Bologna 40129, Italy; ⊥ Dipartimento di Scienza e Alta Tecnologia, Università dell’Insubria, Como 22100, Italy; ∥ Kaust Catalysis Center, Physical Sciences and Engineering Division, King Abdullah University of Science and Technology, Thuwal 23955, Saudi Arabia; # Dipartimento di Chimica “Giacomo Ciamician”, Università di Bologna, Bologna 40126, Italy; □ Dipartimento di Scienze e Tecnologie Agro-alimentari, Università di Bologna, Bologna 40127, Italy

## Abstract

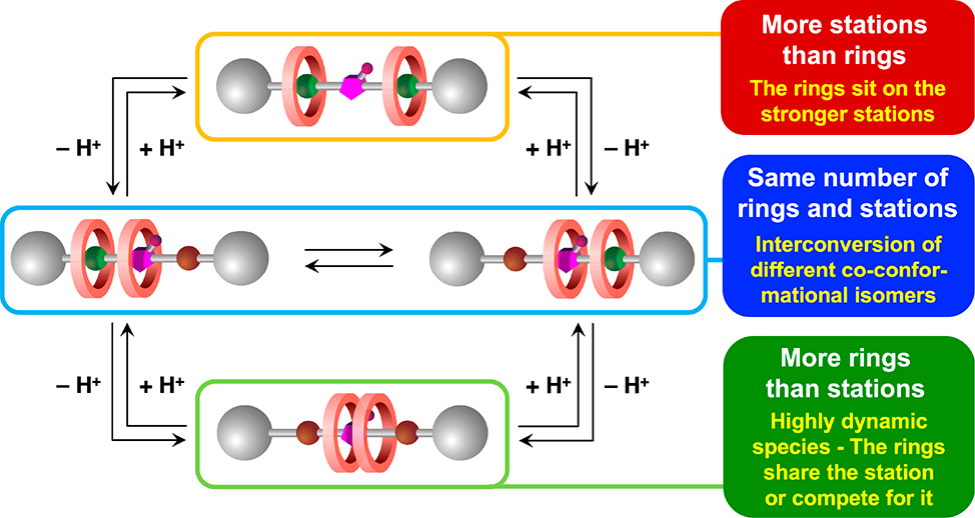

The mechanical interlocking of molecular components can lead to the appearance of novel and unconventional properties and processes, with potential relevance for applications in nanoscience, sensing, catalysis, and materials science. We describe a [3]rotaxane in which the number of recognition sites available on the axle component can be changed by acid–base inputs, encompassing cases in which this number is larger, equal to, or smaller than the number of interlocked macrocycles. These species exhibit very different properties and give rise to a unique network of acid–base reactions that leads to a fine p*K*_a_ tuning of chemically equivalent acidic sites. The rotaxane where only one station is available for two rings exhibits a rich coconformational dynamics, unveiled by an integrated experimental and computational approach. In this compound, the two crown ethers compete for the sole recognition site, but can also come together to share it, driven by the need to minimize free energy without evident inter-ring interactions.

## Introduction

Mechanically interlocked molecules (MIMs) such as rotaxanes have gained considerable attention over the past three decades because of their intriguing structural and dynamic properties^1,2^ and, in more recent years, for their application in key areas such as dyes,^3^ ion recognition^4^ and transport,^5^ catalysis,^6,7^ molecular electronics,^8,9^ structural and functional materials,^10−14^ molecular machines,^15−17^ and nanomedicine.^18^

Most often, the functions brought about by rotaxanes take advantage of a precise relative arrangement of the ring and axle components, which can be engineered through chemical design and potentially controlled by external stimuli. These goals are typically achieved by introducing recognition sites (*stations*) on the axle component in order to establish noncovalent interactions with the macrocycle(s). The presence of stations can be a requirement to template the formation of the MIM,^1,2,19^ although strategies that do not require permanent recognition motifs on the components to be interlocked have been developed.^20^

**Figure 1:**
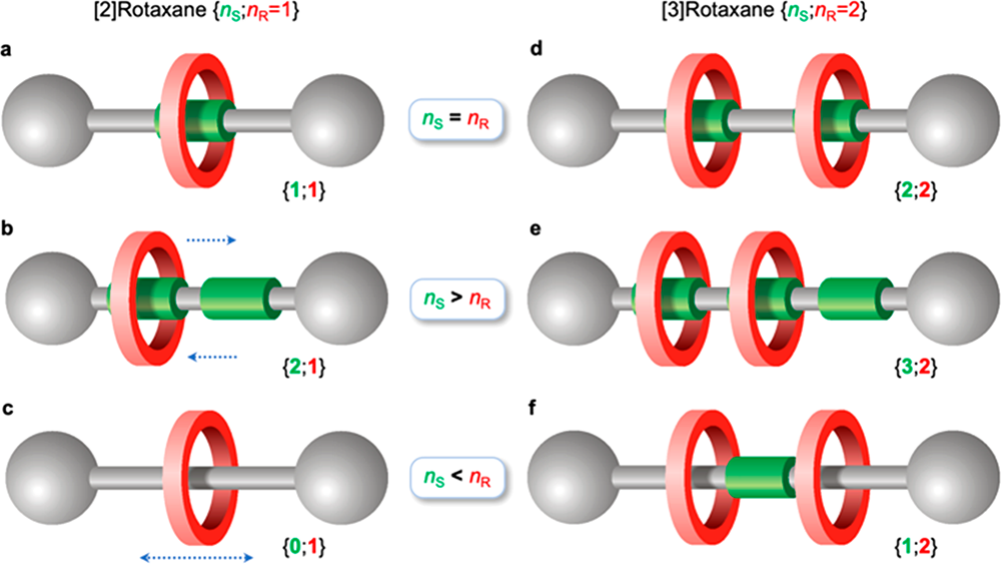
Cartoon representation of [2]rotaxanes (left) and [3]rotaxanes (right) in which the number of recognition sites, or stations (*n*_S_), is respectively (a, d) equal to, (b, e) higher than, and (c, f) lower than the number of macrocyclic rings (*n*_R_). The blue arrows in b and c highlight the kind of translational movements that are typically observed in these rotaxanes.

The balance between the number of stations (*n*_S_) and of rings (*n*_R_) in a rotaxane is a primary design element that leads to very different properties, as shown schematically in Figure 1a–c for a [2]rotaxane.

A common case is when *n*_S_ = *n*_R_ (Figure 1a), that is, the ring sits on the sole recognition site present on the axle; in a rotaxane with more than one ring, it means that there is one station available to each ring. Also popular is the instance when *n*_S_ > *n*_R_ (Figure 1b); this is the case of molecular shuttles, where the translational movement of the ring between the stations can occur either randomly or under the control of physical or chemical stimuli.^1,2,4−6,8,9,14,16,18^

Much less investigated is the case in which *n*_S_ < *n*_R_, i*.*e., there are not enough stations for the rings; in a [2]rotaxane, this means that the axle contains no recognition sites (i*.*e*.*, *n*_S_ = 0; Figure 1c). Such a scenario is particularly intriguing because the relative arrangement of the components is not dominated by strong intermolecular interactions, and peculiar structural and/or dynamic effects purely arising from the mechanical bond could emerge.^21−25^

Such a condition is even more interesting for [3]rotaxanes that comprise two rings encircling the same axle, as it can be fulfilled with *n*_S_ ≠ 0; in other words, a MIM could be obtained in which there is only one station available to two rings (Figure 1f). This “frustrated” state could lead to novel coconformational phenomena that range from exclusive competition of the rings for the station in a highly dynamic fashion to an arrangement in which the two rings come together to both interact with the station^26^ without necessarily interacting with each other as observed in previously studied [3]rotaxanes.^27−29^ Obviously, [3]rotaxanes characterized by *n*_S_ > *n*_R_ (Figure 1e) and *n*_S_ = *n*_R_ (Figure 1d) can also be obtained.

**Scheme 1:**
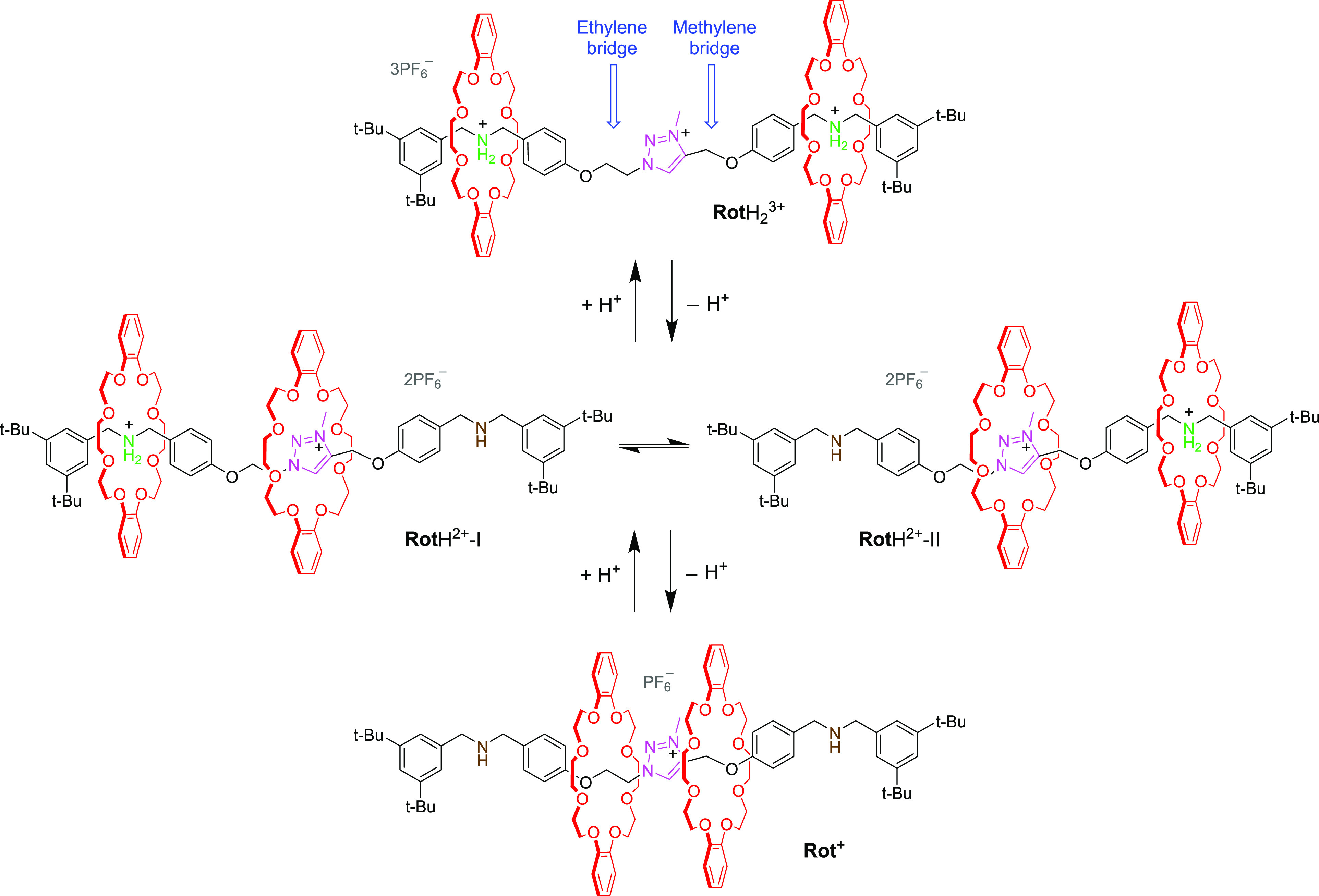
Structural Formulas and Acid–Base Triggered Interconversion of the [3]rotaxanes Studied in This Work

The efficient synthetic routes available nowadays in rotaxane chemistry allow a rational approach to this issue by designing modular [3]rotaxanes where the proportion between rings and stations can not only be predetermined, but also varied on command. We thus prepared [3]rotaxane **Rot**H_2_^3+^ (Scheme 1), which consists of two identical dibenzo-24-crown-8 (DB24C8) rings interlocked with an axle containing two lateral dibenzylammonium (Am) and one central triazolium (Tz) stations. On the basis of published data,^4,30,31^ it is expected that each DB24C8 ring encircles an Am station on account of strong hydrogen bonding. The Am stations, however, can be deactivated by deprotonation,^4,30^ affording rotaxanes **Rot**H^2+^ and **Rot**^+^. The former, because of the nonsymmetric structure of the axle, can exist in two nonequivalent isomers that differ for the position of the sole ammonium site and its surrounding ring on the axle. Therefore, we envisioned that acid–base reactions performed on **Rot**H_2_^3+^ could enable the reversible interconversion between the structures shown in Figure 1def, as shown in Scheme 1.

A number of interesting issues can arise from the investigation of such a switchable [3]rotaxane, namely: (i) Considering that the apparent p*K*_a_ of the Am site depends on whether it is encircled by the ring,^32−34^ how does the rotaxane behave upon treatment with a base? (ii) What are the properties of the isomers of **Rot**H^2+^, and how are they interconverted? (iii) What are the structural and dynamic properties of the fully deprotonated rotaxane in which only the Tz station is available to the two DB24C8 rings?

Here we try to answer these questions by a combination of NMR spectroscopy, UV–visible spectroscopy and computational techniques applied to the [3]rotaxanes and appropriate model compounds. Our prime aim is to shine light on unconventional (and for some aspects unique) chemical reactions that can occur in rotaxanes, which may extend the range of interest and application of these MIMs. Another objective is to gather information on switchable [3]rotaxanes of higher complexity than [2]rotaxane-based molecular shuttles, which despite their huge potential^35,36^ are yet scarcely investigated.^37^ A long-term goal of this work is the development of more complex mechanical nanodevices such as a multistate molecular abacus.

## Results and Discussion

**Scheme 2:**
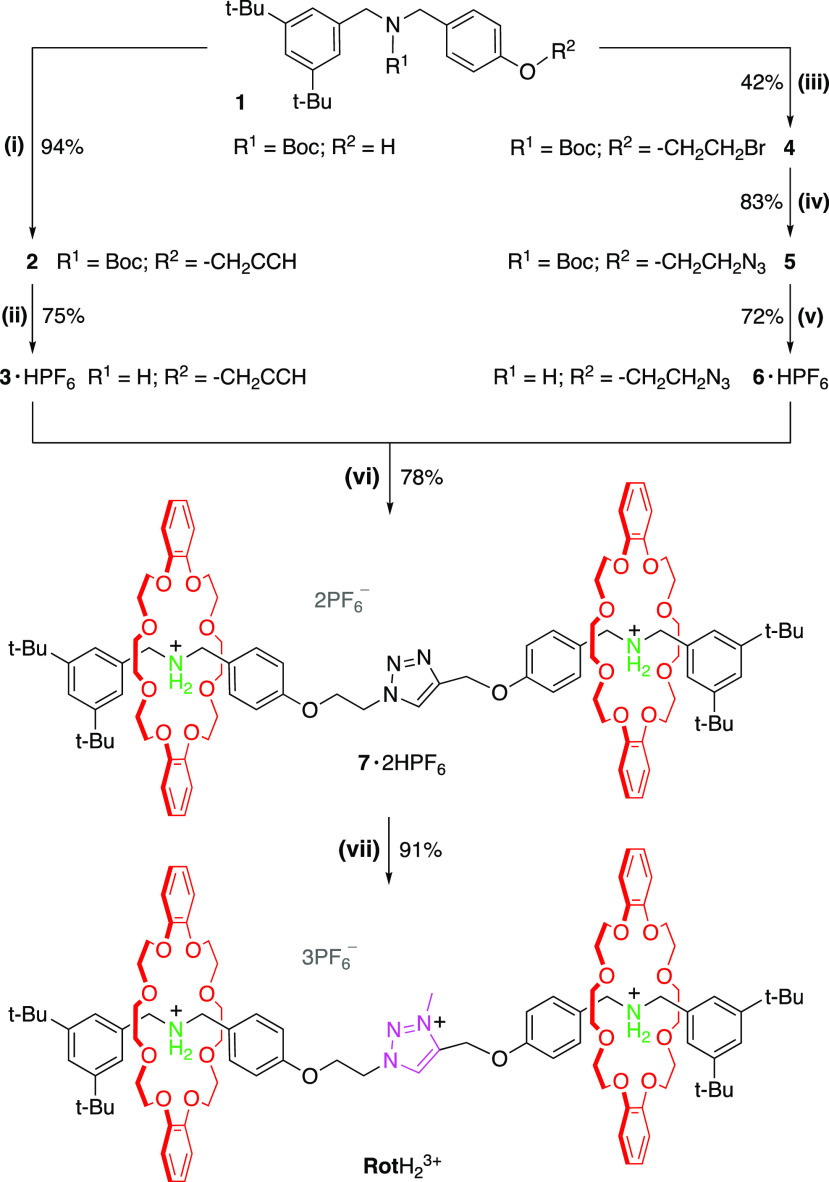
Synthetic Route to the [3]rotaxane **Rot**H_2_^3+^: (i) BrCH_2_CCH, K_2_CO_3_, MeCN; (ii) HPF_6_, THF; (iii) BrC_2_H_4_Br, K_2_CO_3_, MeCN; (iv) NaN_3_, DMF; (v) HPF_6_, THF; (vi) DB24C8, [(MeCN)_4_Cu][PF_6_], CH_2_Cl_2_; (vii) MeI, KPF_6_

The target compound of this study is the [3]rotaxane **Rot**H_2_^3+^, provided with two switchable ammonium stations and one permanent triazolium station. The symmetric structure of the rotaxane enables a straightforward convergent synthetic approach starting from the readily available Boc-protected species **1** (Scheme 2), which is converted through a sequence of functional group exchange reactions into the building blocks of the axle. The rotaxane formation relies on the copper(I)-catalyzed azide–alkyne cycloaddition reaction between **6** and **3** in the presence of the crown ether dibenzo-24-crown-8, affording the dicationic [3]rotaxane **7·**2HPF_6_ in good yield. Reaction of **7·**2HPF_6_ with iodomethane followed by anion exchange provides the desired tricationic rotaxane.

**Figure 2:**
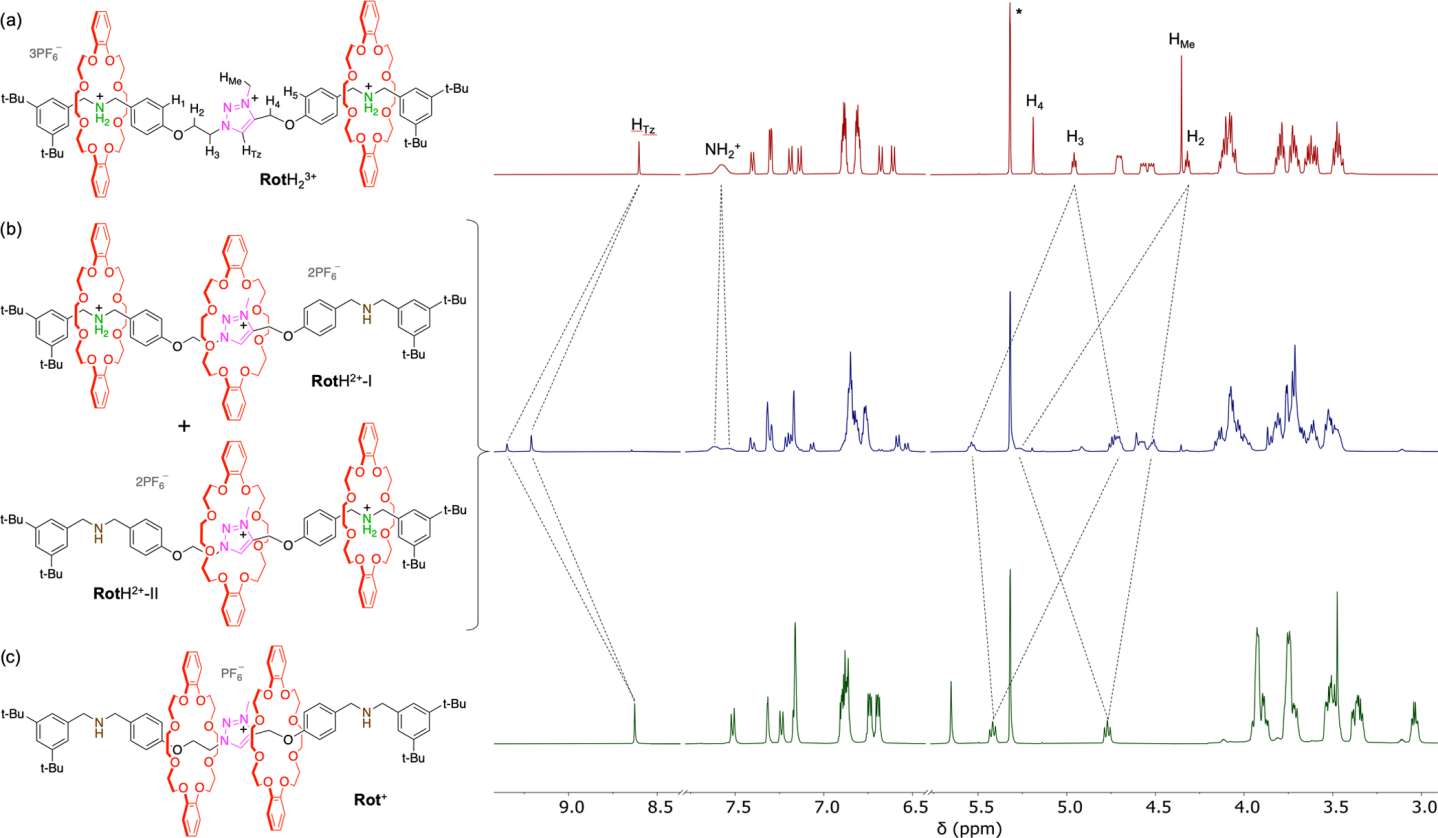
^1^H NMR spectra (500 MHz, CD_2_Cl_2_, 298 K) of (a) trication **Rot**H_2_^3+^ (red trace) and the species observed upon deprotonation with the heterogeneous phosphazene base **B1**: (b) mixture of dications **Rot**H^2+^-I and **Rot**H^2+^-II (blue trace), and (c) monocation **Rot**^+^ (green trace). The peak marked with an asterisk is due to the residual solvent.

**Rot**H_2_^3+^ was characterized by mass spectrometry and NMR experiments, allowing for the assignment of all ^1^H and ^13^C resonances. The most relevant signals in the ^1^H NMR spectrum of **Rot**H_2_^3+^ (Figure 2a) are (i) the sharp singlet at 8.6 ppm, related to the Tz aromatic C–H, and (ii) the peaks in the region between 4.5 and 4.8 ppm, assigned to the benzylic protons exhibiting the characteristic multiplicity of Am stations complexed by crown ether rings.^38^

### Base-Induced Switching

The tricationic rotaxane **Rot**H_2_^3+^ presents two ammonium stations that can be deactivated by the addition of a suitable base. The behavior of **Rot**H_2_^3+^ toward deprotonation was investigated by ^1^H NMR spectroscopy throughout the sequential addition of the heterogeneous phosphazene base **B1** (polystyrene-supported BEMP, see the Supporting Information). As the reaction proceeds, two new products are formed as unambiguously indicated by the two independent sets of signals, each containing a triazolium-related peak (H_Tz_, Figure 2 and Figure S37). The considerable downfield shift displayed by these peaks, moving from 8.60 ppm in **Rot**H_2_^3+^ to 9.35 and 9.21 ppm, is attributed to the complexation of the Tz cation by a crown ether ring upon deactivation of one of the Am stations.^39^ The two new patterns persist until the full conversion of the starting rotaxane **Rot**H_2_^3+^ (addition of one equivalent of **B1**, Figure 2b) and are as such assigned to the two isomers **Rot**H^2+^-I and **Rot**H^2+^-II, formed upon monodeprotonation of the parent compound **Rot**H_2_^3+^. The stoichiometric deprotonation of the rotaxane performed with the heterogeneous base **B1** was validated with a control experiment carried out in homogeneous solution by adding 1 equiv. of the phosphazene base P_1_-*tert*-butyl (**B2**, see the Supporting Information) under the same reaction conditions (Figure S38). The existence of the two species **Rot**H^2+^-I and **Rot**H^2+^-II derives from the quasi-symmetry of the axle, which imparts a nonequivalent nature to the two ammonium stations located on each side of the central triazolium cation in **Rot**H_2_^3+^. The identity of the dicationic rotaxanes was assigned in accordance with their DFT-computed relative stabilities (vide infra).

It is important to note that the **Rot**H^2+^-I/**Rot**H^2+^-II ratio is 30:70; it does not change over time, and it remains the same throughout the whole stepwise deprotonation up to the addition of 1 equiv. of base. Such observations suggest that the two species are in thermodynamic equilibrium and possess different stabilities. On the other hand, forms I and II of **Rot**H^2+^ exhibit distinct signals in the ^1^H NMR spectra, indicating that the exchange between the two species is slow on the NMR time scale. Indeed, EXSY experiments performed on monodeprotonated **Rot**H_2_^3+^ (Figure S40) confirm the presence of an active exchange pathway between **Rot**H^2+^-I and **Rot**H^2+^-II, which takes place with a rate constant of 2.2 s^–1^.

**Figure 3:**
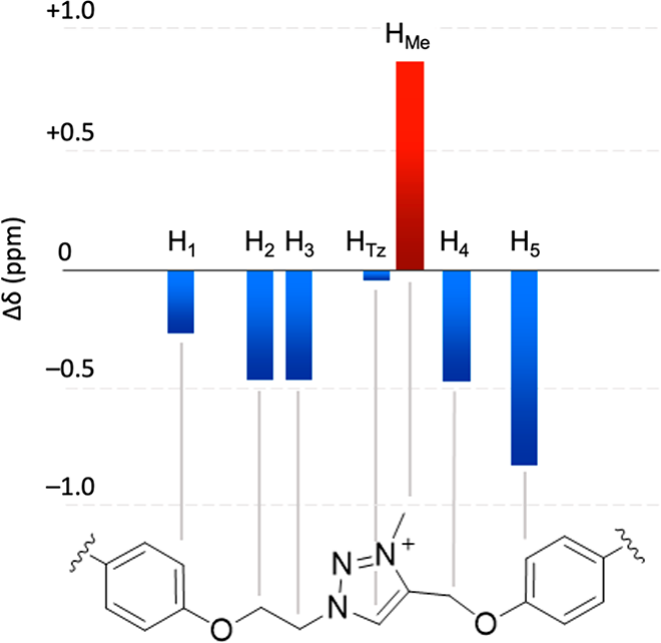
^1^H NMR chemical shift variations upon deprotonation of **Rot**H_2_^3+^ into **Rot**^+^ (500 MHz, CD_2_Cl_2_, 298 K). The Δδ values in red and blue correspond to shielding and deshielding effects, respectively. For clarity, the two DB24C8 rings and the anion have been omitted.

Starting from the mixture of **Rot**H^2+^-I and **Rot**H^2+^-II, further deprotonation with a second equivalent of **B1** produces the monocation **Rot**^+^ as the only product, confirming the correct assignment of the two dicationic intermediates through their convergent reactivity (Figure 2c). The complete deprotonation of **Rot**H_2_^3+^ into **Rot**^+^ causes significant shifts in the related ^1^H NMR spectra (Figure 3). In particular, a deshielding is observed for all the signals related to the nuclei placed around the Tz core, whereas the Tz aryl peak remains almost unaltered and the Tz methyl group becomes shielded by 0.88 ppm.^31,40,41^ These changes suggest that in **Rot**^+^ both rings have moved toward the central triazolium station, with neither of them overtaking it.

The nucleophilic amine sites generated upon deprotonation are in principle available for functionalization. Thus, compound **Rot**^+^ was reacted with di-*tert*-butyl dicarbonate, accessing the bis-carbamate **Rot**Boc_2_^+^ (Figure S41). The ^1^H NMR spectrum of **Rot**Boc_2_^+^ shows a set of peaks that is almost unchanged compared to the one observed in **Rot**^+^, except for the peaks directly influenced by the newly formed carbamate group. The close similarity of the NMR spectra of the deprotonated and Boc-protected rotaxanes confirms that in **Rot**^+^ the rings have moved away from the amine sites. The effortless attainment of **Rot**Boc_2_^+^ is also an indirect evidence for the translation of the crown ether rings toward the center of the rotaxane in **Rot**^+^, as it is well-known that reactivity of functional groups encircled by a macrocycle in a rotaxane can be hindered.^21,32,42,43^

### Thermodynamic Analysis of Deprotonation

Hydrogen bonding interactions established between a crown ether ring and an ammonium station have a considerable impact on the thermodynamic properties of the latter. Upon complexation, the acidity of the ammonium cation dramatically decreases,^32−34^ reaching p*K*_a_ values commonly associated with much weaker acids like guanidinium and phosphazenium cations.^44^ The different ring dispositions observed in **Rot**H_2_^3+^, **Rot**H^2+^, and **Rot**^+^ are thus expected to confer distinct thermodynamic features to the four kinds of ammonium stations—on the side of the ethylene or methylene bridge, free or complexed—comprised in these rotaxanes. We exploited NMR and UV–vis spectroscopies to investigate this peculiar situation in acetonitrile.

**Figure 4:**
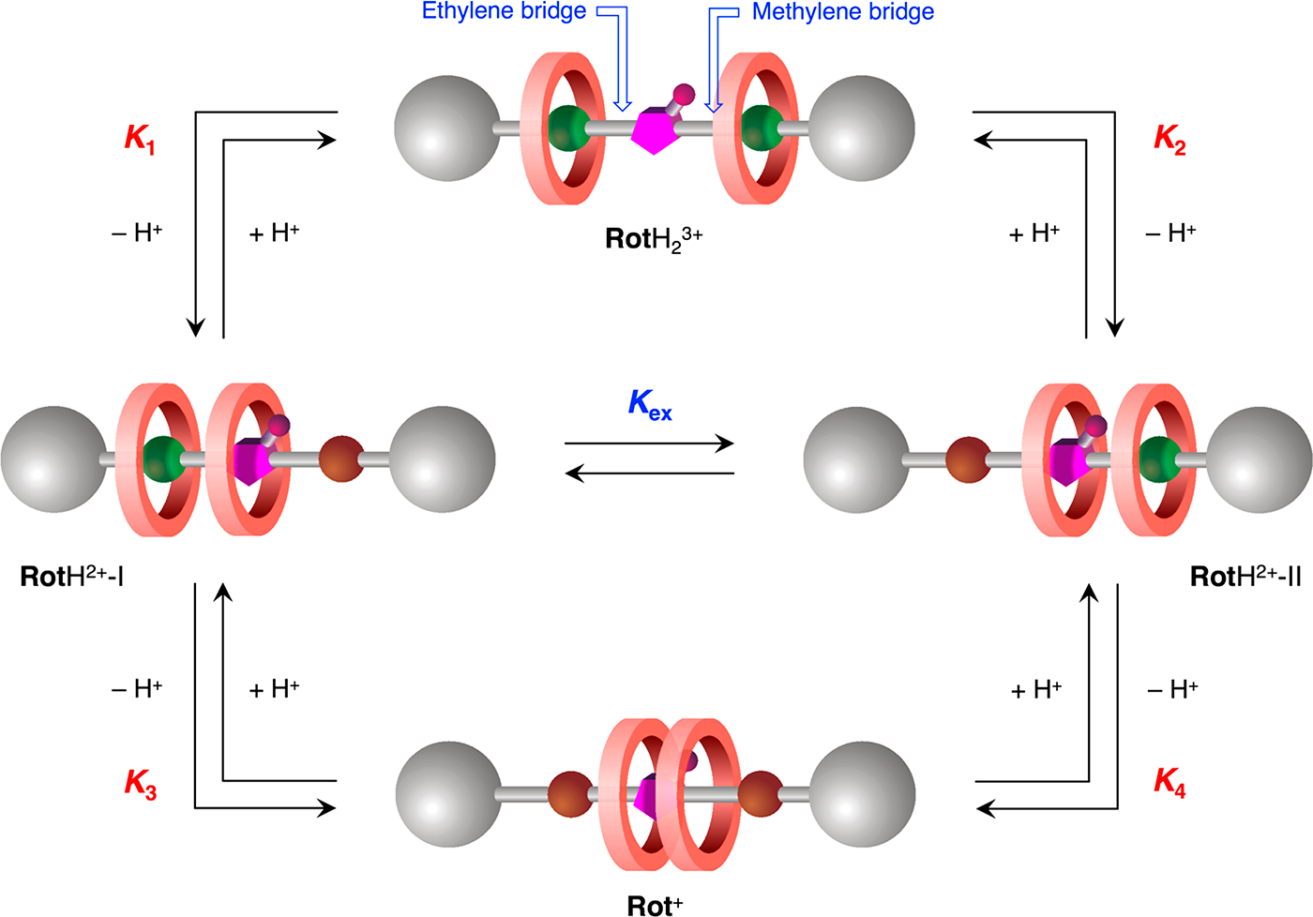
Schematic representation of the equilibria involved in the interconversion between **Rot**H_2_^3+^, **Rot**H^2+^ (I and II), and **Rot**^+^.

The interconversion between the different protonated forms of the rotaxanes occurs through a network of acid–base equilibria, characterized by the acidity constants *K*_1_*–K*_4_, and the exchange between the dications **Rot**H^2+^-I and **Rot**H^2+^-II, regulated by the equilibrium constant *K*_ex_ (Figure 4). The value of *K*_ex_ was inferred from the NMR spectra, and the values of *K*_1_*–K*_4_ were calculated by solving the equations that describe the reaction network at equilibrium (Figure S47), using the spectrophotometric titration data with the phosphazene **B2** (Figures S45–S46) whose acidity constant in acetonitrile is known.^45^

The trication **Rot**H_2_^3+^ undergoes deprotonation to yield the two isomers **Rot**H^2+^-I and **Rot**H^2+^-II with p*K*_1_ = 23.5 and p*K*_2_ = 23.2, respectively; successive deprotonation of these two forms affords the monocation **Rot**^+^ with p*K*_3_ = 24.2 and p*K*_4_ = 24.5, respectively. It must be first emphasized that the supramolecular coordination of the rings in the rotaxane is necessary to express the difference between the acidity constants of the two ammonium sites.

These units are not chemically identical in the axle because of the uneven lengths of the alkyl bridges on either side of the triazolium cation and the orientation of the latter; in the free axle, however, the two monodeprotonated species are in fast exchange and cannot be individually detected (Figure S42). The ringless compound **9** therefore has to be treated as a simple diprotic acid whose dissociation constants were determined as p*K*_1_′ = 16.9 and p*K*_2_′ = 17.5 (Figure S46). Conversely, as noted in the previous paragraph, the two monodeprotonated forms of the rotaxane are in slow equilibrium on the ^1^H NMR time scale. Most likely, the proton exchange in the rotaxane is slow because (i) the removal of a proton from the complexed ammonium station is kinetically hindered, and/or (ii) the proton exchange between forms I and II involves the displacement of two rings (Figure 4). In the rotaxane, the two ammonium units not only become much harder to deprotonate because of the rings encircling them but also exhibit clearly different p*K*_a_ values; namely, the ammonium on the ethylene bridge side is about two times easier to deprotonate than that on the other side.

Another significant result is that the presence of a ring on the triazolium cation affects the acidity of the nearby complexed ammonium station, as, for instance, observed by comparing *K*_1_ and *K*_4_. Although the local chemical environment of the ammonium site on the methylene bridge side of **Rot**H_2_^3+^ and **Rot**H^2+^-II is the same, it can be argued that in **Rot**H^2+^-II the steric interaction between the ring on the triazolium and that on the ammonium disfavors the shuttling of the latter, thus making the station less acidic (*K*_4_ < *K*_1_). A similar consideration can be done for the ammonium station on the ethylene bridge side by comparing the values of *K*_2_ and *K*_3_. The fact that the magnitude of this effect (Δp*K*_a_ = 1.0 units for both ammonium sites) exceeds the p*K*_a_ difference of the two successive deprotonations of the free axle (Δp*K*_a_ = 0.6 units) highlights the peculiar role of the mobile macrocycles in the acid–base equilibrium network.

### Variable-Temperature NMR

**Figure 5:**
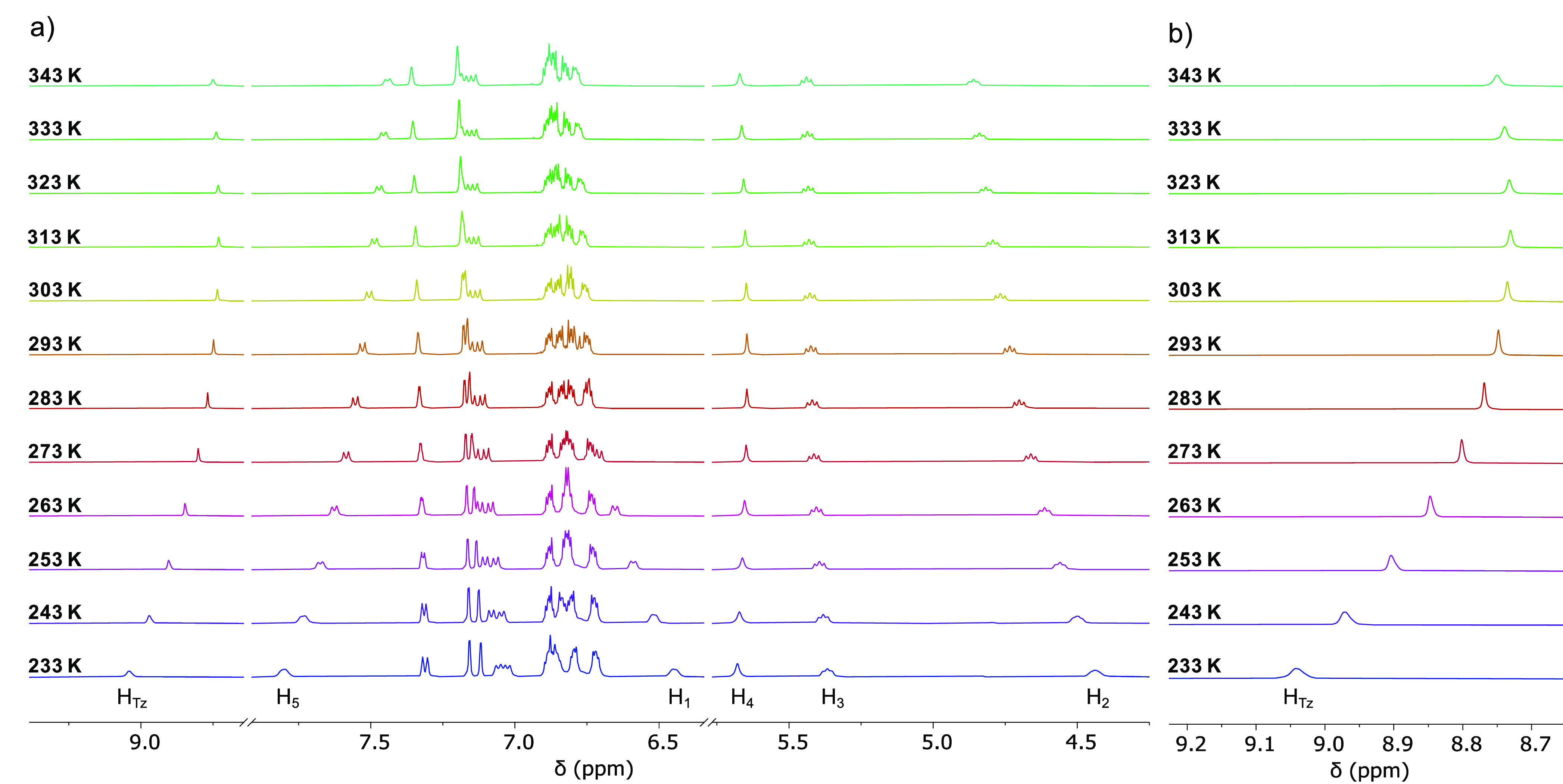
(a) Portion of the variable-temperature ^1^H NMR spectra of **Rot**^+^ (500 MHz, CD_3_CN). (b) Magnification of the chemical shift variation of the triazolium peak H_Tz_. For peak assignment refer to Figure 2.

The cation **Rot**^+^ is a unique rotaxane in which two crown ethers share a single triazolium station. We envisaged that the weak ion-dipole interaction holding the macrocycles close to the station would create a highly dynamic system; we therefore investigated the behavior of the crown ether rings in **Rot**^+^ by means of variable-temperature NMR analysis in acetonitrile (Figure 5 and S43). As a result of its anticipated dynamic nature, large spectral variations are observed in ^1^H NMR VT experiments performed on **Rot**^+^. In particular, the observed shielding upon temperature increase of the resonances on the methylene bridge side (H_5_), together with the deshielding of the resonances on the ethylene bridge side (H_1_, H_2_) are indicative of a concomitant shuttling of the rings toward the ethylene bridge. Additionally, the minor changes in chemical shift observed for the ethylene bridge protons H_3_ and the methylene bridge protons H_4_ imply that these atoms are subjected to a similar local environment at different temperatures. More insight is provided by the variation of the triazolium C–H peak (H_Tz_, Figure 5b): in contrast to the unidirectional trends mentioned above, the triazolium peak undergoes an initial shielding, shifting from 9.04 ppm at 233 K to 8.73 ppm at 313 K. An additional increase in temperature, however, does not lead to further shielding; instead, a low-field shift up to 8.76 ppm is observed at 343 K. Such unusual behavior is indicative of a multimodal dynamic system involving multiple coconformations, whose population distribution is temperature dependent. Similarly to the properties emerged from the deprotonation study, this peculiar temperature-dependent behavior arises from the displacement of the rings along the axle, as confirmed by the fact that variable-temperature experiments on the free monocationic axle display only unidirectional chemical shift variations (Figure S44). More information on this point was gained from molecular modeling studies.

### Molecular Modeling

Atomistic-level insight on the structure, the thermodynamics and the kinetics of the different [3]rotaxanes **Rot**H_2_^3+^, **Rot**H^2+^-I, **Rot**H^2+^-II, and **Rot**^+^ was provided by density functional theory (DFT) modeling.^46,47^ Whereas the instances where *n*_S_ ≥ *n*_R_ were tackled via geometry optimizations, the case where *n*_R_ > *n*_S_ (i.e., **Rot**^+^) was investigated via a finite temperature DFT-metadynamics approach (see the Supporting Information for an account of the computational approaches).^48−52^ Indeed, in the **Rot**^+^ case, a competition can be foretold for the two DB24C8 macrocycles to approach the only charged station in the axle, which could lead to a highly dynamic “frustrated” system.

We first analyzed the *n*_S_ ≥ *n*_R_ cases. In **Rot**H_2_^3+^ the rings are located on the two ammonium stations (Figure S49), in agreement with the experimental results. The two dicationic [3]rotaxanes, **Rot**H^2+^-I and **Rot**H^2+^-II, feature one macrocycle on the protonated Am station, whereas the other ring encircles the Tz station in the center of the axle (Figures S50 and S51). **Rot**H^2+^-II is more stable by 1.4 kcal mol^–1^ with respect to **Rot**H^2+^-I (≈2*k*T at room temperature). The calculated NMR chemical shifts for both dications are compared with those for the **Rot**H_2_^3+^ trication in Figure S52. The calculations reproduce fairly well the chemical shift trend of the triazolium proton H_Tz_ in the first deprotonation step (**Rot**H_2_^3+^ → **Rot**H^2+^-I and **Rot**H^2+^-II), confirming that the stable occupation of the Tz station by one DB24C8 macrocycle causes a significant change in the H_Tz_ chemical shift.

**Figure 6:**
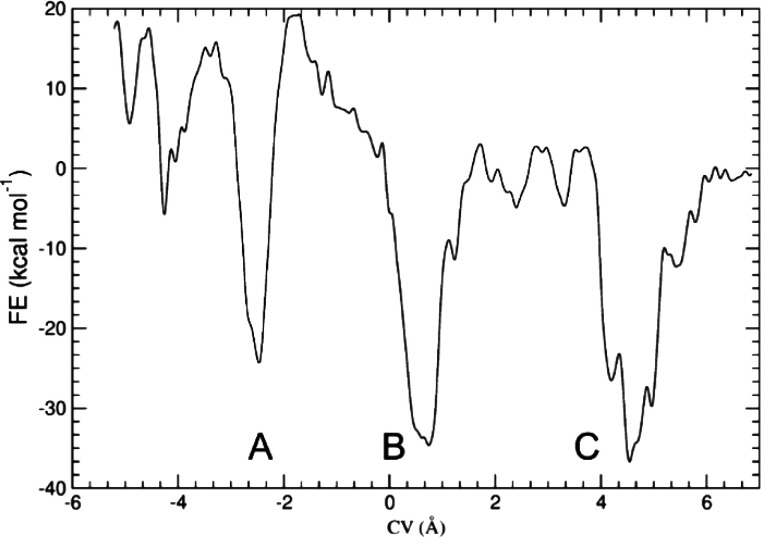
Free energy profile (kcal mol^–1^) for the shuttling of the two macrocycles along the axle in **Rot**^+^. The collective variable CV (in Å) represents the displacement of the ethereal oxygen atoms of the two rings with respect to the Tz nitrogen atoms (see the Supporting Information for details). The three different coconformations of **Rot**^+^ characterized by deep free energy minima are labeled A, B, and C.

We expect a more complex behavior for **Rot**^+^ because two macrocycles are competing for the sole station (Tz) remained on the axle. The free energy profile for the shuttling of the two macrocycles with respect to the central triazolium site is shown in Figure 6. Because of the large dimensions of the system, this profile was calculated in vacuum, hence the predicted barriers are higher than the real ones. However, previous studies on rotaxanes indicated that although the inclusion of solvent molecules decreases the barriers by about 15 kcal mol^–1^, the transition state structures do not change appreciably with respect to vacuum calculations.^53^ Therefore, although we acknowledge that all barriers are quantitatively overestimated, we are confident that the free energy path provides a qualitatively correct picture of the shuttling process. The profile displays three free energy wells with comparable depth characterized by a very different mutual arrangement of the molecular components.

**Figure 7:**
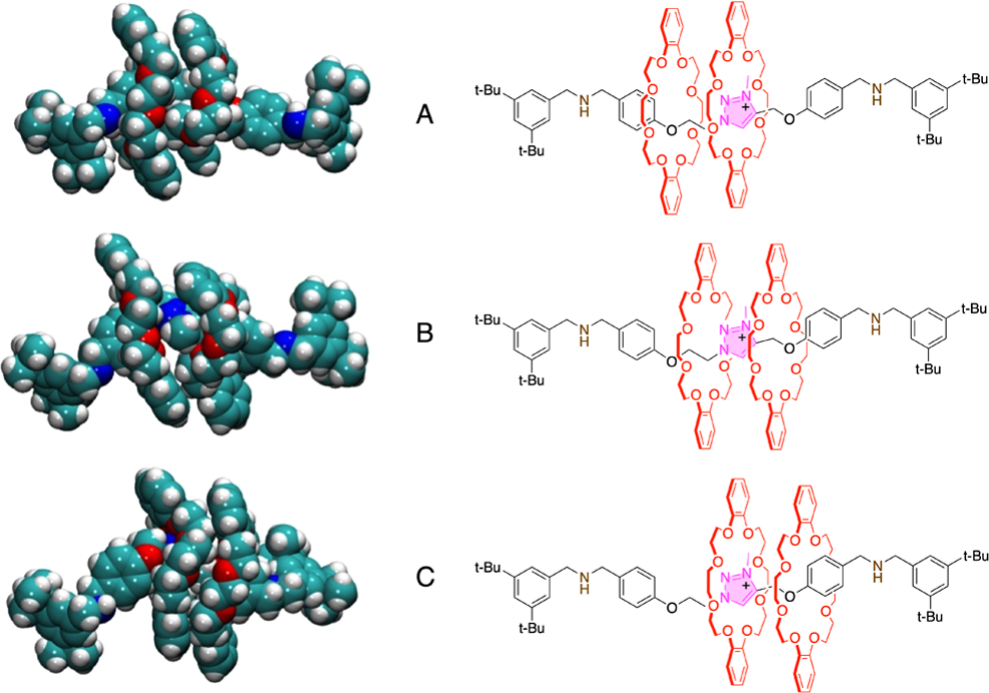
Graphical representations of the three coconformations extracted from the combined Metadynamics-DFT simulation of **Rot**^+^ at room temperature. The reported coconformations are representative of the three free energy minima labeled A, B, and C in Figure 6. Color code: C, cyan; N, blue; O, red; H, white. The corresponding structural formulas are also shown for clarity.

Typical structures representative of the three minima are shown in Figure 7. In structure A, one macrocycle is located close to the Tz station and the other one is on the ethylene bridge side. In structure B, both macrocycles are close to the central Tz station. In structure C, one DB24C8 ring is close to the central Tz station and the other ring sits on the methylene bridge side. The profile in Figure 6 highlights a significantly lower barrier for the B → C path with respect to the B → A one. Such a multiwell free energy profile can explain the unusual temperature-dependent behavior of **Rot**^+^ as detected by VT-NMR analysis.

At low temperatures, the most stable structure C is predominant, whereas at intermediate temperatures, both C and B are present in comparable concentrations. The population increase of structure A requires higher temperatures because A is less stable than B and C. This hypothesis is supported by the calculated chemical shift of the triazolium proton H_Tz_, which indicates a deshielding for structure C, a moderate shielding for structure B, and again, a deshielding for structure A (Figure S53), in agreement with the experimental VT-NMR data (Figure 5b). In fact, Figure 2 indicates that a macrocycle encircling the central Tz station like in **Rot**H^2+^-I and **Rot**H^2+^-II causes a pronounced deshielding of the triazolium proton H_Tz_. On the contrary, the chemical shift decreases when the Tz proton is far away from the ethereal oxygen atoms of a DB24C8 macrocycle, like in **Rot**H_2_^3+^ (Figure S49). In both A and C, a macrocycle is very close to the Tz station, hence the H_Tz_ proton is deshielded. Conversely, in structure B, both macrocycles are relatively distant from the Tz central station, and the signal calculated for the H_Tz_ proton is not deshielded.

**Figure 8:**
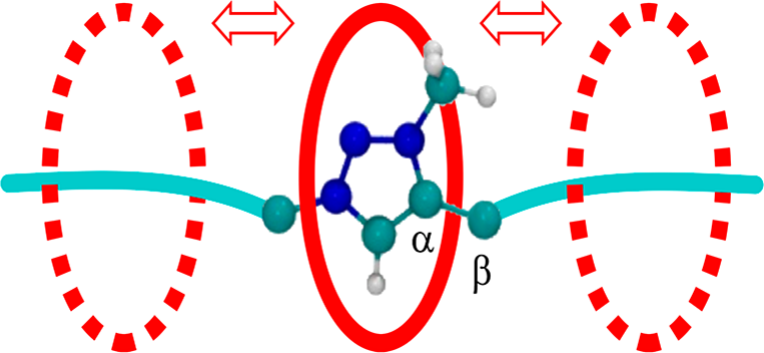
Ball-and-stick representation of a triazolium unit encircled by a macrocycle (red oval). Color code: C, cyan; N, blue; H, white. The red arrows represent two nonequivalent shuttling directions for the macrocycle.

The much higher barrier for the B → A path than for the B → C one suggests that directionally controlled shuttling motion could in principle be achieved in triazolium-based rotaxanes. Because of the intrinsic asymmetry of the Tz group, which can be approximated to a flat rigid section bearing an off-axis methyl, the macrocycle transit can occur by two nonequivalent paths (Figure 8).

**Figure 9:**
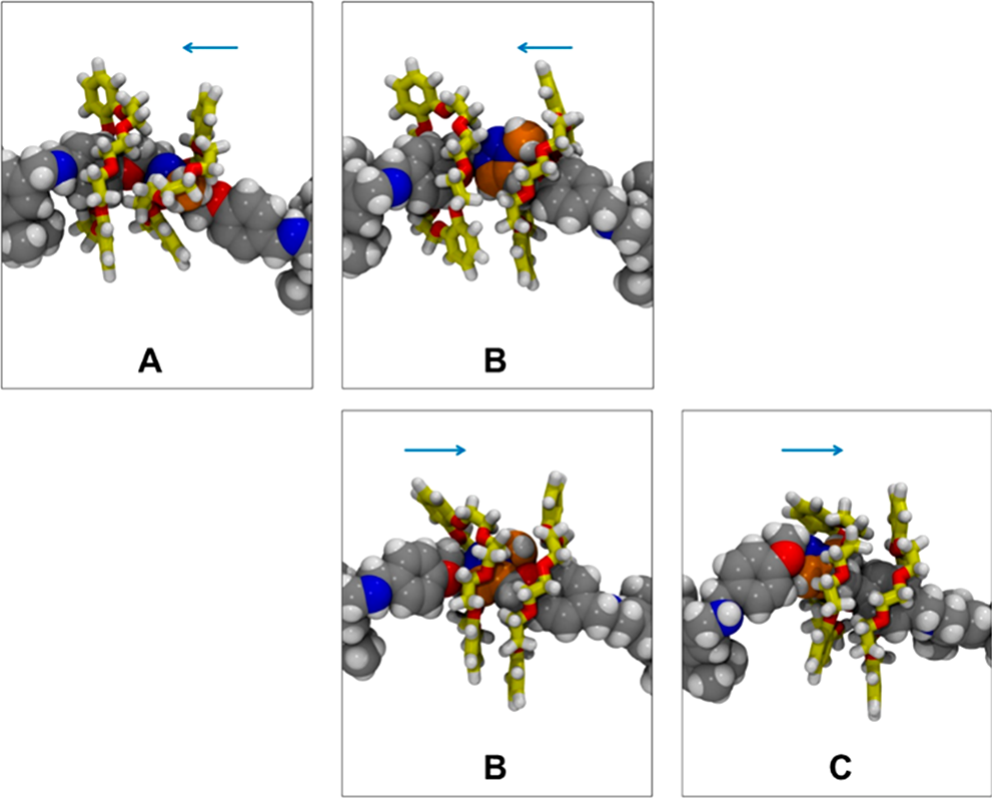
Snapshots taken from the DFT-metadynamics simulation of **Rot**^+^. Top panels: right-to-left transit of a DB24C8 macrocycle over the Tz station, describing the B → A path. Bottom panels: left-to-right transit of a DB24C8 macrocycle over the Tz station, describing the B → C path. Color code: H, white; N, blue; O, red. C atoms belonging to the axle in gray, C atoms belonging to the DB24C8 rings in yellow, C atoms of the triazolium group in orange. Atoms of the axle are represented as spheres, atoms of the macrocycles are represented as sticks.

The difference in free energy for the B → A transit with respect to the B → C one can be related to such an asymmetry. In particular, in the B → C case, a macrocycle should first pass over the flat rigid part of the Tz, thus hindering the Tz rotation (left-hand side in Figures 8 and 9). As a consequence, the passage is easier and the B → C transit can be completed at a lower free energy cost (Figure 9, lower panels). On the other hand, in the B → A case, the macrocycle should first impact with the bulkier methyl group, causing a rotation of the Tz along the C_α_-C_β_ single bond (right-hand side in Figures 8 and 9). As a result, the transit of the macrocycle is hampered by the Tz rotation and hence is accomplished at a greater free energy cost (Figure 9, upper panels).

Movies S1–S3 show that such a mechanism, previously detected for the transit of a DB24C8 macrocycle over a different pseudostopper,^53^ is operative also in the B → A path described here. The rotation of Tz during the transit is accompanied by low-energy synchronous vibrations of the crown ether ring, which provide the appropriate deformation needed to accomplish the shuttling.^53^ In particular, Movie S1 highlights that a rotation of nearly 90° of the Tz moiety occurs along the B → A path, whereas no similar rotation of the Tz group has been detected during the B → C transit (Movie S3). Finally, Movie S2 illustrates the proximity of the rings to share the complexation of the triazolium station.

## Conclusion

We have designed and investigated a [3]rotaxane that contains two acid–base-switchable ammonium sites and an acid–base-insensitive triazolium unit, giving access to a family of rotaxanes whose members – **Rot**H_2_^3+^, **Rot**H^2+^, and **Rot**^+^ – contain a number of recognition sites (*n*_S_) respectively larger than, equal to, or lower than the number of interlocked macrocyclic rings (*n*_R_).

Detailed spectroscopic studies and DFT-metadynamics calculations showed that these molecules exhibit markedly different structural and dynamic properties. In **Rot**H_2_^3+^, the two crown ethers encircle the ammonium stations, making them harder to deprotonate by a base in solution; moreover, they exhibit different p*K*_a_ values because of the inherent asymmetry of the axle. **Rot**H^2+^ can exist in two nonequivalent isomeric forms that differ for the position of the ammonium site (and of its surrounding macrocycle) with respect to the central triazolium. Although in the free axle these two isomers are in fast equilibrium and could not be individually observed, in the rotaxane the proton exchange between the isomers becomes slow due to the presence of the rings. Hence, the two forms of **Rot**H^2+^ could be studied, and the peculiar effect of the moving rings on the acidity of the ammonium sites has been thoroughly characterized.

The fully deprotonated species **Rot**^+^ has *n*_S_ < *n*_R_, that is, there is only one recognition site for two rings. Such an arrangement is very rare, despite the large number of studies on rotaxanes available in the literature. Our expectation that a situation of this kind could lead to a frustrated, highly dynamic system was confirmed by variable-temperature NMR studies. We employed computational modeling to identify the stable coconformations available to the rotaxane and to simulate their NMR spectra; this approach enabled us to interpret the nontrivial spectroscopic results and understand the dynamic properties of **Rot**^+^. Not unexpectedly, stable coconformations of this rotaxane consist of one macrocycle catching the triazolium station with the other ring located in the vicinity. However, a deep energy minimum in which the two crown ethers are very close together and share the recognition site was also identified. Another significant observation is that the barrier for the transit of a macrocycle over the triazolium unit is different depending on the approaching direction along the axle, because of the presence of the asymmetrically positioned methyl substituent.

This study shows that interesting and unusual thermodynamic and kinetic phenomena can emerge by interlocking multiple macrocyclic rings with a molecular axle and inducing a mismatch between rings and stations. The fact that the investigated compounds are based on simple molecular components (DB24C8 is commercially available) and a well-established chemistry makes them even more valuable^54^ and is a useful premise for extending the study to higher-order rotaxanes. Research in this direction is important to foster the use of MIMs with designed physicochemical and mechanical properties as components of functional nanostructured devices and materials.

## Supporting Information Available

Experimental and computational details, synthetic procedures, NMR and UV–visible spectra, and computational results (PDF)
Movie S1 showing a rotation of nearly 90° of the Tz moiety occuring along the B → A path, color code as in Figure 9 (MP4)
Movie S2 showing the proximity of the rings to share the complexation of the triazolium station, color code as in Figure 9 (MP4)
Movie S3 showing no rotation of the Tz group during the B → C transit, color code as in Figure 9 (MP4)
