# Supplementary material for: Chemically Induced Mismatch of Rings and Stations in [3]Rotaxanes
Source: J Am Chem Soc. 2021 Apr 29;143(21):8046–55. doi: 10.1021/jacs.1c02230 (PMC8176457; doi:10.1021/jacs.1c02230)
Supplement: Supplementary file 1 — ja1c02230_si_001.pdf [file ja1c02230_si_001.pdf]

Chemically Induced Mismatch of Rings and Stations in [3]Rotaxanes

Massimiliano Curcio,[a,b] Federico Nicoli,[a,b] Erica Paltrinieri,[a,b] Ettore Fois,[c,*] Gloria Tabacchi,[c] Luigi Cavallo,[d] Serena Silvi,[b,e] Massimo Baroncini,[b,f] and Alberto Credi[a,b,*]

[a] *Dipartimento di Chimica Industriale "Toso Montanari", Università di Bologna, Bologna, Italy.*

[b] *Center for Light Activated Nanostructures, Istituto ISOF-CNR, Bologna, Italy.*

[c] *Dipartimento di Scienza e Alta Tecnologia, Università dell'Insubria, Como, Italy.*

[d] *Kaust Catalysis Center, Physical Sciences and Engineering Division, King Abdullah University of Science and Technology, Thuwal, Saudi Arabia.*

[e] *Dipartimento di Chimica "Giacomo Ciamician", Università di Bologna, Bologna, Italy.*

[f] *Dipartimento di Scienze e Tecnologie Agro-alimentari, Università di Bologna, Bologna, Italy.*

SUPPORTING INFORMATION

Table of contents

1. Experimental Details **3**

2. Computational Details **4**

3. Synthetic procedures **6**

Synthetic pathway to **1** 6
Synthesis of **2** 7
Synthesis of **3** 7
Synthesis of **4** 8
Synthesis of **5** 8
Synthesis of **6** 8
Synthesis of **7** 9
Synthesis of **Rot**H_2^{3+} 10
Rotaxane deprotonation procedure 11
Synthesis of **Rot**$^+$ 12
Synthesis of **8** 13
Synthesis of **9** 14
Synthesis of **10** 14

4. NMR spectra **15**

5. Spectrophotometric data **36**

UV-Visible titration of **Rot**H_2^{3+} 36
UV-Visible titration of **9** 36

6. Thermodynamic analysis **37**

7. Computational results **38**

Starting co-conformation of **Rot**$^+$ for MTD runs 38
Optimized geometry of **Rot**H_2^{3+} 39
Optimized geometry of **Rot**H^{2+}-I 39
Optimized geometry of **Rot**H^{2+}-II 40
Simulated NMR spectra 41
Co-conformation of **Rot**$^+$ from energy minimum A 42
Co-conformation of **Rot**$^+$ from energy minimum B 43
Co-conformation of **Rot**$^+$ from energy minimum C 43

References **44**

Experimental Details

Solvents and reagents [3,5-di-*tert*-butyl-benzyl bromide, sodium azide, 4-hydroxybenzaldehyde, sodium borohydride, di-*tert*-butyl-dicarbonate, 1,2-dibromoethane, propargyl bromide, hexafluorophosphoric acid, dibenzo-24-crown-8, methyl iodide, ammonium hexafluorophosphate, polystyrene-supported BEMP, P1-*t*-Bu] and triethylamine were all used as supplied by Fluorochem, Sigma-Aldrich or VWR without further purification.

^{1}H NMR spectra were recorded on an Agilent DD2 spectrometer operating at 500 MHz or a Varian Mercury spectrometer operating at 400 MHz; ^{13}C NMR spectra were recorded on an Agilent DD2 spectrometer operating at 126 MHz or a Varian Mercury spectrometer operating at 101 MHz. Chemical shifts are quoted in ppm relative to tetramethylsilane ($SiMe_4$, δ = 0 ppm), using the residual solvent peak as a reference standard; all coupling constants (*J*) are expressed in Hertz (Hz). Exchange rate constant quantification was carried out using the MestreLab EXSYCalc software.

Flash column chromatography was performed using Sigma Aldrich Silica 40 (230-400 mesh size or 40-63 µm) as the stationary phase. Size exclusion chromatography was performed using Biorad Biobeads SX-3 as the stationary phase. Thin layer chromatography was performed on TLC Silica gel 60 F254 coated aluminium plates from Merck. Hydrogenation reactions were carried out in a H-Cube flow reactor equipped with a 10 % Pd/C catalyst at a H_2 pressure of 10 bar and a flow rate of 1 mL min^{-1} using methanol as the solvent.

Deprotonation experiments were carried out in acetonitrile using triethylamine (pK_a = 18.8) or phosphazene compounds as heterogeneous [**B1,** polystyrene-supported BEMP] and homogeneous [**B2,** P1-*t*-Bu), pK_a = 26.9] bases. The pK_a values for the ammonium stations were calculated based on the titration curve fitting, carried out on the HyperSpec suite. The error on the pK_a values is estimated to be ±0.1 units, calculated as the average mean square root in the pK_a values of the ammonium stations investigated.

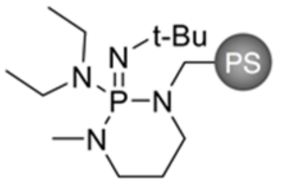

B1

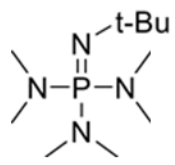

B2

Computational Details

Quantum chemical calculations

A model structure of the complex **Rot**H_2^{3+}, consisting of two identical dibenzo-24-crown-8 (DB24C8) rings interlocked with an axle containing two lateral dibenzylammonium (Am) and one central triazolium (Tz) stations, was optimized by adopting the D95(d,p)[1] basis set and the range-separated hybrid functional ωB97XD[2] as density functional approximation to DFT.

From this optimized structure, two different starting geometries for the **Rot**H^{2+} model were created by removing one proton from one of the two lateral dibenzylammonium stations. These computational models correspond to the two isomers **Rot**H^{2+}-I and **Rot**H^{2+}-II, formed upon mono-deprotonation of the parent compound **Rot**H_2^{3+}. In particular, the starting structures for **Rot**H^{2+}-I and **Rot**H^{2+}-II were obtained by deprotonating the Am on the methyl group side, and the Am on the ethyl group side, respectively. These two geometries were optimized at the same level of theory as **Rot**H_2^{3+} - namely, ωB97XD/D95(d,p).

The calculations of the NMR spectra of the above-mentioned optimized structures of **Rot**H_2^{3+} **Rot**H^{2+}-I and **Rot**H^{2+}-II, were performed by using as basis set a QZP (quadruple-ζ with polarization) for all the protons[3] and cc-pCVDZ (double-ζ with polarization and tight-core) for the heavy atoms[4] (O, C, N) and adopting a DFT approximation optimized for proton NMR chemical shifts[5]. The chemical shifts of all the protons were calculated by taking as a reference the chemical shift of the protons of tetrametilsilane (TMS). The chemical shifts of the protons reported in this work were calculated from the average over equivalent protons.

The above calculations were performed with the Gaussian 09 code[6] using an implicit solvent model for CH_2Cl_2 or CH_3CN.[7]

Ab initio molecular dynamics and Metadynamics calculations

The finite-temperature behavior of **Rot**$^+$ was modelled via *ab initio* molecular dynamics,[8] combined with statistical sampling according to the *ab initio* metadynamics[9,10] scheme.

A Generalized Gradient Approximation to density functional theory (DFT) was used to describe electron-electron interactions – in particular, the PBE functional in combination with empirical dispersion corrections (*i.e.* PBE-D2).[11,12] Ion cores-electron interactions were treated with ultra-soft pseudopotentials.[13] Plane-waves (PW) were used as basis set. The cutoffs for the PW expansion of the wavefunctions and density were 25 Ry and 200 Ry, respectively. Calculations were performed using periodic boundary conditions, which were applied to a simulation cell of size 70×30×30 Å. Such a size is sufficiently large to allow for a Γ-point-only sampling, and to minimize interactions of the rotaxane with periodic images.

Each simulation system was constituted by the neutral ring DB24C8 and by the positively charged axle. In all cases, the simulation cell contained a total of 255 atoms.

A guess configuration was obtained by removing one proton from both the two lateral dibenzylammonium stations of the parent compound. A*b initio* molecular dynamics (AIMD) equilibration (elapsed time: 10 ps) was performed at 300K (27 °C). This temperature was chosen in order to favour a faster equilibration of the system. Additionally, it corresponds roughly to the central part of the temperature conditions at which the variable temperature NMR experiments were conducted (T = from –40 to 70 °C).

The simulations were carried out in the canonical NVT ensemble and with Nose-Hoover chain thermostats for the ionic degrees of freedom.[14,15] The AIMD equations[8,16] were integrated with a time step of 5 atomic units (a.u.), *i.e.* 0.121 fs. The (fictitious) mass of the wavefunction's coefficients was 500 a.u.

The shuttling process was explored by performing two *ab initio* metadynamics (MTD). In the first run, we selected as collective variable (CV), the displacement of the 8 oxygen atoms of the DB24C8 macrocycle on the side of the ethylene bridge, with respect to the nitrogen atoms of the Tz station (see path B→ C in Figure 10, main text). In the second run, we adopted as CV the displacement of the 8 oxygen atoms of the ring on the methylene bridge side with respect to the Tz nitrogen atoms (see path B→A in Figure 10, main text). For the evolution of the CV, we employed the Lagrange-Langevin dynamics with friction of 0.001 a.u. The selected target temperature was 300 K as in the equilibration runs. The metadynamics parameters adopted for the gaussian hills in the production simulations were the following in all the runs: perpendicular width = 0.02 a.u., height = 0.002 a.u. The sampling was accomplished in ~3000 metadynamics steps.

Due to the size of the simulation cell, all the PW simulations were performed in the gas phase.

All PW calculations were carried out with the CPMD (Car-Parrinello-Molecular-Dynamics) computer program[17] running on the Shaheen II supercomputer at Kaust.

Synthetic procedures

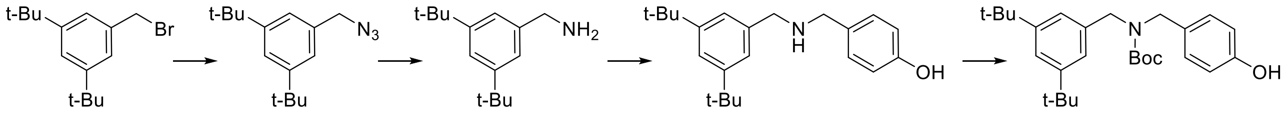

a ***b*** ***c*** ***d*** **1**

Figure S1. Synthetic route to the Boc-protected intermediate **1**.

3,5-di-*tert*-butylbenzyl azide, *b*

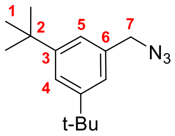

A solution of 3,5-di-*tert*-butylbenzyl bromide ***a*** (10.34 g, 36.50 mmol) and sodium azide (2.37 g, 36.50 mmol) in dimethylformamide (200 mL) was heated to 70 °C for 2 h. Cooling to room temperature and removal of the solvent under reduced pressure provided a crude solid which was suspended in diethyl ether (100 mL), washed with water (3×100 mL) and dried over anhydrous $MgSO_4$. Filtration and removal of the solvent under reduced pressure provided the azide ***b*** as a pale yellow oil (8.96 g, quantitative), which was used without further purification. ^{1}H NMR (500 MHz, Chloroform-*d*) δ 7.47 (t, *J* = 1.9 Hz, 1H, **4**), 7.20 (d, *J* = 1.9 Hz, 2H, **5**), 4.39 (s, 2H, **7**), 1.40 (s, 18H, **1**). ^{13}C NMR (126 MHz, Chloroform-*d*) δ 151.51, 134.68, 122.51, 122.42, 55.62, 34.96, 31.55.

3,5-di-*tert*-butylbenzyl amine, *c*

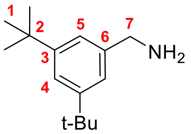

A solution of the azide ***b*** (8.96 g, 36.50 mmol) in methanol (730 mL) was reduced using a flow hydrogenation reactor equipped with a 10 % Pd/C catalyst and in situ generated H_2 (10 bar) at a flow rate of 1 mL min^{-1}. Removal of the solvent under reduced pressure provided the product ***c*** as a colourless oil (8.01 g, quantitative), which was used without further purification. ^{1}H NMR (500 MHz, Chloroform-*d*) δ 7.34 (t, *J* = 1.9 Hz, 1H, **4**), 7.17 (d, *J* = 1.9 Hz, 2H, **5**), 3.88 (s, 2H, **7**), 1.35 (s, 18H, **1**). ^{13}C NMR (126 MHz, Chloroform-*d*) δ 150.99, 142.53, 121.29, 120.90, 47.18, 34.86, 31.51.

4-(((3,5-di-*tert*-butylbenzyl)amino)methyl)phenol, *d*

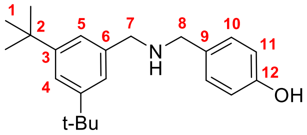

An ethanol solution (200 mL) of ***c*** (3.11 g, 14.16 mmol) and 4-hydroxybenzaldehyde (1.73 g, 14.16 mmol) was dried azeotropically at the rotary evaporator. The procedure was repeated three times, affording the imine intermediate, which was re-dissolved in ethanol (200 mL) and reduced by portionwise addition of $NaBH_4$ (1.18 g, 17.00 mmol) providing a vigorous effervescence and a final light-yellow solution. Once the effervescence terminated, the solution was heated to reflux for 30 min, then cooled down to room temperature and the solvent removed under reduced pressure. The crude product was dissolved in ethyl acetate (150 mL), washed with water (3×150 mL) and dried over $MgSO_4$. Filtration and removal of the solvent under reduced pressure provided an off-white solid which was purified by recrystallisation from

cyclohexane to obtain the product ***d*** as a colourless solid (3.79 g, 82%). ^{1}H NMR (400 MHz, Chloroform-*d*) δ 7.34 (t, *J* = 1.8 Hz, 1H, **4**), 7.18 (d, *J* = 1.8 Hz, 2H, **5**), 7.13 - 7.07 (m, 2H, **10**), 6.63 - 6.57 (m, 2H, **11**), 3.85 (s, 2H, **8**), 3.76 (s, 2H, **7**), 1.31 (s, 18H, **1**). ^{13}C NMR (101 MHz, Chloroform-*d*) δ 156.24, 151.13, 138.15, 130.04, 129.94, 122.79, 121.44, 115.99, 53.86, 52.83, 34.94, 31.59.

***tert*-butyl 3,5-di-*tert*-butylbenzyl(4-hydroxybenzyl)carbamate, 1**

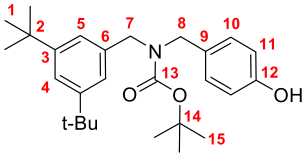

A tetrahydrofuran solution (100 mL) of di-*tert*-butyl dicarbonate (2.65 mL, 11.52 mmol) was added dropwise to a tetrahydrofuran solution (500 mL) of ***d*** (3.75 g, 11.52 mmol). The resulting mixture was stirred at room temperature for 16 h. Upon reaction completion, the solvent was removed under reduced pressure and the residue dissolved in ethyl acetate (150 mL), washed with water (3×150 mL) and dried over $MgSO_4$. Filtration and removal of the solvent under reduced pressure provided a yellow solid which was purified by flash chromatography (hexane:ethyl acetate 70:30, R_f = 0.65) to obtain the product **1** as a colourless solid (4.275 g, 87%). ^{1}H NMR (400 MHz, Chloroform-*d*) δ 7.33 (t, *J* = 1.7 Hz, 1H, **4**), 7.05 (m, 4H, **5+10**), 6.79 (m, 2H, **11**), 6.29 (s, 1H, O*H*), 4.34 (dd, *J* = 20.9, 18.6 Hz, 4H, **7+8**), 1.53 (s, 9H, **15**), 1.32 (s, 18H, **1**). ^{13}C NMR (101 MHz, Chloroform-*d*) δ 155.62, 150.91, 137.10, 136.70, 129.41, 129.10, 128.91, 122.29, 121.91, 121.18, 115.51, 80.34, 49.91, 49.41, 48.90, 48.68, 34.79, 31.50, 28.56.

***tert*-butyl 3,5-di-*tert*-butylbenzyl(4-(prop-2-yn-1-yloxy)benzyl)carbamate, 2**

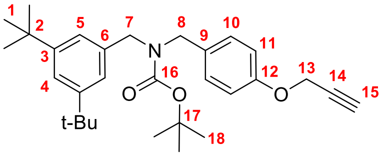

A dry acetonitrile (250 mL) suspension containing compound **1** (1.20 g, 2.82 mmol), propargyl bromide (911 µL, 8.46 mmol) and K_2CO_3 (780 mg, 5.64 mmol) was stirred at 50 °C for 96 h under a dinitrogen atmosphere. The mixture was then cooled down to room temperature and the solvent removed under reduced pressure. The residue was dissolved in dichloromethane (100 mL), washed with water (3×100 mL) and dried over $MgSO_4$. Filtration and removal of the solvent under reduced pressure provided an orange oil which was purified by flash chromatography (hexane:ethyl acetate 85:15, R_f = 0.37) to obtain the product **2** as a brown/orange oil (1.23 g, 94%). ^{1}H NMR (400 MHz, Chloroform-*d*) δ 7.32 (t, *J* = 1.8 Hz, 1H, **4**), 7.22 - 6.98 (m, 4H, **5+10**), 6.93 (d, *J* = 8.6 Hz, 2H, **11**), 4.69 (d, *J* = 2.4 Hz, 2H, **13**), 4.47 - 4.17 (m, 4H, **7+8**), 2.52 (t, *J* = 2.4 Hz, 1H, **15**), 1.51 (s, 9H, **18**), 1.31 (s, 18H, **1**). ^{13}C NMR (101 MHz, Chloroform-*d*) δ 156.91, 156.10, 150.97, 137.39, 137.12, 131.38, 129.51, 128.95, 122.41, 122.01, 121.23, 114.99, 79.93, 78.75, 75.62, 55.97, 50.04, 49.55, 48.90, 48.63, 34.90, 31.61, 28.63.

***N*-(3,5-di-*tert*-butylbenzyl)-1-(4-(prop-2-yn-1-yloxy)phenyl)methanaminium hexafluorophosphate, 3**

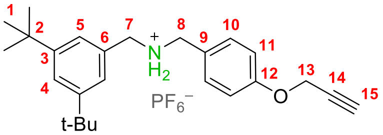

A tetrahydrofuran solution (1 mL) of HPF_6 (1.3 mL of a 55% aqueous solution, 9.00 mmol) was added to a tetrahydrofuran solution (10 mL) of compound **2** (1.40 g, 3.02 mmol) and the resulting mixture stirred at room temperature for 4 h. The

solvent was removed under reduced pressure, water (50 mL) was then added and the mixture was extracted with dichloromethane (3×50 mL) and the combined organic phases washed with water (3×50 mL). The solvent was removed under reduced pressure providing a light brown crude product that was purified by recrystallisation from cyclohexane, obtaining the product **3** as a colourless solid (1.15 g, 75%). ^{1}H NMR (500 MHz, Chloroform-*d*) δ 7.47 (s, 1H, **4**), 7.30 - 7.25 (m, 2H, **10**), 7.17 (d, *J* = 1.8 Hz, 2H, **5**), 6.98 (d, *J* = 8.5 Hz, 2H, **11**), 4.63 (d, *J* = 2.1 Hz, 2H, **13**), 4.11 (d, *J* = 21.6 Hz, 4H, **7+8**), 2.51 (m, 1H, **15**), 1.31 (s, 18H, **1**). ^{13}C NMR (126 MHz, Chloroform-*d*) δ 158.88, 152.67, 131.59, 128.40, 124.25, 124.04, 122.12, 115.91, 78.07, 76.14, 55.91, 51.77, 50.52, 35.10, 31.39. ^{19}F NMR (470 MHz, Chloroform-*d*) δ – 0.47 (d, *J* = 715.4 Hz, 6F).

***tert*-butyl 4-(2-bromoethoxy)benzyl(3,5-di-*tert*-butylbenzyl)carbamate, 4**

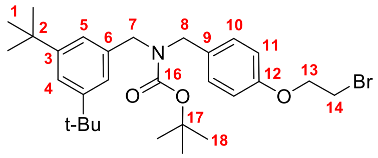

Compound **1** (1.57 g, 3.68 mmol), 1,2-dibromoethane (5 mL, 58 mmol), KI (30 mg, 0.18 mmol) and K_2CO_3 (1.27 g, 9.20 mmol) were suspended in dry acetonitrile (10 mL) under a dinitrogen atmosphere and stirred at 70 °C for 5 days. The mixture was then cooled down to room temperature and ethyl acetate (20 mL) was added. The organic phase was washed with water (3×50 mL), dried over $MgSO_4$. Filtration and removal of the solvent under reduced pressure provided a pale yellow solid which was purified by flash chromatography (hexane:ethyl acetate 90:10) to obtain the product **4** as a colourless solid (820 mg, 42%). ^{1}H NMR (400 MHz, Chloroform-*d*) δ 7.32 (s, 1H, **4**), 7.24 - 6.96 (m, 4H, **5+10**), 6.86 (d, *J* = 8.4 Hz, 2H, **11**), 4.44 - 4.23 (m, 6H, **7+8+14**), 3.64 (t, *J* = 6.3 Hz, 2H, **13**), 1.51 (s, 9H, **17**), 1.31 (s, 18H, **1**). ^{13}C NMR (126 MHz, Chloroform-d) δ 157.28, 155.98, 150.84, 137.25, 131.13, 129.51, 128.91, 122.26, 121.87, 121.10, 114.75, 79.82, 67.94, 49.89, 49.42, 48.47, 34.77, 31.47, 29.10, 28.50.

***tert*-butyl 4-(2-azidoethoxy)benzyl(3,5-di-*tert*-butylbenzyl)carbamate, 5**

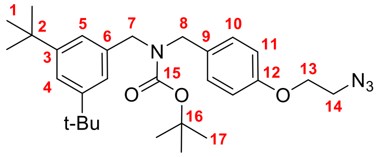

Compound **4** (1.28 g, 2.40 mmol) and sodium azide (200 mg, 3.10 mmol) were suspended in dry dimethylformamide (20 mL) under a dinitrogen atmosphere and stirred at 70 °C for 3 h. The mixture was then cooled down to room temperature and water (20 mL) was added, followed by extraction with diethyl ether (3×30 mL). The combined organic phases were washed with water (3×30 mL) and dried over $MgSO_4$. Filtration and removal of the solvent under reduced pressure provided the product **5** as a colourless solid (980 mg, 83%). ^{1}H NMR (500 MHz, Chloroform-*d*) δ 7.33 (s, 1H, **4**), 7.25 - 6.97 (m, 4H, **5+10**), 6.88 (d, *J* = 8.4 Hz, 2H, **11**), 4.46 - 4.24 (m, 4H, **7+8**), 4.16 (t, *J* = 5.0 Hz, 2H, **14**), 3.61 (t, *J* = 4.9 Hz, 2H, **13**), 1.52 (s, 9H, **17**), 1.32 (s, 18H, **1**). ^{13}C NMR (126 MHz, Chloroform-*d*) δ 157.58, 156.13, 150.99, 137.40, 131.18, 129.62, 128.99, 122.43, 122.06, 121.26, 114.72, 79.97, 67.17, 50.34, 50.02, 49.56, 48.92, 48.62, 34.92, 31.62, 28.65.

***N*-(4-(2-azidoethoxy)benzyl)-1-(3,5-di-*tert*-butylphenyl)methanaminium hexafluorophosphate, 6**

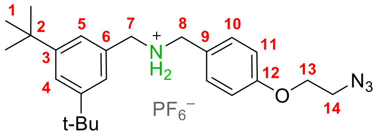

A tetrahydrofuran solution (1 mL) of HPF_6 (1.3 mL of a 55% aqueous solution, 9.00 mmol) was added to a tetrahydrofuran solution (10 mL) of compound **5** (930 mg, 1.90 mmol) and the resulting mixture stirred at room temperature for 4 h. Water (50 mL) was then added and the mixture was extracted with dichloromethane (3×50 mL) and the combined organic phases washed with water (3×50 mL). The solvent was removed under reduced pressure providing a light brown crude product that was purified by recrystallisation from cyclohexane, obtaining the product **6** as a colourless solid (741 mg, 72%). ^{1}H NMR (500 MHz, Chloroform-*d*) δ 7.47 (s, 1H, **4**), 7.30 - 7.25 (m, 2H, **10**), 7.18 (m, 2H, **5**), 6.92 (d, *J* = 8.5 Hz, 2H, **11**), 4.18 - 4.06 (m, 6H, **7**+**8**+**14**), 3.57 (t, *J* = 4.8 Hz, 2H, **13**), 1.31 (s, 18H, **1**). ^{13}C NMR (126 MHz, Chloroform-*d*) δ 159.62, 152.72, 131.69, 128.36, 124.29, 124.00, 121.86, 115.58, 67.10, 51.83, 50.63, 50.18, 35.10, 31.38. ^{19}F NMR (470 MHz, Chloroform-*d*) δ –70.47 (d, *J* = 715.4 Hz, 6F).

Triazole-linked [3]rotaxane, 7

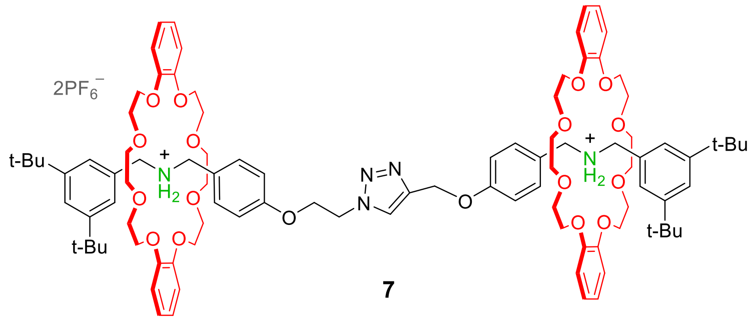

7

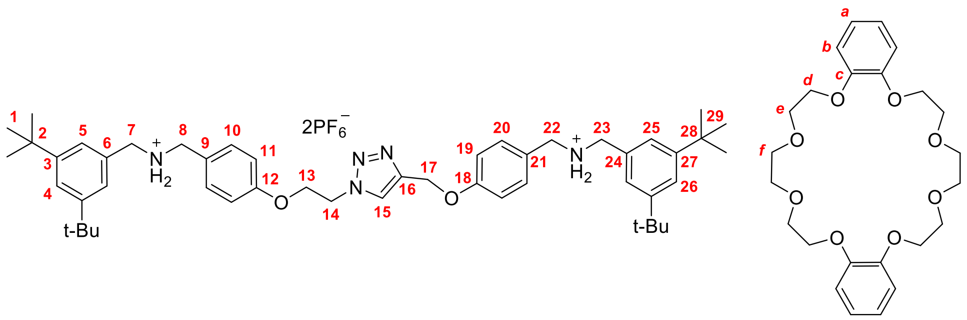

Compound **6** (150 mg, 0.28 mmol), compound **3** (143 mg, 0.28 mmol) and DB24C8 (630 mg, 1.40 mmol) were dissolved in dry dichloromethane (20 mL) and stirred for 10 min at room temperature under a dinitrogen atmosphere. The catalyst $[Cu(MeCN)_4][PF_6]$ (59 mg, 0.61 mmol) was then added and the resulting mixture stirred at room temperature for 48 h. The mixture was then diluted with dichloromethane (50 mL) and washed with sodium ethylenediaminotetraacetate (0.1 M in H_2O, 3×25 mL), water (3×25 mL) and dried over $MgSO_4$. Filtration and removal of the solvent under reduced pressure provided a pale-yellow solid which was purified by Size-Exclusion Chromatography (dichloromethane) to obtain the product **7** as a colourless solid (425 mg, 78%). ^{1}H NMR (500 MHz, Methylene Chloride-d_2) δ 7.94 (s, 1H, **15**), 7.53 (s, 4H, NH_2), 7.42 - 7.37 (m, 2H, **4**+**26**), 7.29 (m, 4H, **5**+**25**), 7.12 (d, *J* = 8.7 Hz, 2H, **20**), 7.11 (d, *J* = 8.7 Hz, 2H, **10**), 6.92 - 6.80 (m, 8H, ***a***), 6.84 - 6.75 (m, 8H, ***b***), 6.67 (d, *J* = 8.6 Hz, 2H, **19**), 6.53 (d, *J* = 8.6 Hz, 2H, **11**), 5.02 (s, 2H, **17**), 4.76 - 4.68 (m, 6H, **14**+**7**+**23**), 4.52 (dd, *J* = 6.8, 3.2 Hz, 4H, **8**+**22**), 4.20 (t, *J* = 5.1 Hz, 2H, **13**), 4.14 - 4.03 (m, 16H, ***d***), 3.83 - 3.68 (m, 16H, ***e***), 3.66 - 3.43 (m, 16H, ***f***), 1.20 (s, 36H, **1**+**29**). ^{13}C NMR (126 MHz, Methylene Chloride-d_2) δ 159.33 (**18**), 158.80 (**12**), 151.94 (**3** or **26**), 151.90 (**26** or **3**), 147.88 and 147.82 (***c***), 143.77 (**16**), 131.85 (**6**+**24**), 131.23 (**10** or **20**), 131.21 (**20** or **10**), 124.83 (**15**), 124.48 (**9** or **21**), 124.10 (**21** or **9**), 123.96 (**5** or **25**), 123.92 (**25** or **5**), 123.71 (**4**+**27**), 121.94 and 121.86 (***a***), 114.86 (**19**), 114.59 (**11**), 112.93 and 112.85 (***b***), 71.01 and 70.98 (***f***), 70.61 and 70.60 (***e***), 68.36 and 68.28 (***d***), 66.54 (**13**), 61.91 (**17**), 53.22 (**7**+**23**), 52.70 and 52.60 (**8**+**22**), 50.01 (**14**), 35.15 and 35.14 (**2**+**28**), 31.46 and 31.45 (**1**+**29**). ^{19}F NMR (470 MHz, Acetonitrile-d_3) δ –72.89 (d, *J* = 706.5 Hz, 12F). HRMS-ESI (*m*/*z*): calcd for $[C_{97}H_{133}N_5O_{18}]$, 828.4862; found 828.9901 $[7+H]^{2+}$.

Tricationic [3]rotaxane, RotH_2^{3+}

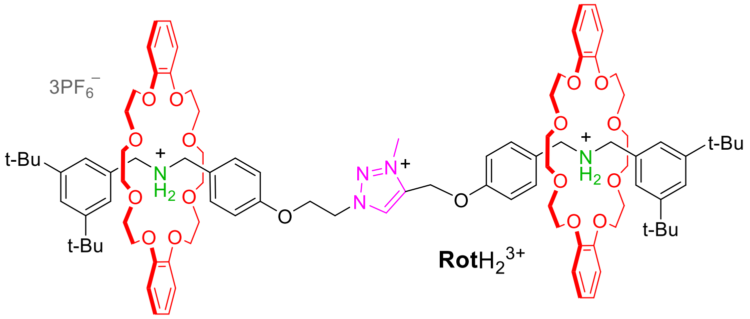

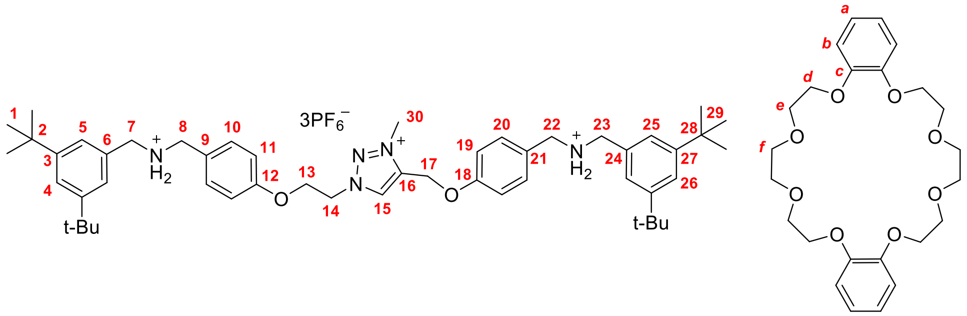

Under a dinitrogen atmosphere, compound **7** (254 mg, 0.13 mmol) was dissolved in iodomethane (13 mL) and the resulting solution was stirred for 48 h at room temperature under exclusion of light. Upon reaction completion, the solvent was removed under reduced pressure providing a yellow solid, which was dissolved in the minimum amount of acetonitrile. Addition of a saturated aqueous solution of ammonium hexafluorophosphate led to the precipitation of a yellow solid, which was isolated, dissolved in dichloromethane and washed with a saturated aqueous solution of ammonium hexafluorophosphate (3×5 mL) and water (3×5 mL) and dried over $MgSO_4$. Filtration and removal of the solvent under reduced pressure provided the product **Rot**H_2^{3+} as a yellow solid (246 mg, 91%). ^{1}H NMR (500 MHz, Methylene Chloride-*d*$_2$) δ 8.59 (s, 1H, **15**), 7.58 (s, 4H, N*H*$_2$), 7.41 (d, *J* = 6.9 Hz, 2H, **4**+**26**), 7.35 - 7.27 (m, 4H, **5**+**25**), 7.20 (d, *J* = 8.6 Hz, 2H, **20**), 7.14 (d, *J* = 8.6 Hz, 2H, **10**), 6.85 (m, 16H, ***a***+***b***), 6.69 (d, *J* = 8.6 Hz, 2H, **19**), 6.61 (d, *J* = 8.6 Hz, 2H, **11**), 5.19 (s, 2H, **17**), 4.99 - 4.93 (t, *J* = 5.1 Hz, 2H, **14**), 4.75 - 4.68 (m, 4H, **7**+**23**), 4.61 - 4.50 (m, 4H, **8**+**22**), 4.36 (s, 3H, **30**), 4.34 - 4.30 (t, *J* = 5.1 Hz, 2H, **13**), 4.10 (dtd, *J* = 15.9, 10.9, 6.3 Hz, 16H, ***d***), 3.77 (ddt, *J* = 41.8, 11.7, 6.1 Hz, 16H, ***e***), 3.68 - 3.44 (m, 16H, ***f***), 1.21 (d, *J* = 4.5 Hz, 36H, **1**+**29**). ^{13}C NMR (126 MHz, Methylene Chloride-*d*$_2$) δ 158.23 (**12**), 157.75 (**18**), 151.95 (**3** or **26**), 151.90 (**26** or **3**), 147.84 (***c***), 140.31 (**16**), 131.79 (**6**+**24**), 131.53 (**20**), 131.29 (**10**), 130.49 (**15**), 125.74 (**21**), 125.06 (**9**), 123.96 (**5** or **25**), 123.92 (**25** or **5**), 123.71 (**4**+**27**), 121.89 (***a***), 114.93 (**11**), 114.84 (**19**), 112.93 (***b***), 70.98 (***f***), 70.60 (***e***), 68.30 (***d***), 64.96 (**13**), 58.31 (**17**), 53.97 (**14**), 53.25 (**7**+**23**), 52.57 (**8**), 52.48 (**22**), 39.24 (**30**), 35.14 (**2**+**28**), 31.45 (**1**+**29**). ^{19}F NMR (470 MHz, Acetonitrile-*d*$_3$) δ –72.83 (d, *J* = 706.6 Hz, 18F). HRMS-ESI (*m/z*): calcd for $[C_{98}H_{136}N_5O_{18}P_2F_{12}]$, 1960.9164; found 1960.9164 [(**Rot**H_2)$(PF_6)_2$]$^+$.

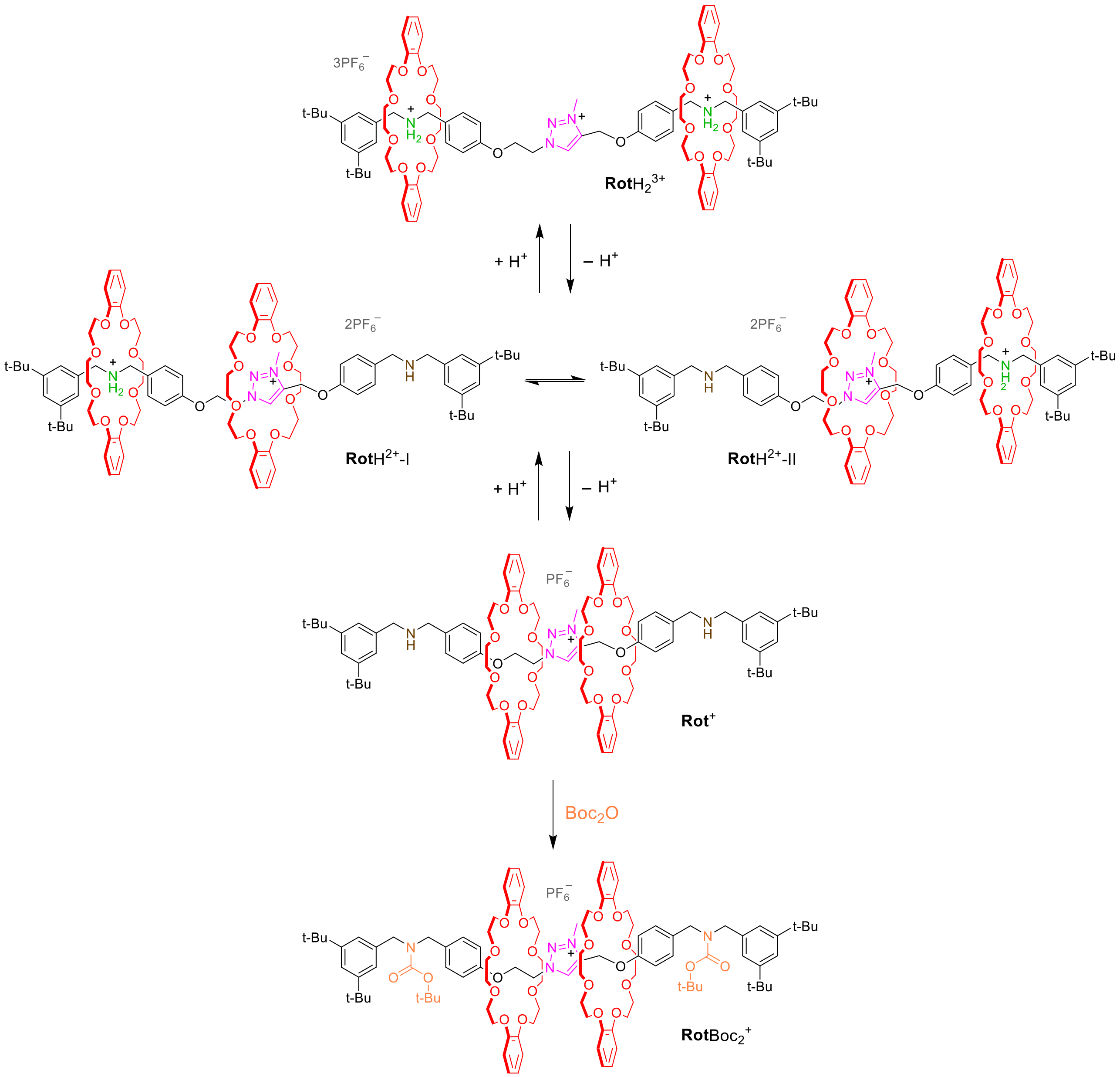

Figure S2. Reactivity of **Rot**H_2^{3+} towards deprotonation and final protection to **Rot**Boc_2^+.

General procedure for the stepwise deprotonation of compound RotH$_2^{3+}$

A solution of **Rot**H_2^{3+} (38 mg, 0.018 mmol) in methylene chloride-d_2 (500 μL) was reacted with the phosphazene base **B1** (20 mg) inside an NMR tube. The suspension was mixed for fixed time intervals and the reaction progress monitored by ^{1}H NMR spectroscopy. Upon disappearance of the peaks of **Rot**H_2^{3+}, the mixture was filtered through a pad of Celite to isolate the solution containing the dicationic compounds **Rot**H_2^+-I and **Rot**H_2^+-II. An analogous protocol, comprising the addition of the base **B1** to the solution containing the intermediates **Rot**H_2^+-I and **Rot**H_2^+-II, agitation and filtration provided the monocation **Rot**$^+$.

Monocationic [3]rotaxane Rot^{+}

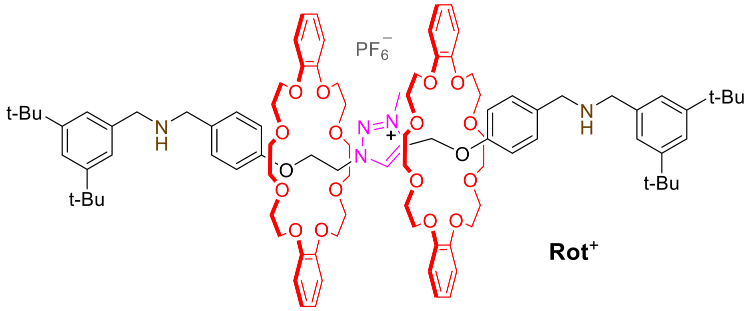

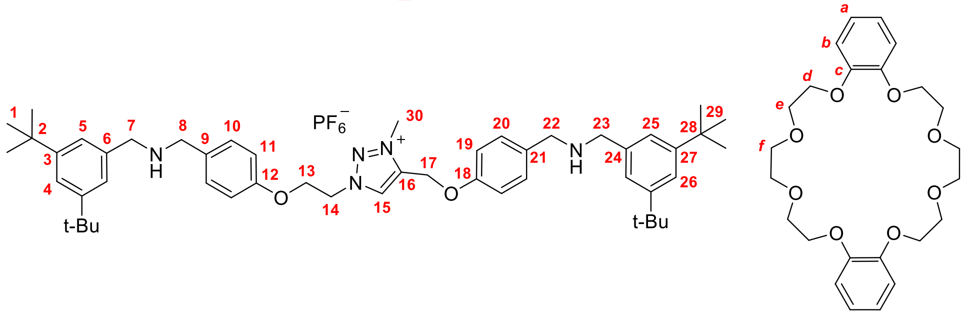

Yield: 33 mg, quantitative. ^{1}H NMR (500 MHz, Methylene Chloride-d_2) δ 8.63 (s, 1H, **15**), 7.51 (d, *J* = 8.5 Hz, 2H, **19**), 7.32 (m, 2H, **4**+**26**), 7.24 (d, *J* = 8.3 Hz, 2H, **20**), 7.16 (m, 6H, **5**+**25**+**10**), 6.88 (m, 10H, ***a***+**11**), 6.72 (m, 8H, ***b***), 5.65 (s, 2H, **17**), 5.42 (t, *J* = 8.1 Hz, 2H, **14**), 4.77 (t, *J* = 8.1 Hz, 2H, **13**), 3.98 - 3.84 (m, 16H, ***d***), 3.79 - 3.67 (m, 16H, **8**+**22**+**7**+**23**+***e***), 3.57 - 3.44 (m, 15H, **30**+***e***+***f***), 3.36 (ddt, *J* = 14.2, 5.8, 3.5 Hz, 8H, ***e***+***f***), 3.04 (tt, *J* = 6.5, 3.3 Hz, 4H, ***f***), 1.31 (d, *J* = 2.3 Hz, 36H, **1**+**29**). ^{13}C NMR (126 MHz, Methylene Chloride-d_2) δ 158.23 (**12**), 158.06 (**18**), 151.17 (**3** or **26**), 151.15 (**26** or **3**), 148.68 (***c***), 148.58 (***c***), 140.22 (**6** or **24**), 140.18 (**24** or **6**), 139.05 (**16**), 133.15 (**9**), 133.08 (**21**), 132.55 (**15**), 129.56 (**10**), 128.63 (**20**), 122.80 (**5** or **25**), 122.68 (**25** or **5**), 121.49 (**a** or **b**), 121.47 (**a** or **b**), 121.23 (**4** or **27**), 121.19 (**27** or **4**), 116.71 (**19**), 114.73 (**11**), 112.86 (**b** or **a**), 112.49 (**b** or **a**), 71.51 (***f***), 71.25 (***f***), 70.66 (***e***), 70.37 (***e***), 69.00 (***d***), 68.89 (***d***), 65.02 (**13**), 56.82 (**17**), 54.15 (**7**+**23**), 53.26 (**22**), 53.20 (**8**), 52.12 (**14**), 37.35 (**30**), 35.08 (**2**+**28**), 31.65 (**1**+**29**). ^{19}F NMR (470 MHz, Acetonitrile-d_3) δ −2.91 (d, *J* = 706.6 Hz, 6F). HRMS-ESI (*m/z*): calcd for [$C_{98}H_{135}N_5O_{18}$], 1669.9802; found 1669.9802 [**Rot**]$^{+}$.

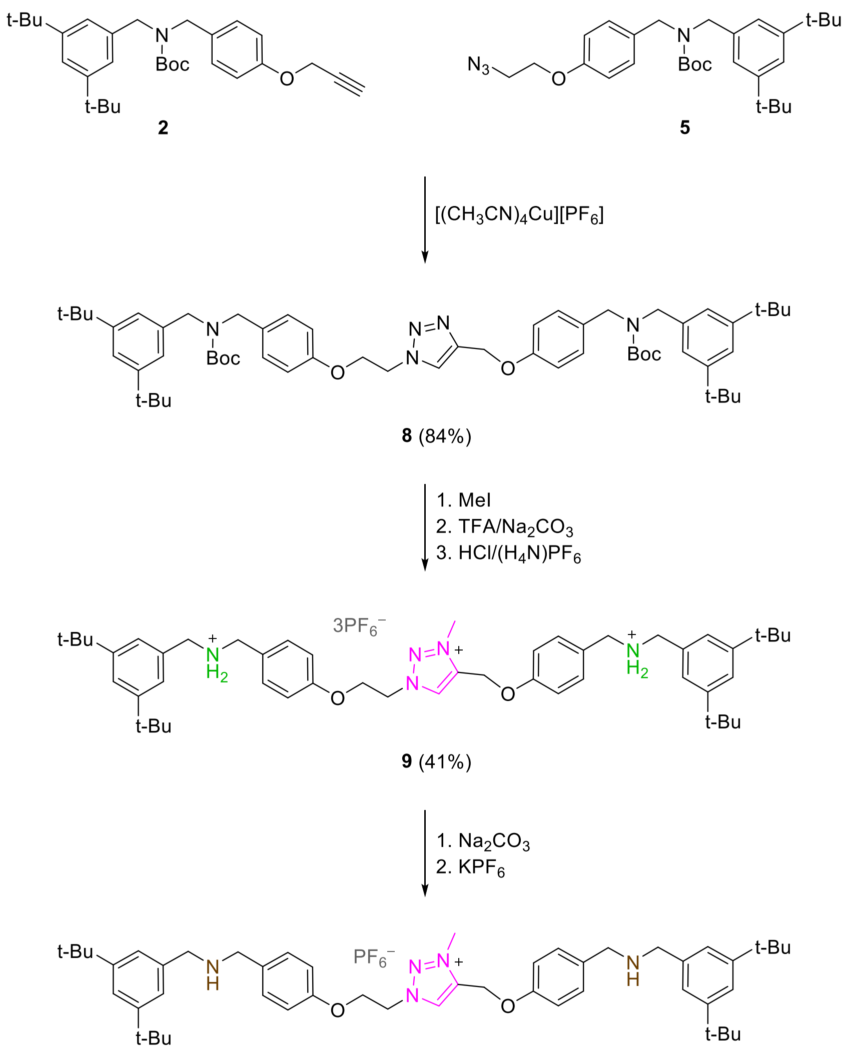

Figure S3. Synthetic route for the free axle **10**.

Protected neutral axle, 8

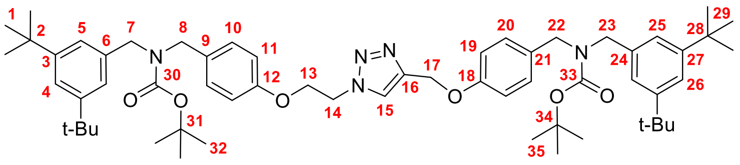

Compound **2** (110 mg, 0.30 mmol), compound **5** (113 mg, 0.30 mmol) and [Cu(MeCN)$_4$][PF$_6$] (224 mg, 0.60 mmol) were dissolved in dry dichloromethane (20 mL) and stirred at room temperature for 48 h. The mixture was then diluted with dichloromethane (50 mL) and washed with sodium ethylenediaminotetraacetate (0.1 M in H_2O, 3×25 mL), water (3×25 mL) and dried over $MgSO_4$. Filtration and removal of the solvent under reduced pressure provided a pale-yellow solid which was purified by Flash Chromatography (chloroform, R_f = 0.2) to obtain the product **8** as a colourless solid (241 mg, 84%). ^{1}H NMR (500 MHz, Methylene Chloride-*d*$_2$) δ 7.90 (s, 1H, **15**), 7.38 - 7.33 (m, 2H, **4**+**26**), 7.21 (bs, 4H, **5**+**25**), 7.08 (m, 4H, **10**+**20**), 7.00 (d, *J* = 8.5 Hz, 2H, **19**), 6.89 (d, *J* = 8.5 Hz, 2H, **11**), 5.21 (s, 2H, **17**), 4.79 (d, *J* = 5.0 Hz, 2H, **14**), 4.45 - 4.30 (m, 10H, **13**+**7**+**8**+**22**+**23**), 1.53 (d, *J* = 3.9 Hz, 18H, **32**+**35**), 1.35 (d, *J* = 6.1 Hz, 36H, **1**+**29**). ^{13}C NMR (126 MHz, Methylene Chloride-*d*$_2$) δ 158.03, 157.56, 156.19, 156.18, 151.32, 144.41, 137.91, 132.07, 131.54, 129.77,

129.67, 129.47, 124.41, 122.29, 122.26, 121.46, 115.11, 114.92, 80.01, 66.92, 62.38, 54.24, 50.52, 50.25, 50.05, 49.62, 49.06, 35.11, 35.10, 31.69, 31.68, 28.68.

Tricationic axle, 9

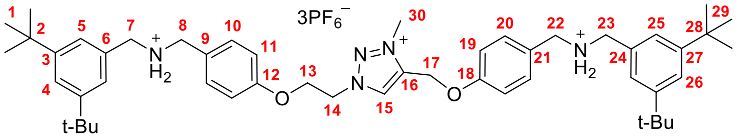

Under a dinitrogen atmosphere, compound **8** (241 mg, 0.25 mmol) was dissolved in iodomethane (15 mL) and the resulting solution was stirred for 48 h at room temperature under exclusion of light. Upon reaction completion, the solvent was removed under reduced pressure providing a yellow solid, which was dissolved in dichloromethane (30 mL) and washed with $KPF_{6(aq)}$ (3×15 mL). The organic fraction was isolated, the solvent removed under reduced pressure and the residue dissolved in trifluoroacetic acid (10 mL) and stirred at room temperature for 16 h. The acid was removed under reduced pressure and the crude product suspended in ethyl acetate (30 mL) and washed with $Na_2CO_{3(aq)}$ (3×30 mL). Removal of the solvent from the organic fraction provided a solid that was dissolved in the minimum volume of methanol (1 mL) and reacted with 37% HCl (few drops). Addition of a saturated methanol solution of $(H_4N)PF_6$ (3 mL), followed by precipitation through dropwise addition of water provided a colourless solid which was filtered and purified by sonication in diethyl ether to obtain the product **9** as a colourless solid (124 mg, 41%). ^{1}H NMR (500 MHz, Acetonitrile-d_3) δ 8.57 (s, 1H, **15**), 7.53 (m, 2H, **4**+**26**), 7.50 - 7.45 (d, *J* = 8.3 Hz, 2H, **20**), 7.44 - 7.39 (d, *J* = 8.3 Hz, 2H, **10**), 7.30 (m, 4H, **5**+**25**), 7.15 - 7.09 (d, *J* = 8.3 Hz, 2H, **19**), 7.08 - 6.81 (m, 6H, **NH$_2$**+**11**), 5.33 (s, 2H, **17**), 4.98 (t, *J* = 4.9 Hz, 2H, **14**), 4.47 (t, *J* = 4.9 Hz, 2H, **13**), 4.27 (d, *J* = 1.0 Hz, 3H, **30**), 4.22 - 4.13 (m, 8H, **7**+**8**+**22**+**23**), 1.32 (s, 36H, **1**+**29**). ^{13}C NMR (126 MHz, Acetonitrile-d_3) δ 159.66, 159.18, 152.85, 152.84, 140.49, 133.20, 133.09, 131.39, 130.75, 125.35, 125.21, 124.69, 124.67, 124.50, 116.17, 115.98, 66.08, 59.09, 54.46, 52.81, 52.75, 51.83, 51.78, 39.62, 35.65, 31.55. ^{19}F NMR (470 MHz, Acetonitrile-d_3) δ –72.89 (dd, J = 705.9, 4.9 Hz, 18F). HRMS-ESI (*m/z*): calcd for [$C_{50}H_{73}N_5O_2P_2F_{12}$], 1065.5048; found 1064.4969 [(**9**–H)$(PF_6)_2$]$^+$.

Monocationic axle, 10

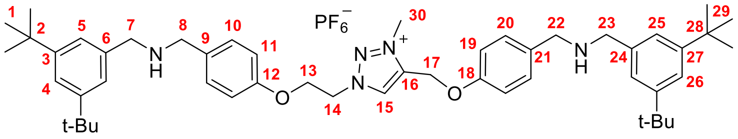

A solution of **9** (124 mg, 0.10 mmol) in dichloromethane (30 mL) was washed with $Na_2CO_{3(aq)}$ (3×30 mL) and dried over $MgSO_4$. Filtration and removal of the solvent under reduced pressure provided the product **10** as a crystalline colourless solid (90 mg, 96%). ^{1}H NMR (500 MHz, Acetonitrile-d_3) δ 8.55 (s, 1H, **15**), 7.45 - 7.41 (m, 2H, **4**+**26**), 7.36 (m, 4H, **20**+**10**), 7.24 (s, 4H, **5**+**25**), 7.04 (d, *J* = 8.7 Hz, 2H, **19**), 6.92 (d, *J* = 8.6 Hz, 2H, **11**), 5.32 (s, 2H, **17**), 4.98 - 4.92 (t, *J* = 4.9 Hz, 2H, **14**), 4.46 - 4.41 (t, *J* = 4.9 Hz, 2H, **13**), 4.26 (s, 3H, **30**), 3.94 - 3.88 (m, 8H, **7**+**8**+**22**+**23**), 1.31 (s, 36H, **1**+**29**). ^{13}C NMR (126 MHz, Acetonitrile-d_3) δ 158.66, 157.96, 152.30, 152.22, 140.71, 131.86, 131.75, 131.35, 124.38, 124.22, 123.30, 123.08, 115.93, 115.71, 66.10, 59.11, 54.51, 53.48, 53.34, 52.34, 52.32, 39.60, 35.55, 31.66, 31.64. ^{19}F NMR (470 MHz, Acetonitrile-d_3) δ –72.89 (d, *J* = 706.5 Hz, 6F). HRMS-ESI (*m/z*): calcd for [$C_{50}H_{71}N_5O_2$], 773.5608; found 772.5530 [**10**–H]$^+$.

NMR spectra

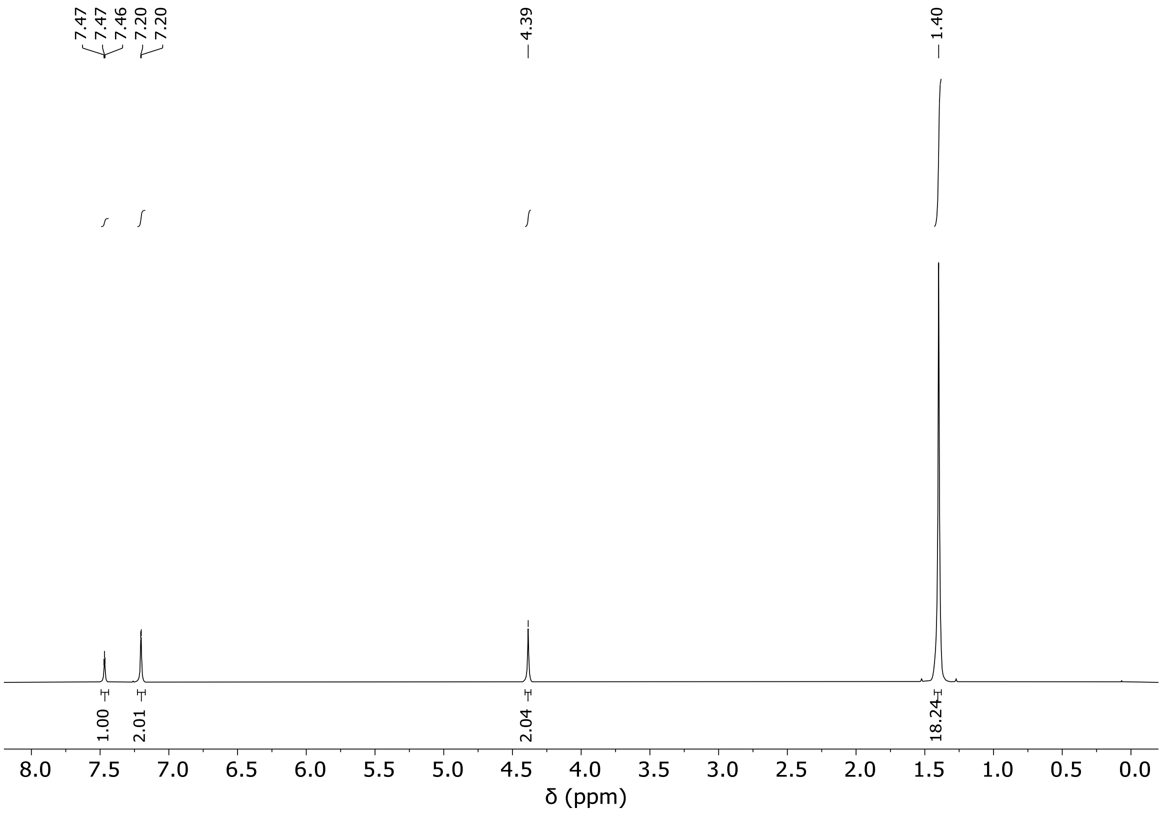

Figure S4. ^{1}H NMR spectrum of ***b*** (Chloroform-*d*, 298 K, 400 MHz).

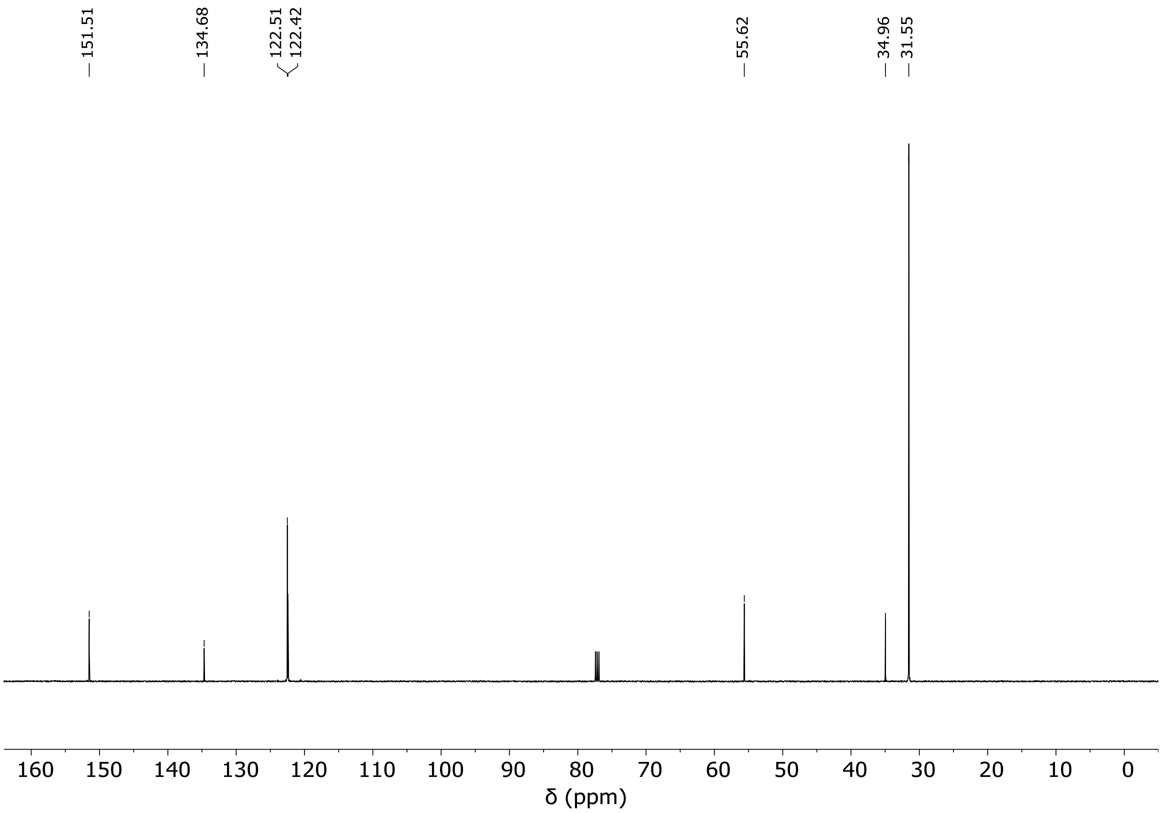

Figure S5. ^{13}C NMR spectrum of ***b*** (Chloroform-*d*, 298 K, 101 MHz).

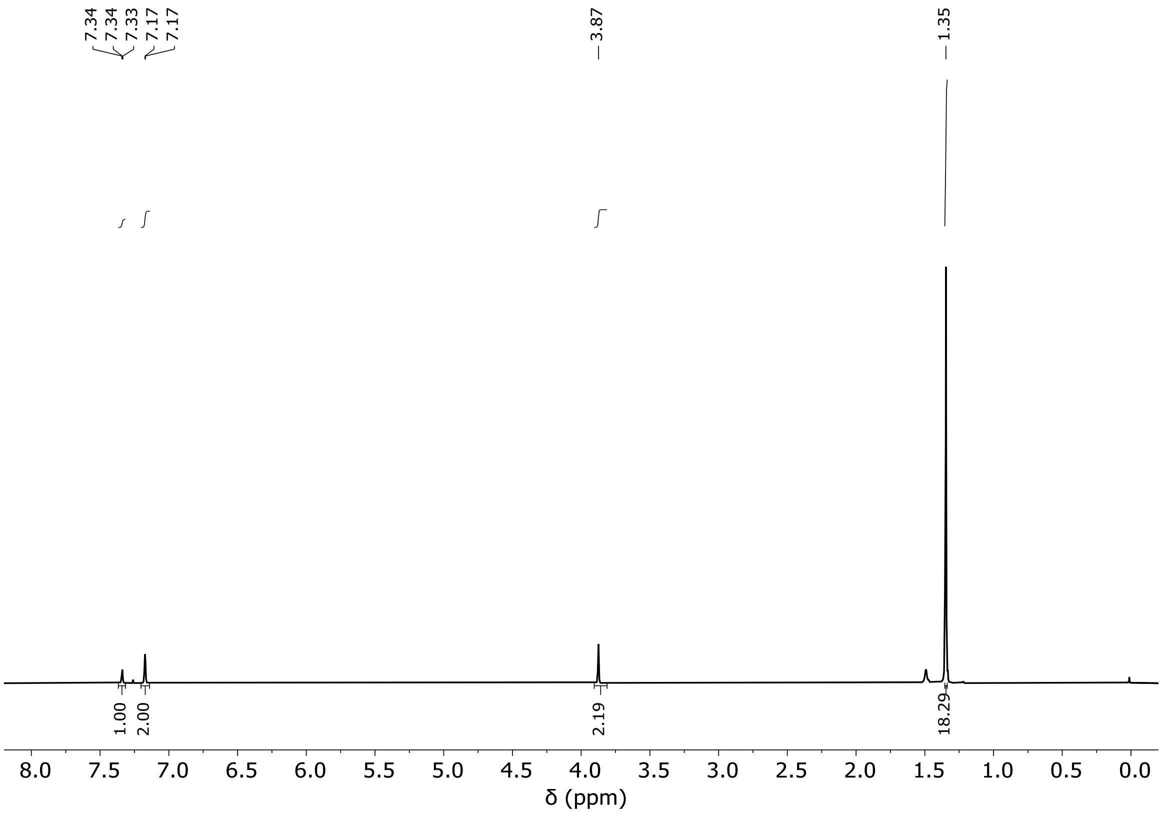

Figure S6. ^{1}H NMR spectrum of ***c*** (Chloroform-*d*, 298 K, 400 MHz).

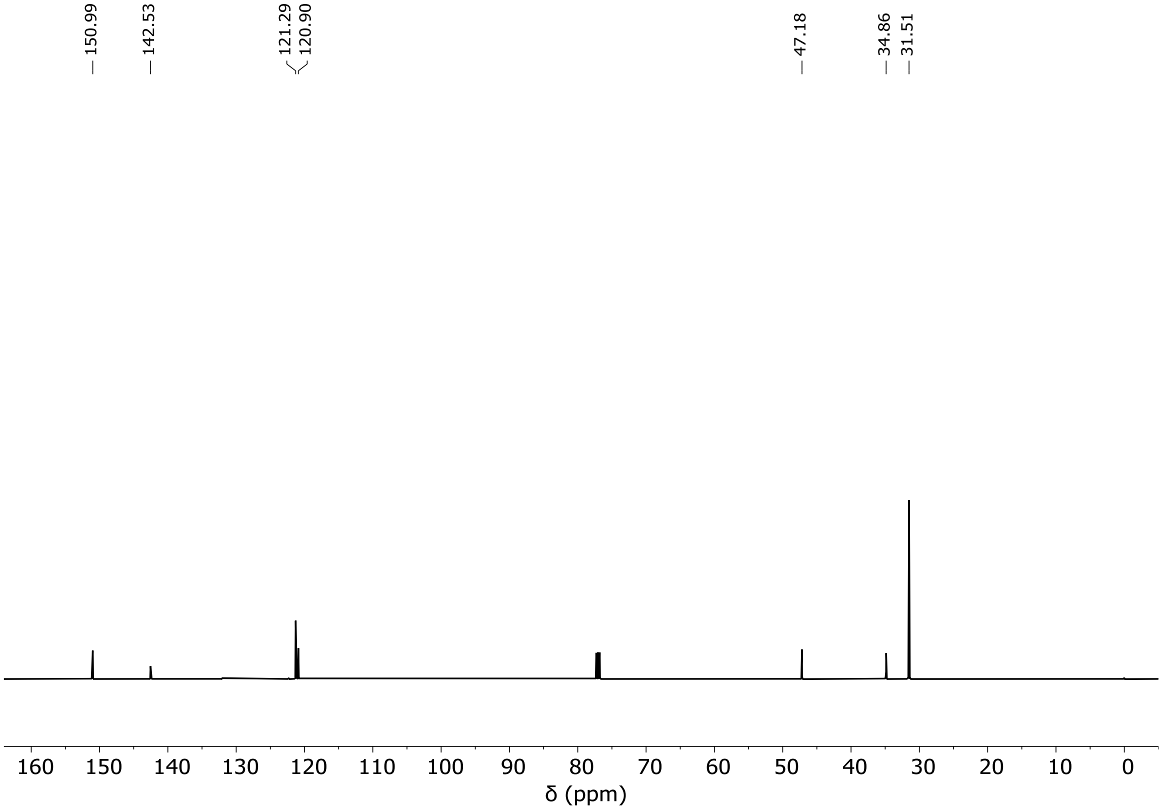

Figure S7. ^{13}C NMR spectrum of ***c*** (Chloroform-*d*, 298 K, 101 MHz).

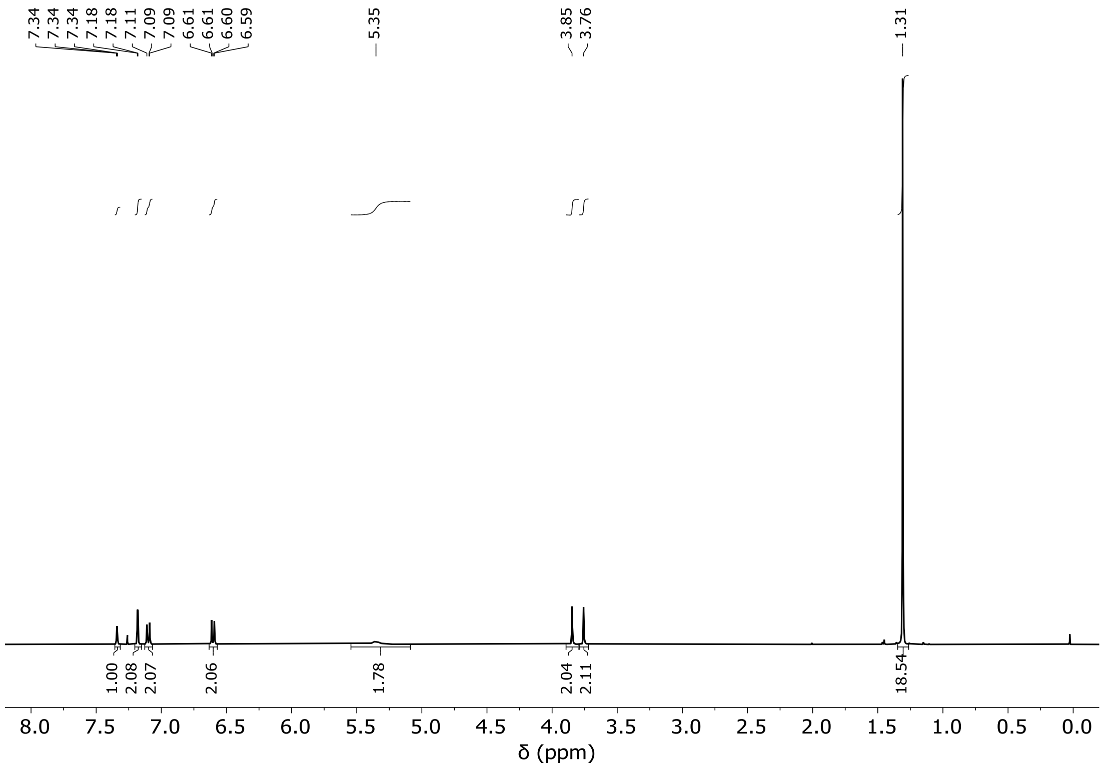

Figure S8. ^{1}H NMR spectrum of ***d*** (Chloroform-*d*, 298 K, 400 MHz).

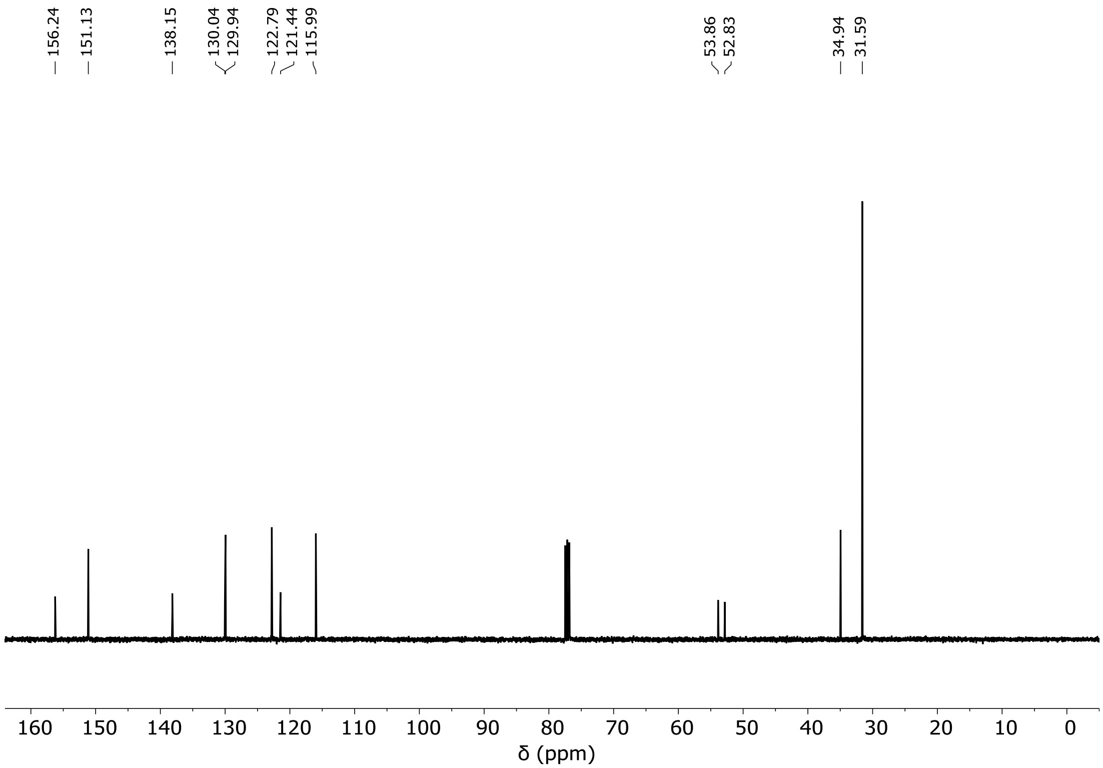

Figure S9. ^{13}C NMR spectrum of ***d*** (Chloroform-*d*, 298 K, 101 MHz).

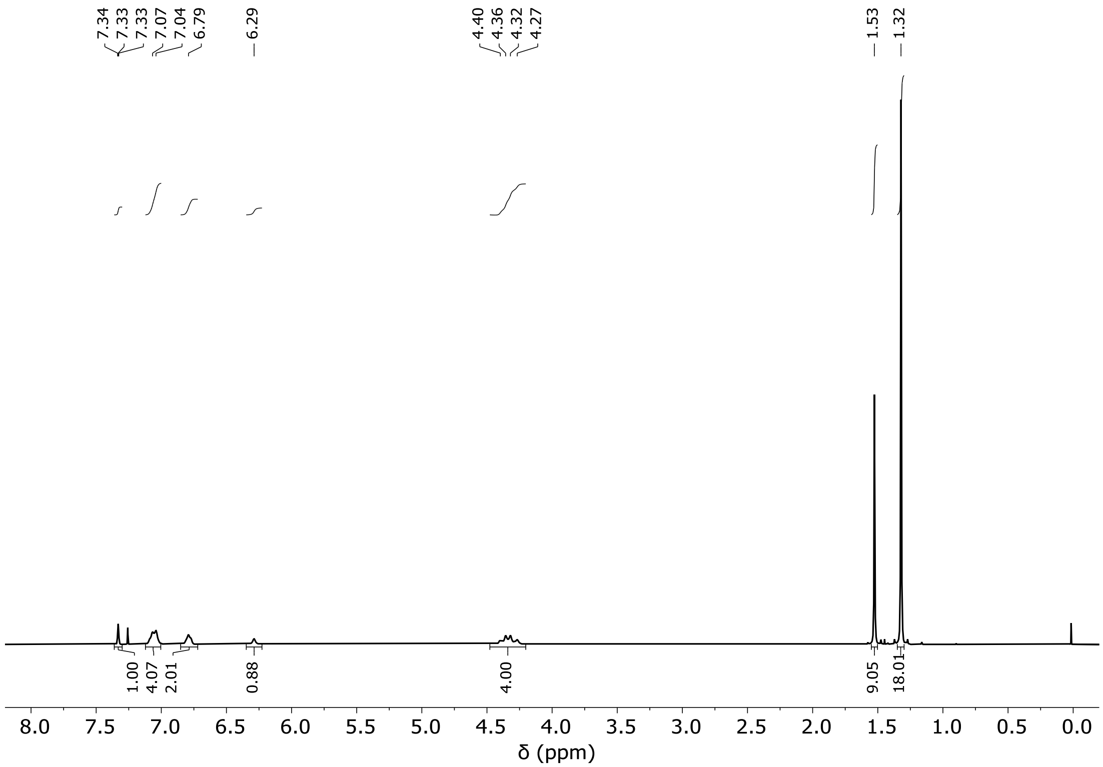

Figure S10. ^{1}H NMR spectrum of **1** (Chloroform-*d*, 298 K, 400 MHz).

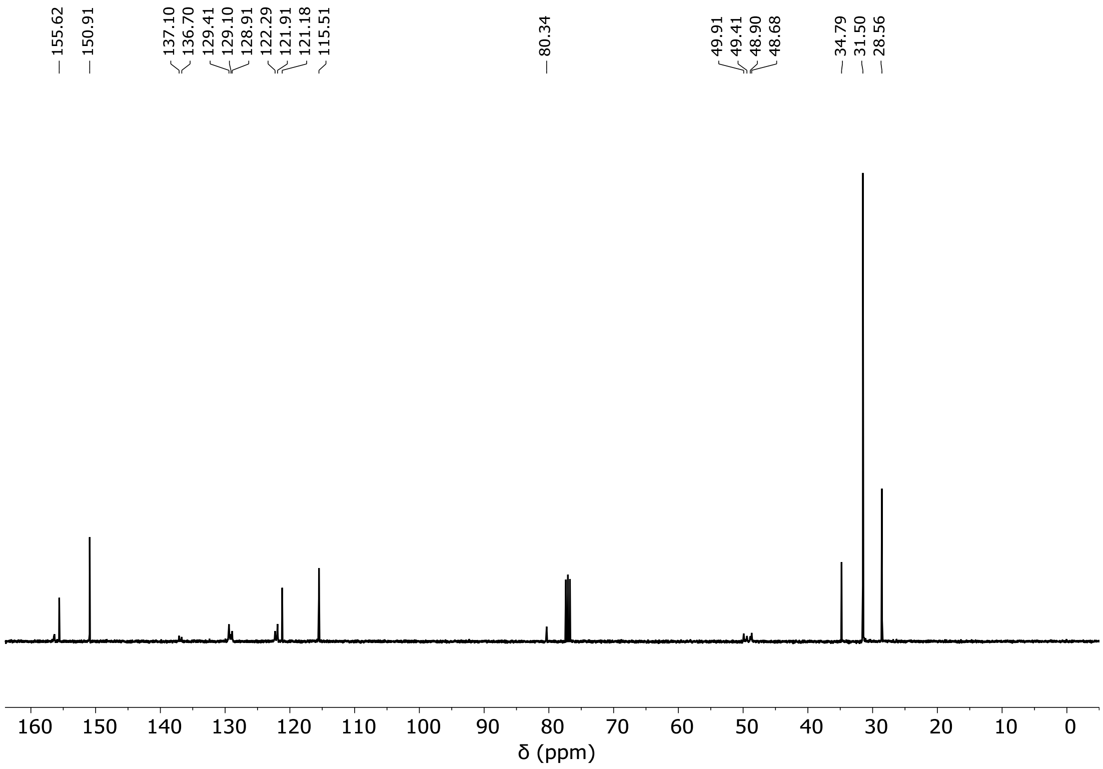

Figure S11. ^{13}C NMR spectrum of **1** (Chloroform-*d*, 298 K, 101 MHz).

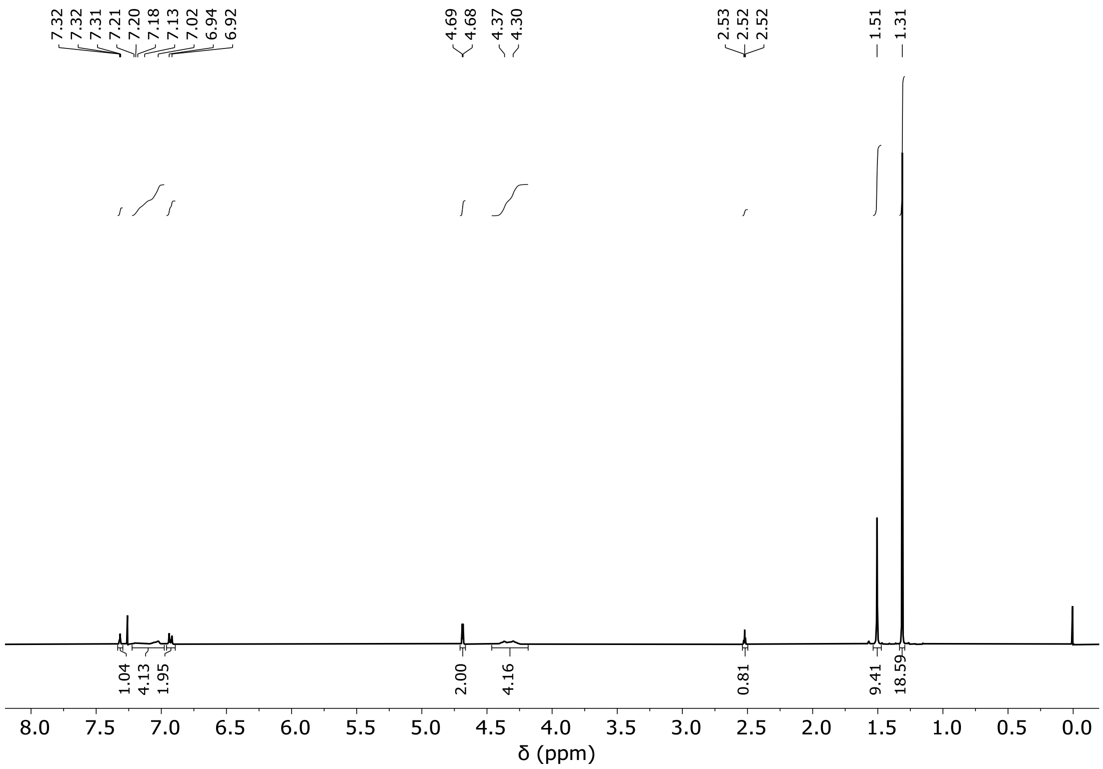

Figure S12. ^{1}H NMR spectrum of **2** (Chloroform-*d*, 298 K, 400 MHz).

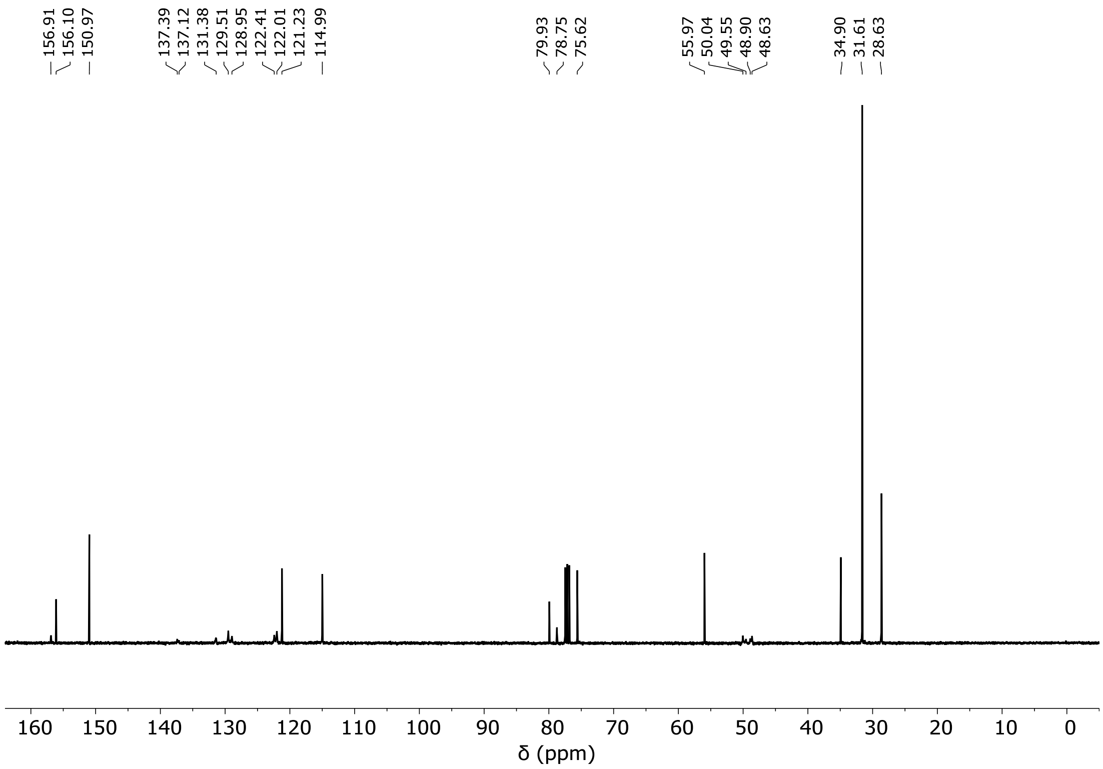

Figure S13. ^{13}C NMR spectrum of **2** (Chloroform-*d*, 298 K, 101 MHz).

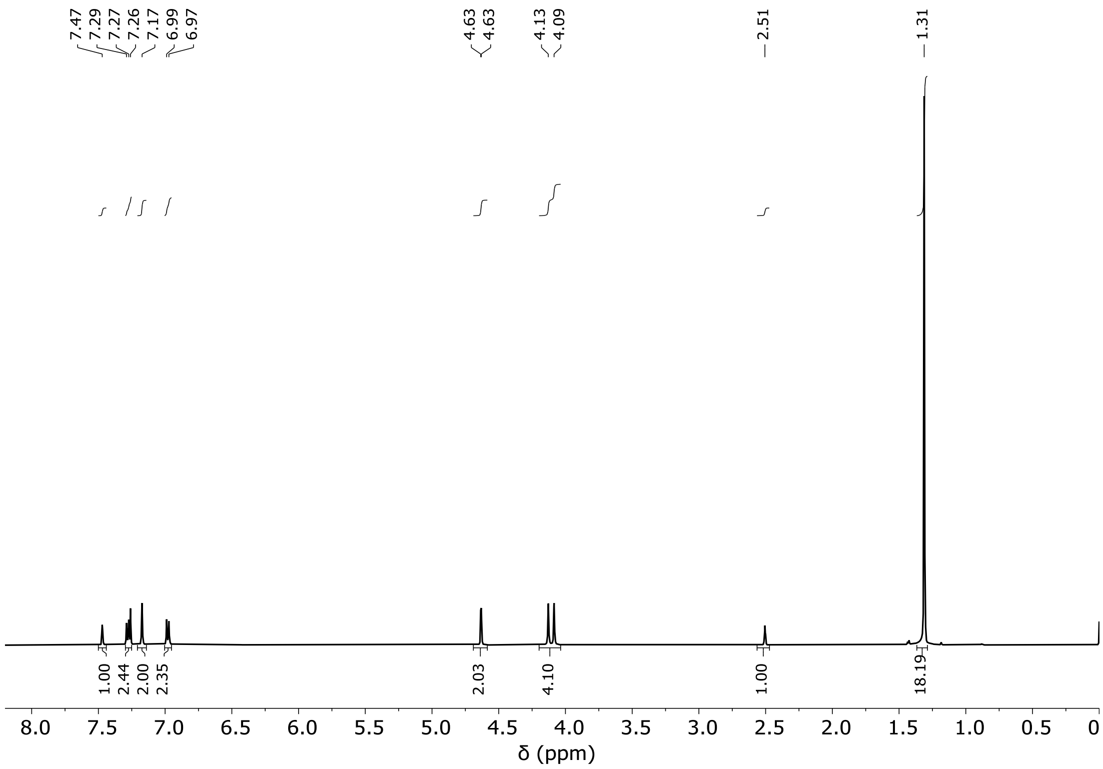

Figure S14. ^{1}H NMR spectrum of **3** (Chloroform-*d*, 298 K, 400 MHz).

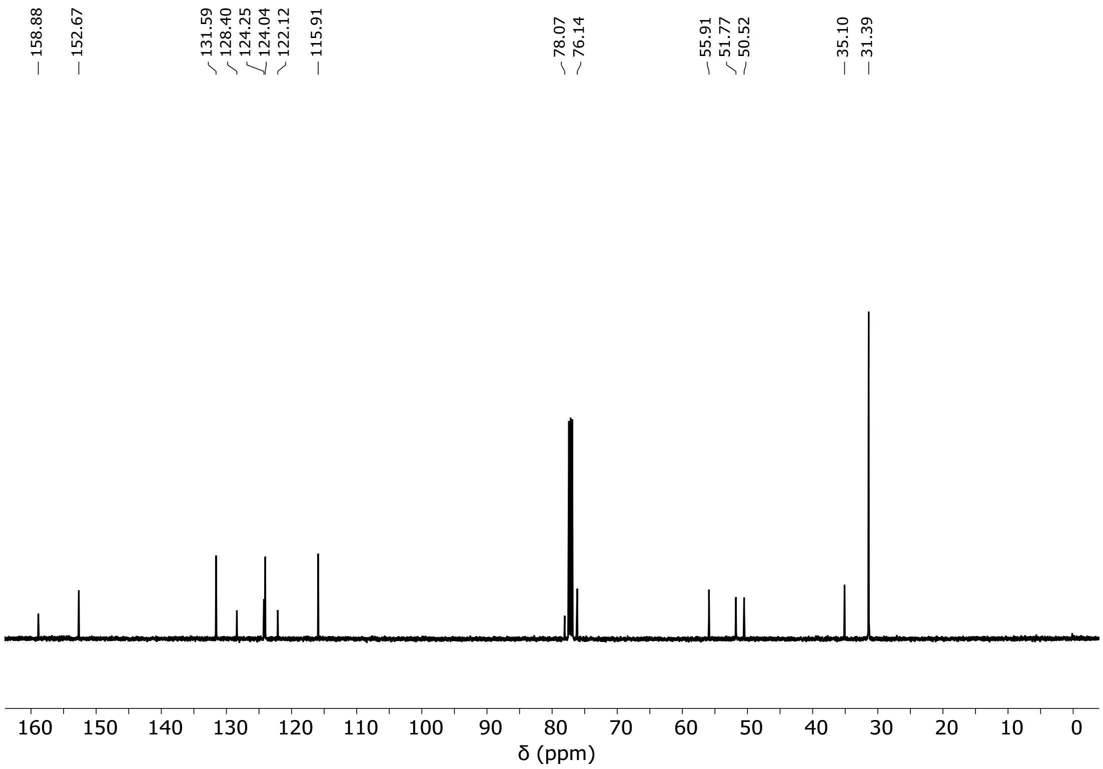

Figure S15. ^{13}C NMR spectrum of **3** (Chloroform-*d*, 298 K, 126 MHz).

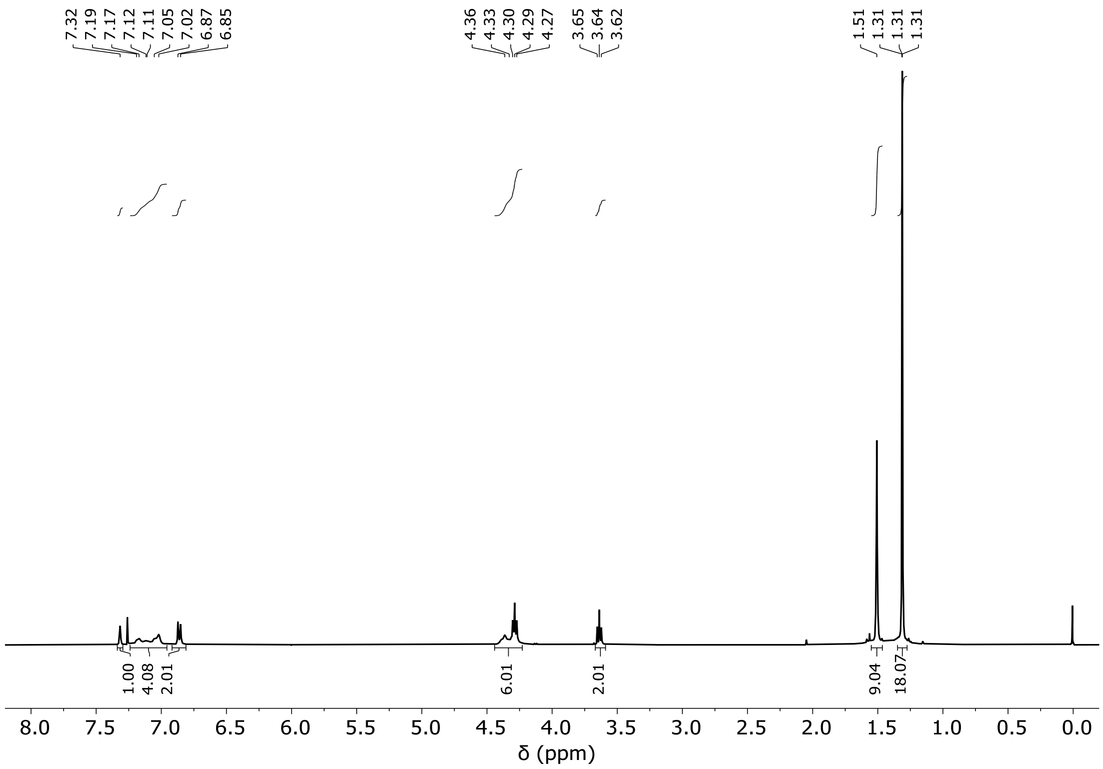

Figure S16. ^{1}H NMR spectrum of **4** (Chloroform-*d*, 298 K, 400 MHz).

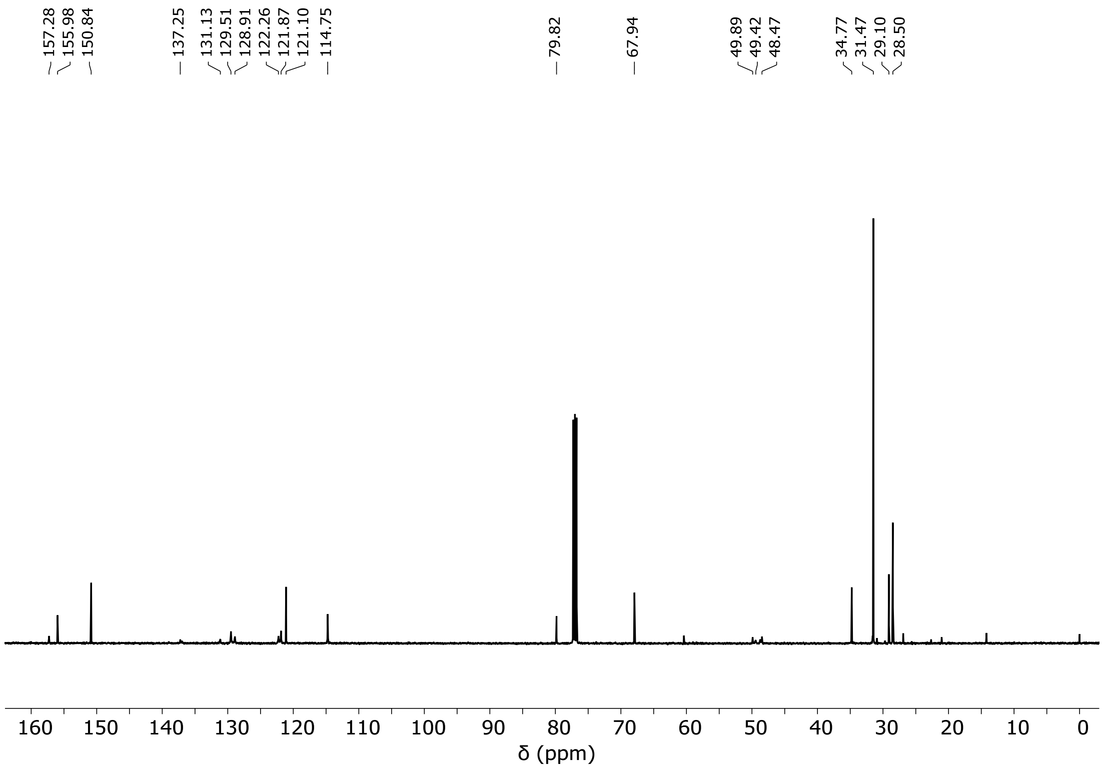

Figure S17. ^{13}C NMR spectrum of **4** (Chloroform-*d*, 298 K, 126 MHz).

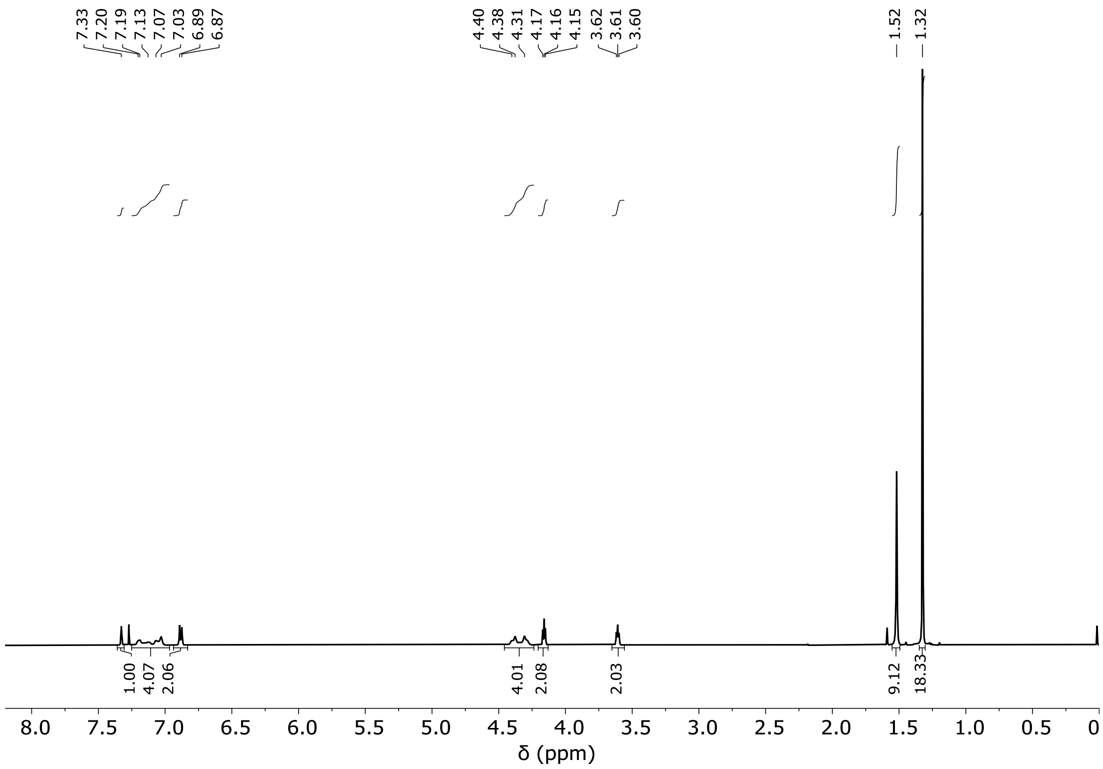

Figure S18. ^{1}H NMR spectrum of **5** (Chloroform-*d*, 298 K, 400 MHz).

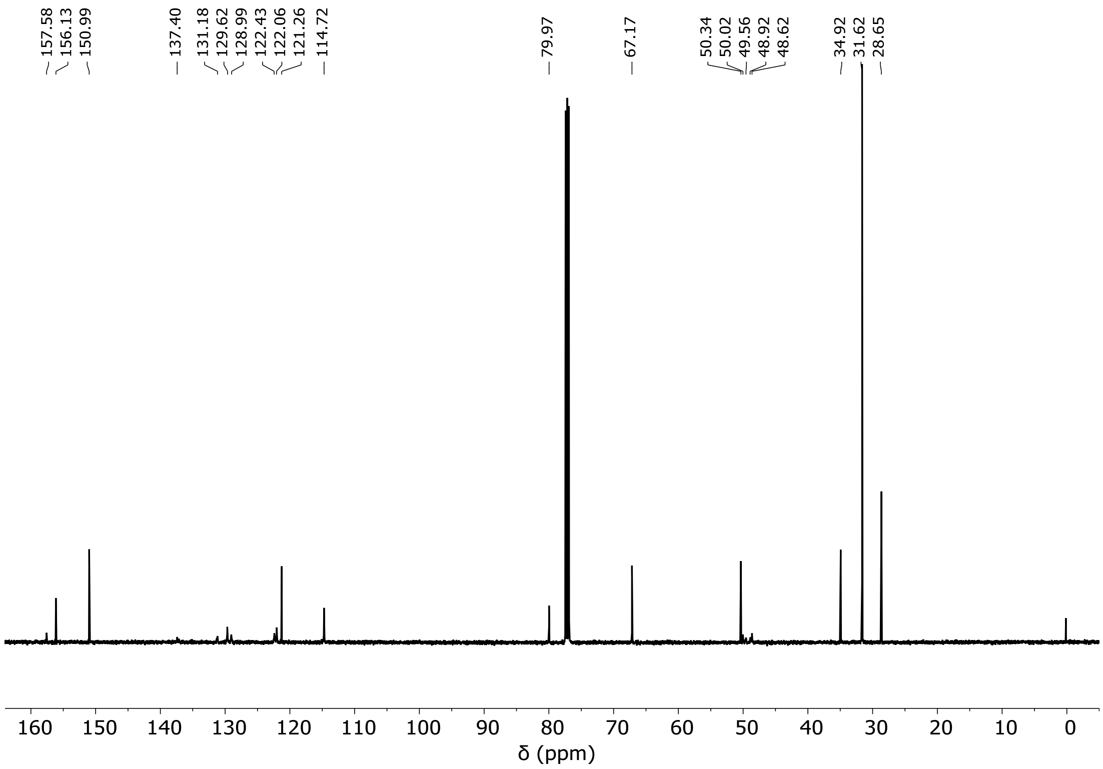

Figure S19. ^{13}C NMR spectrum of **5** (Chloroform-*d*, 298 K, 101 MHz).

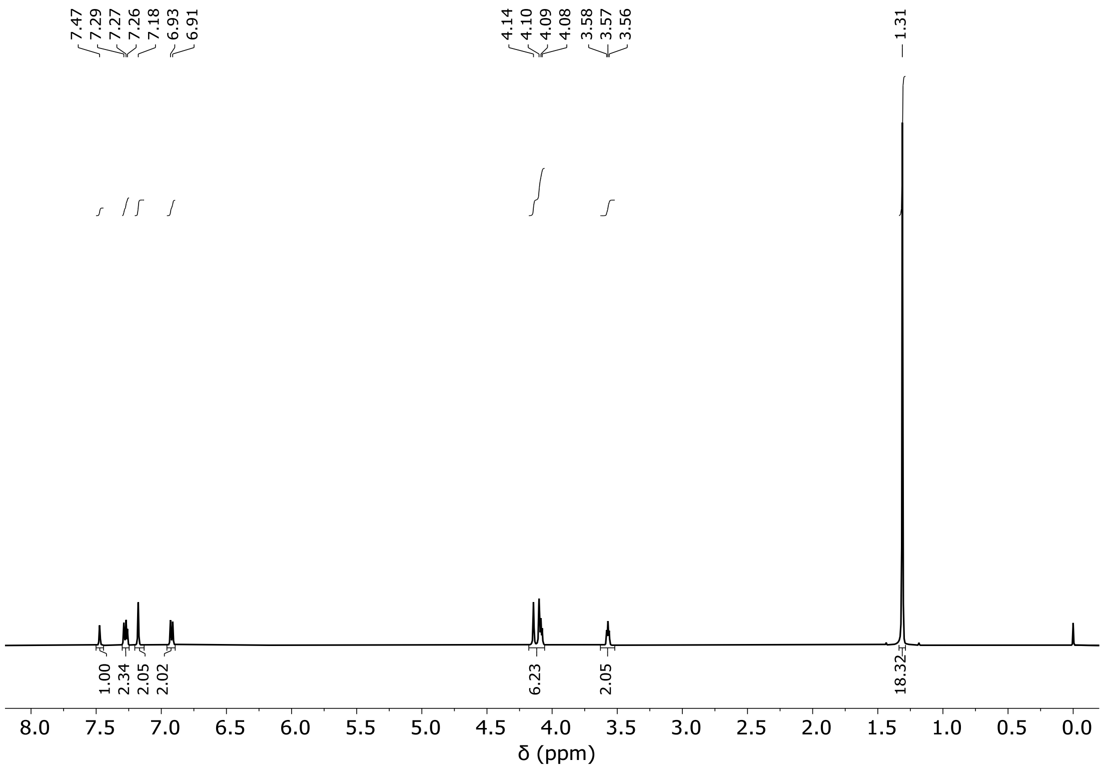

Figure S20. ^{1}H NMR spectrum of **6** (Chloroform-*d*, 298 K, 400 MHz).

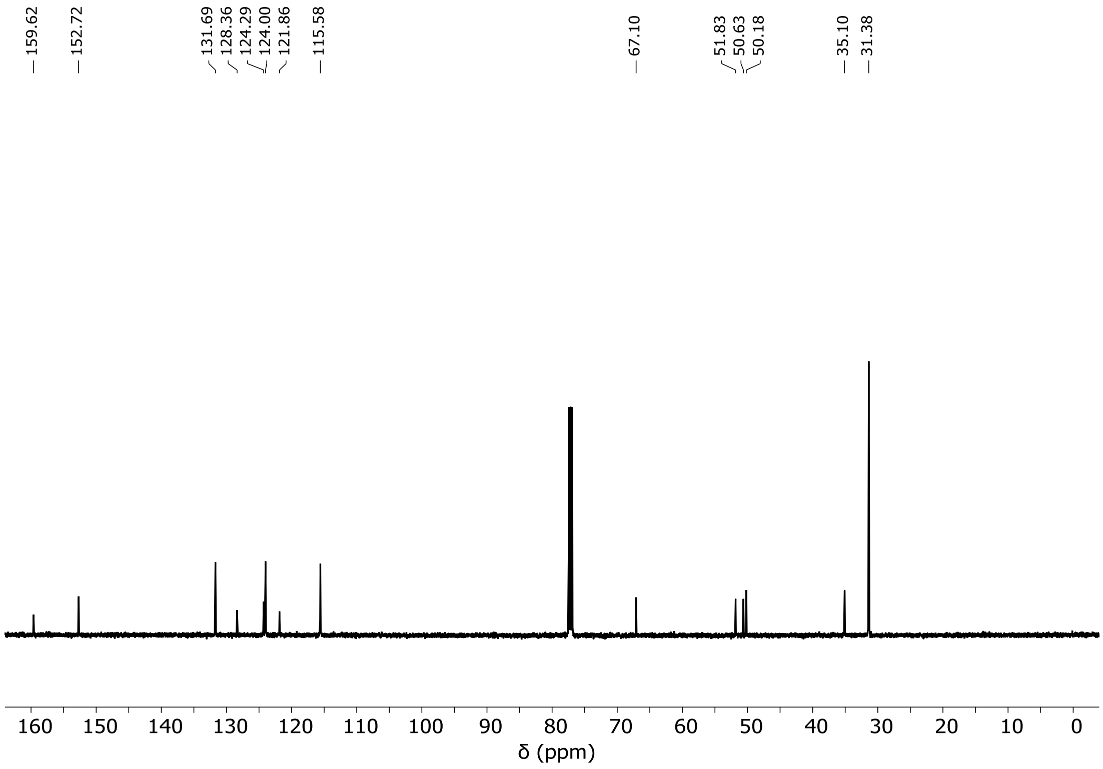

Figure S21. ^{13}C NMR spectrum of **6** (Chloroform-*d*, 298 K, 126 MHz).

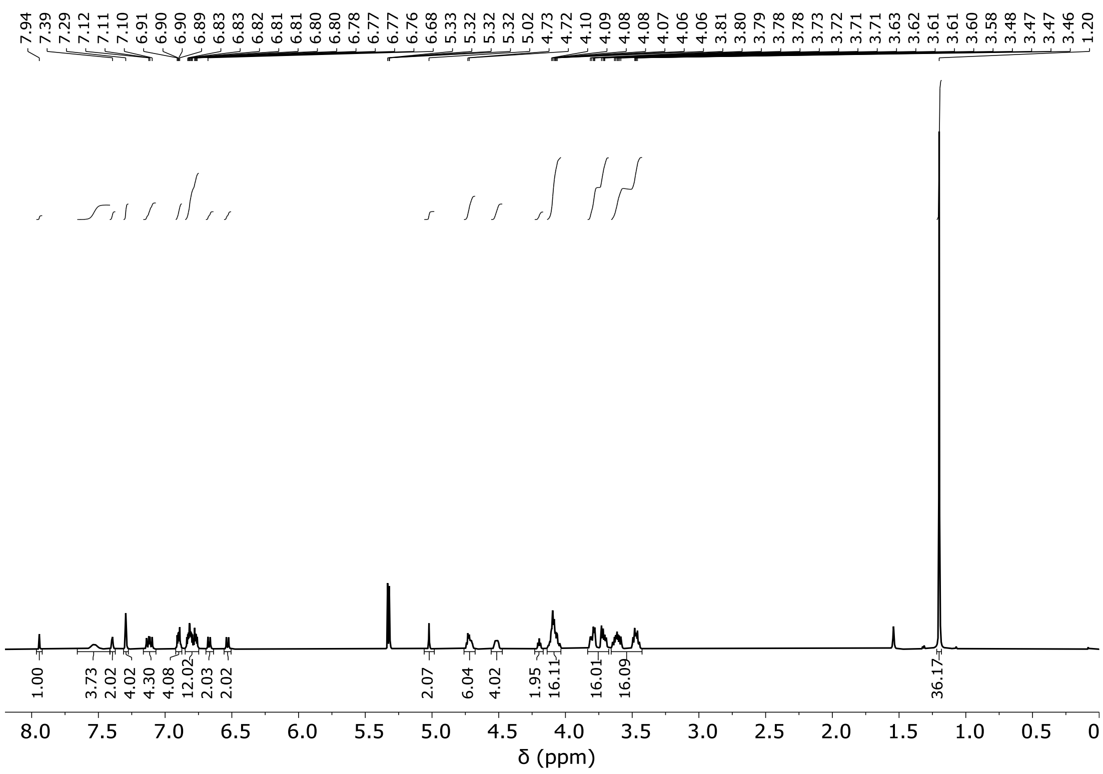

Figure S22. ^{1}H NMR spectrum of **7** (Methylene Chloride-d_2, 298 K, 500 MHz).

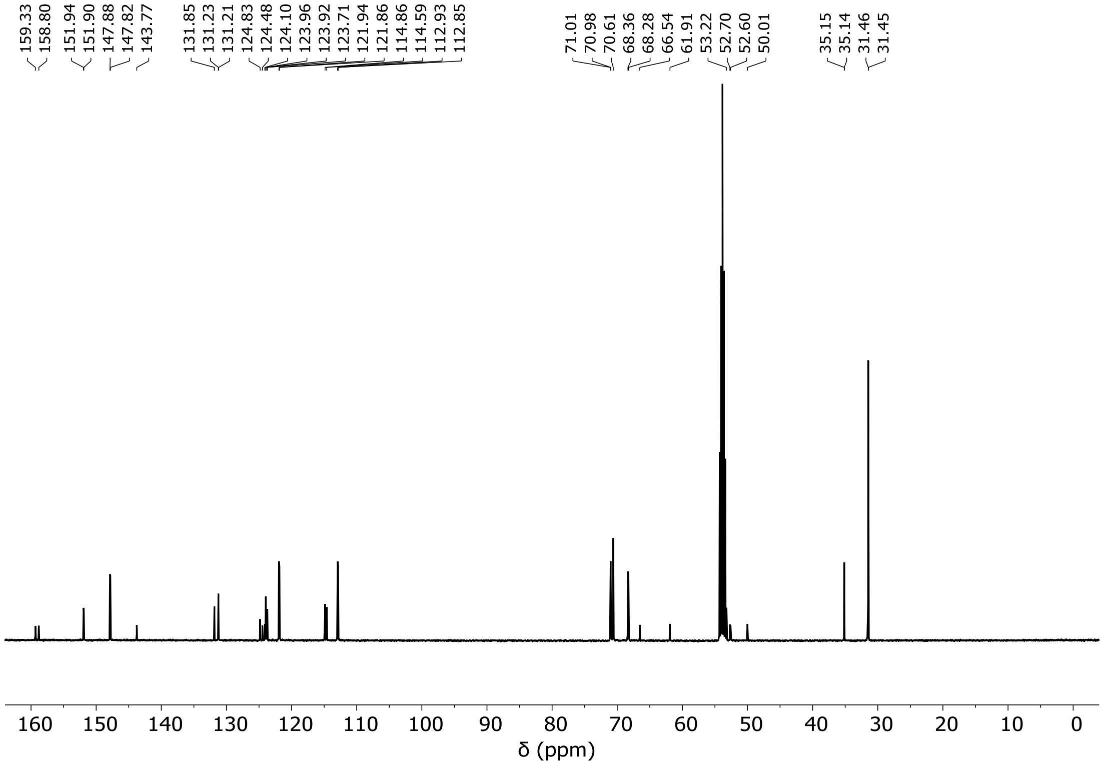

Figure S23. ^{13}C NMR spectrum of **7** (Methylene Chloride-d_2, 298 K, 126 MHz).

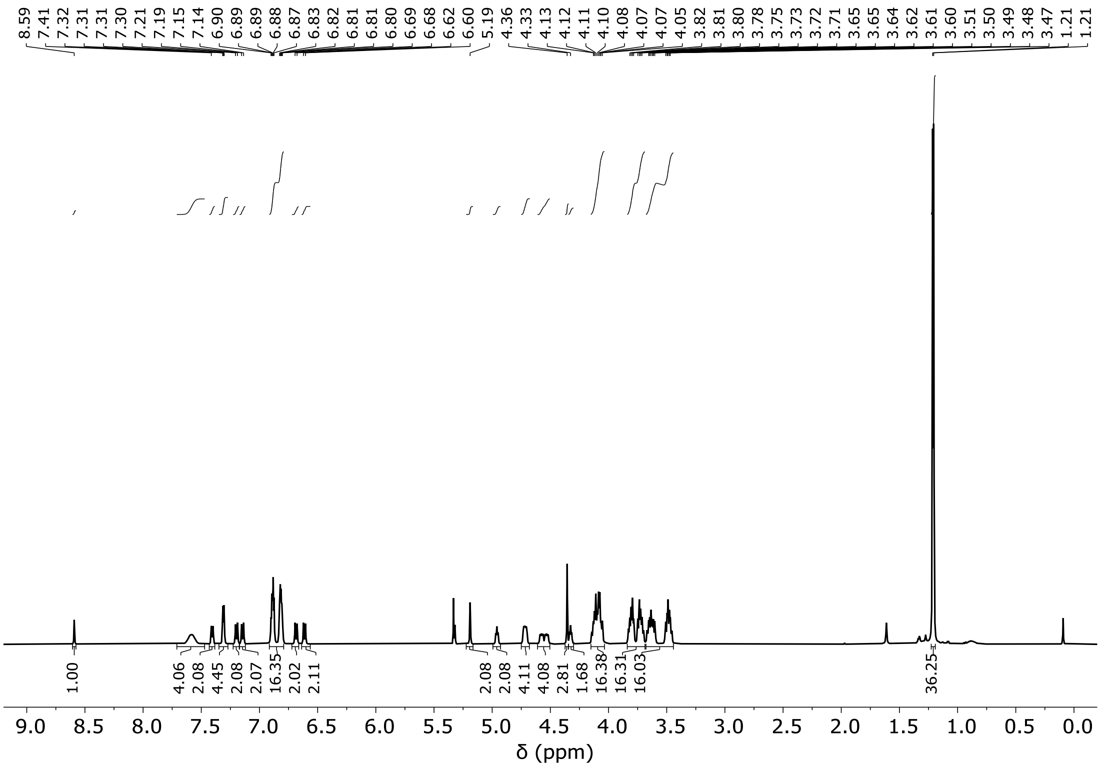

Figure S24. ^{1}H NMR spectrum of **Rot**H_2^{3+} (Methylene Chloride-d_2, 298 K, 500 MHz).

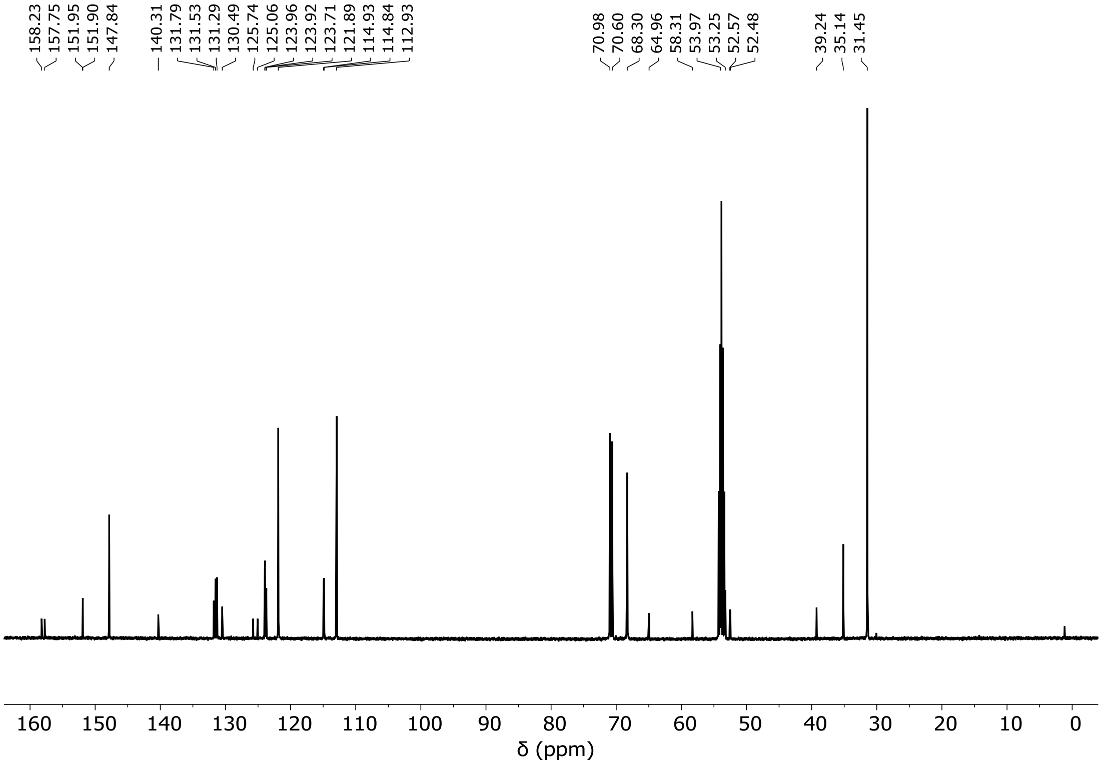

Figure S25. ^{13}C NMR spectrum of **Rot**H_2^{3+} (Methylene Chloride-d_2, 298 K, 126 MHz).

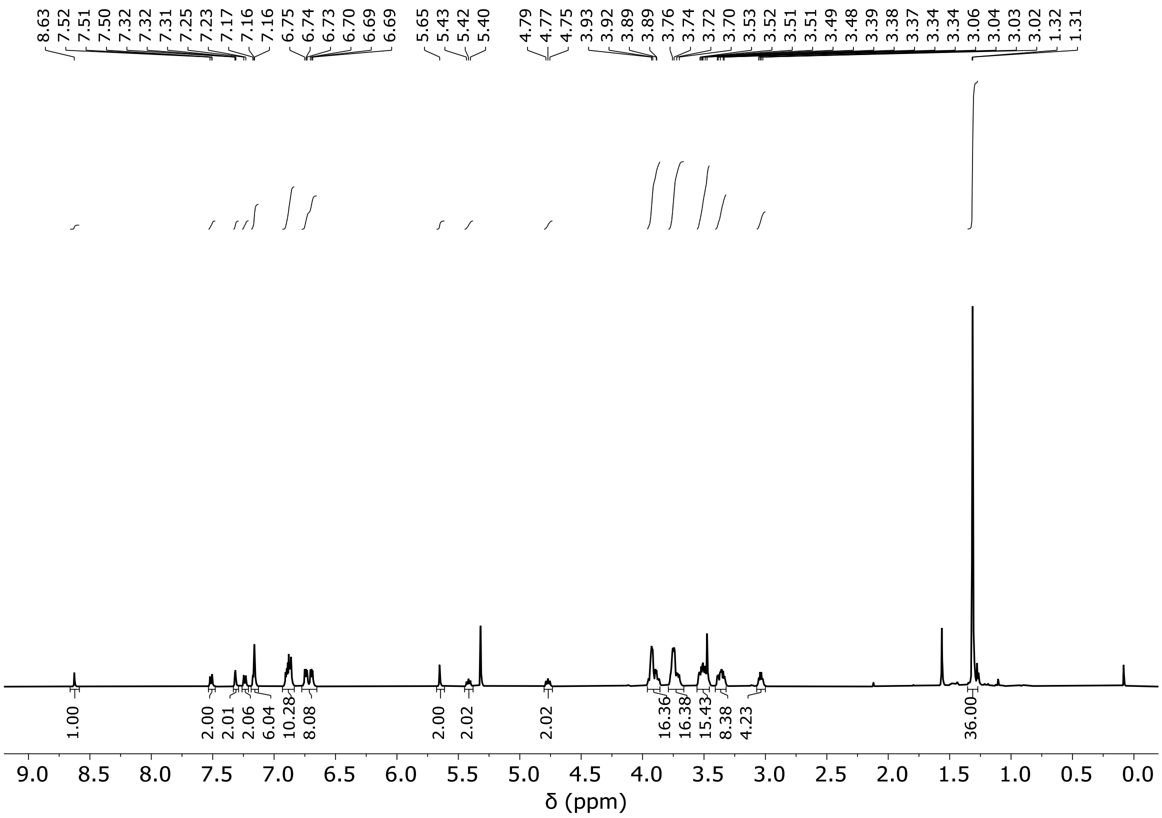

Figure S26. ^{1}H NMR spectrum of **Rot**$^{+}$ (Methylene Chloride-d_2, 298 K, 500 MHz).

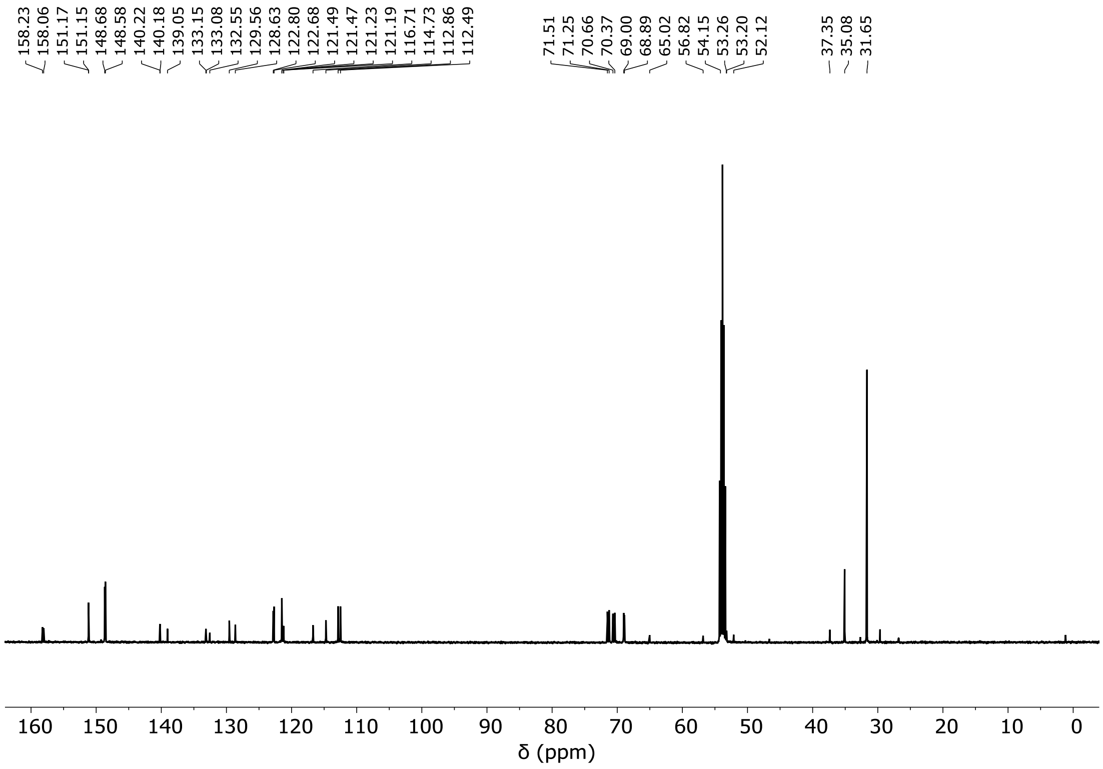

Figure S27. ^{13}C NMR spectrum of **Rot**$^{+}$ (Methylene Chloride-d_2, 298 K, 126 MHz).

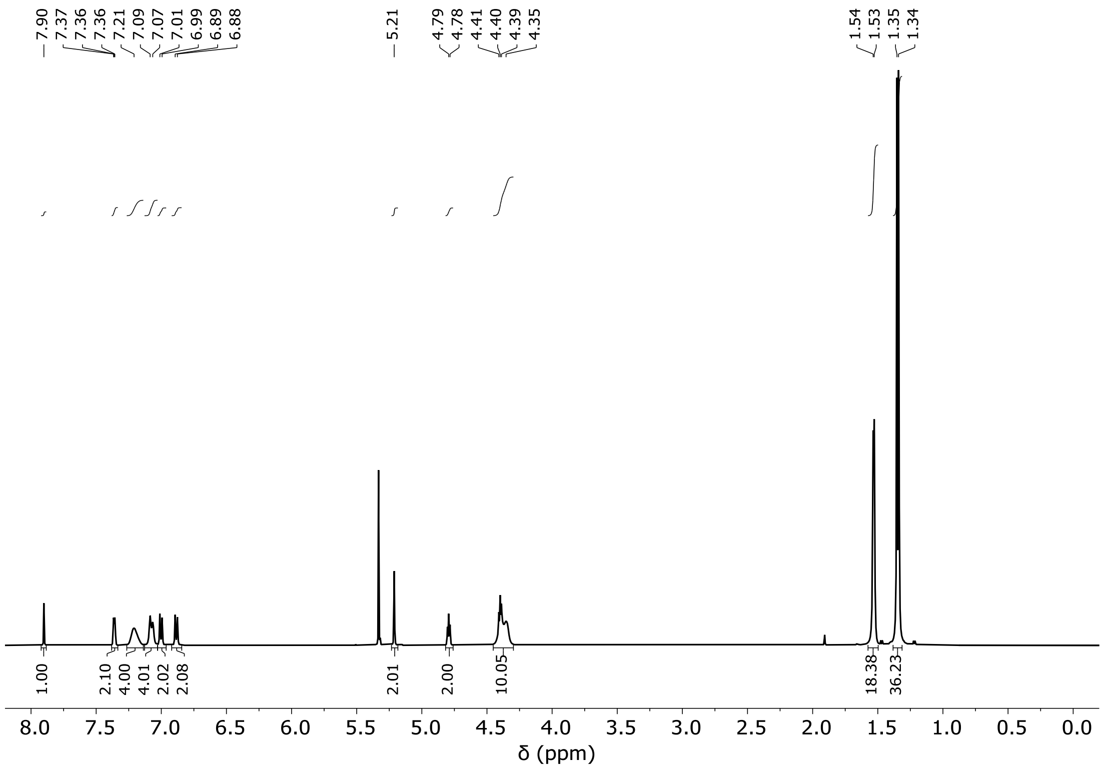

Figure S28. ^{1}H NMR spectrum of **8** (Methylene Chloride-d_2, 298 K, 500 MHz).

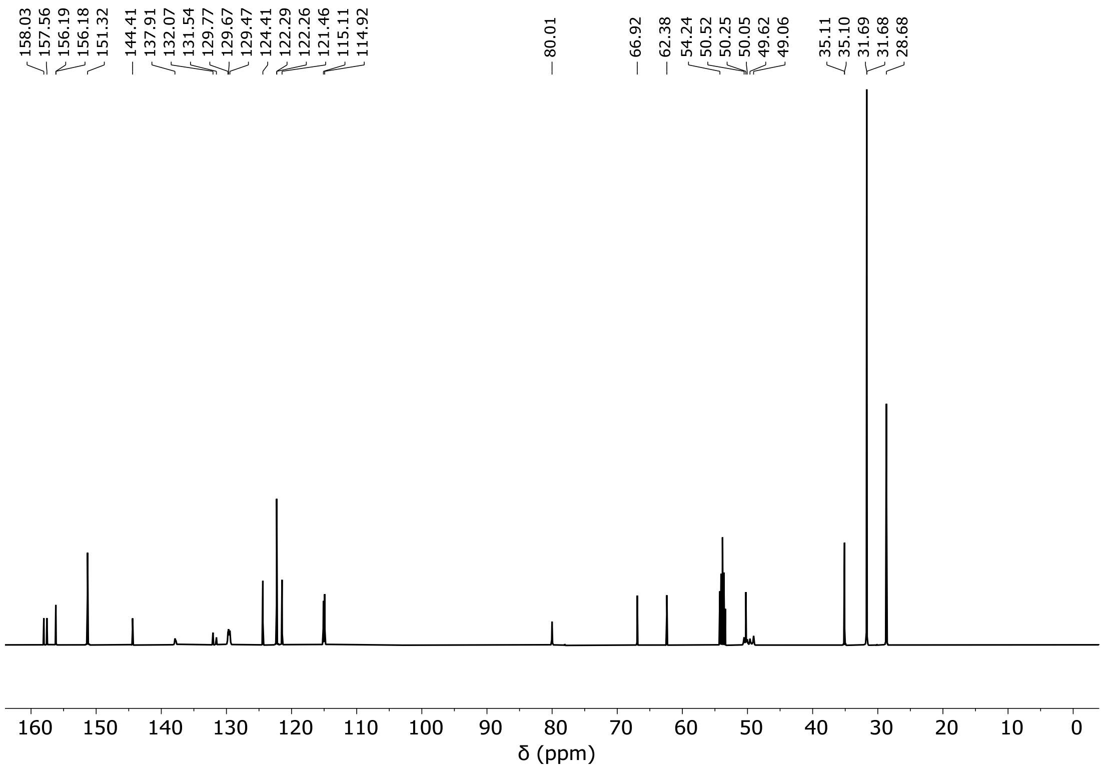

Figure S29. ^{13}C NMR spectrum of **8** (Methylene Chloride-d_2, 298 K, 126 MHz).

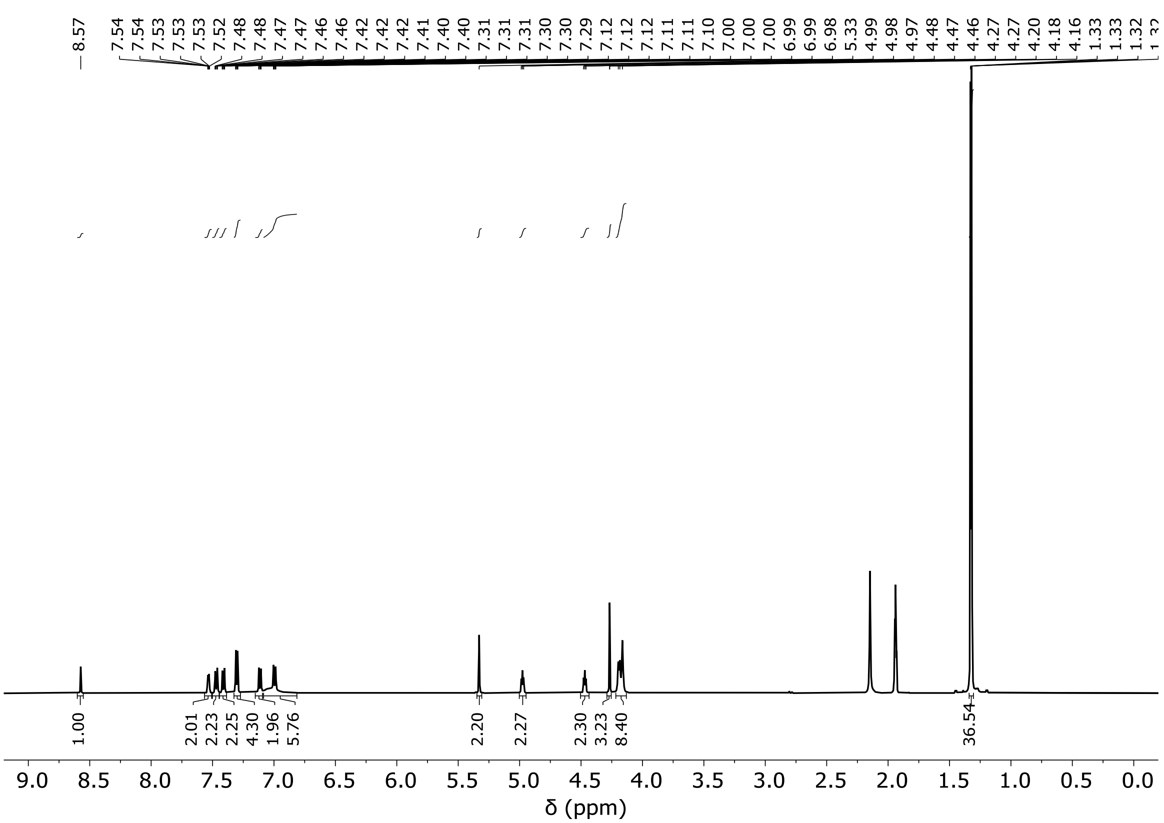

Figure S30. ^{1}H NMR spectrum of **9** (Acetonitrile-d_3, 298 K, 400 MHz).

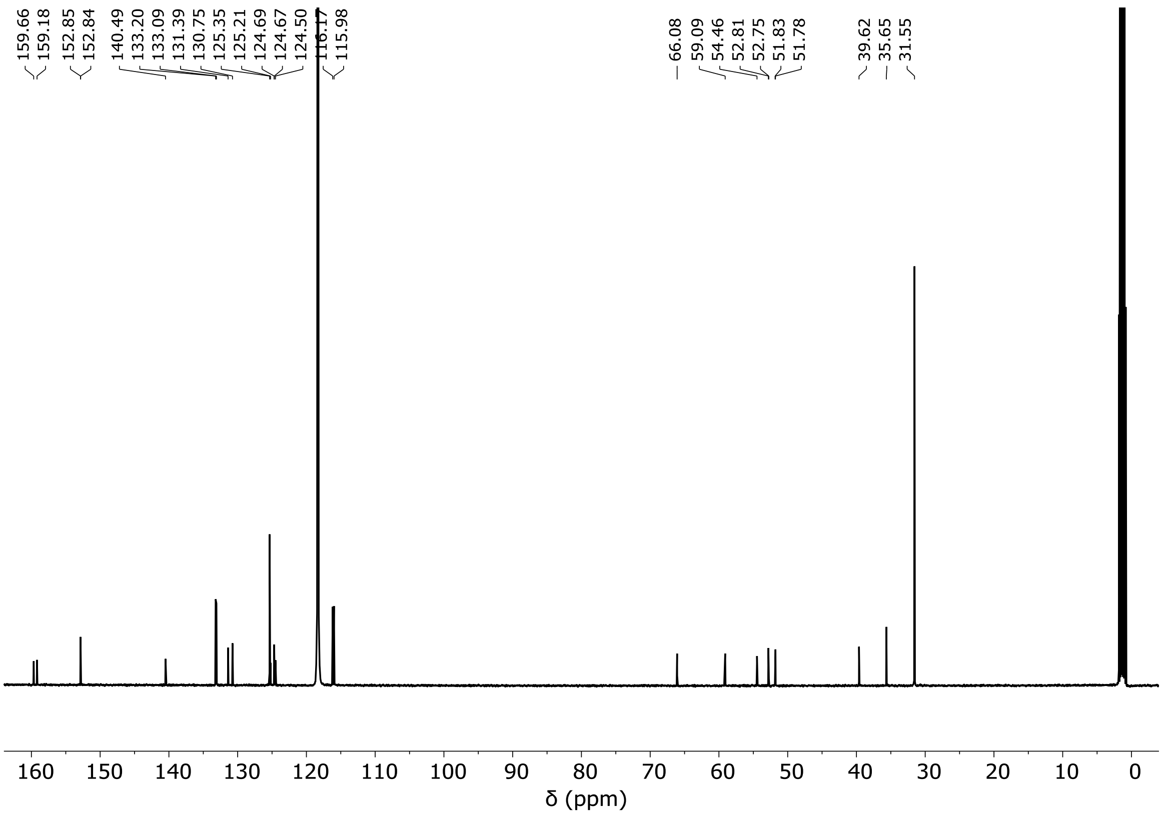

Figure S31. ^{13}C NMR spectrum of **9** (Acetonitrile-d_3, 298 K, 126 MHz).

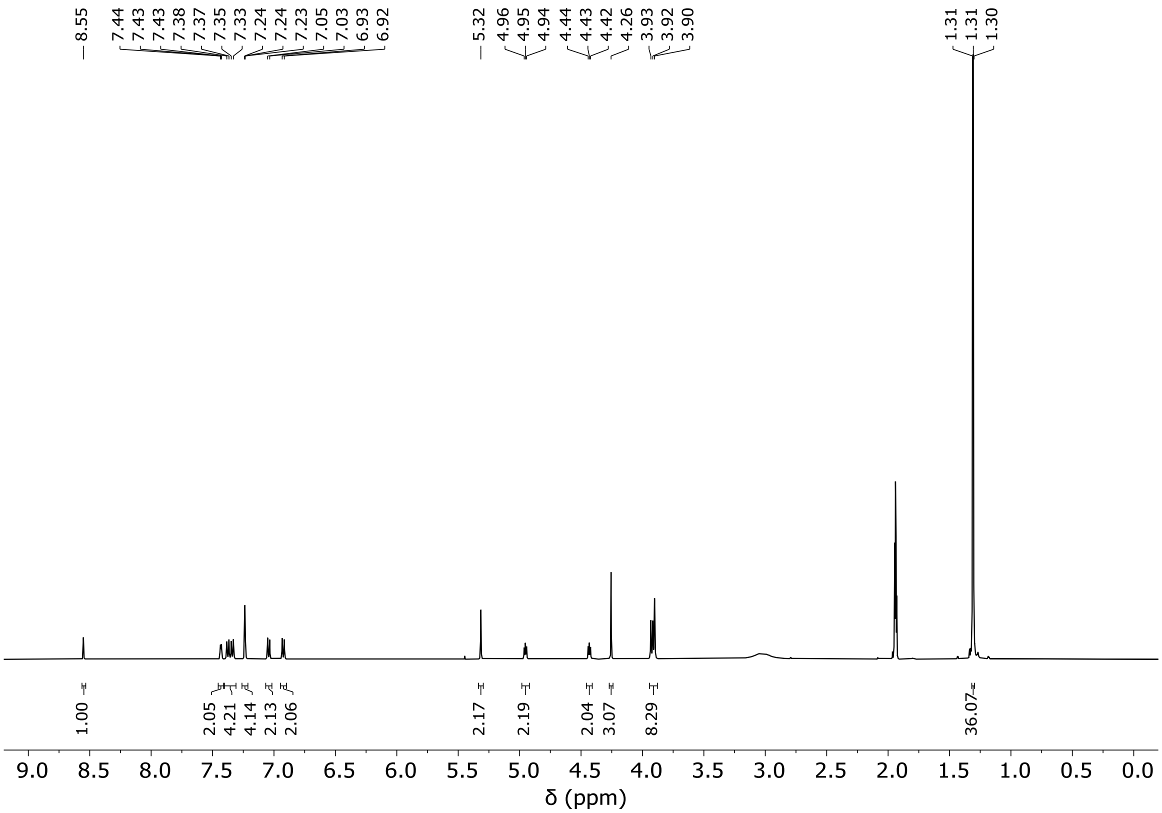

Figure S32. ^{1}H NMR spectrum of **10** (Acetonitrile-d_3, 298 K, 400 MHz).

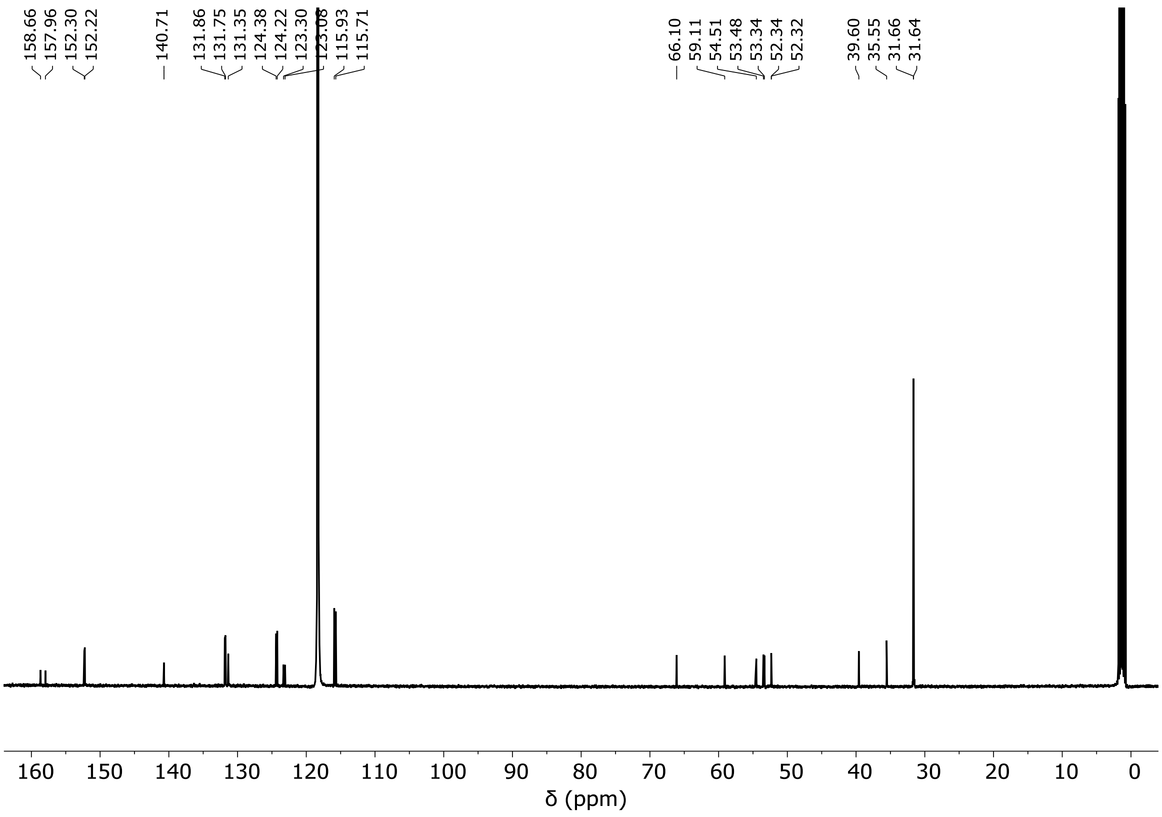

Figure S33. ^{13}C NMR spectrum of **10** (Acetonitrile-d_3, 298 K, 126 MHz).

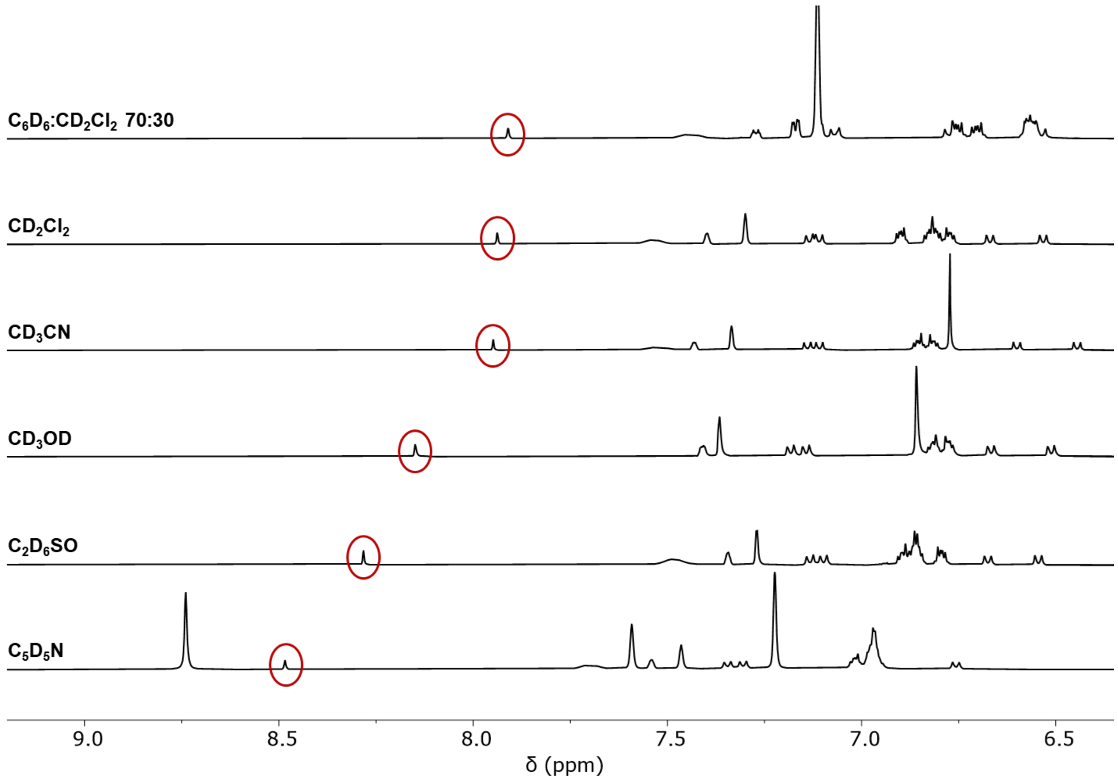

Figure S34. Portion of the ^{1}H NMR spectra of **7** in different solvents (298 K, 500 MHz). The peaks circled in red in the region between 7.8 ppm and 8.6 ppm are related to the triazole aromatic proton.

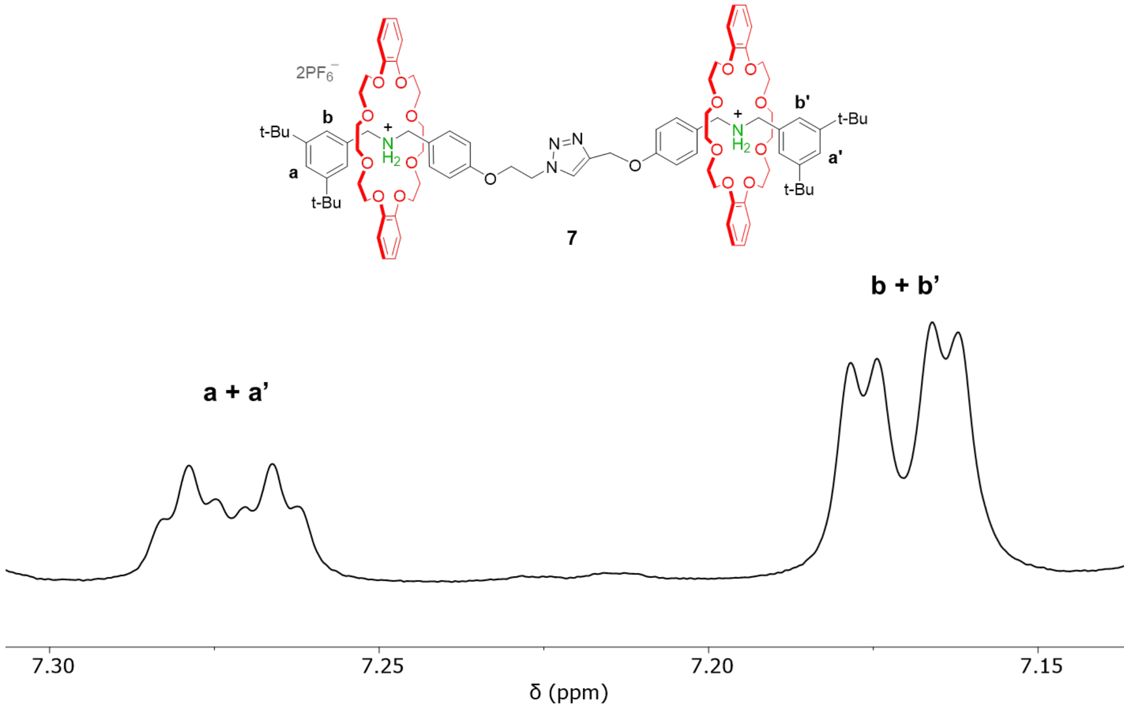

Figure S35. Detail of the ^{1}H NMR spectrum of **7** (Benzene-d_6:Methylene Chloride-d_2 70:30, 298 K, 500 MHz).

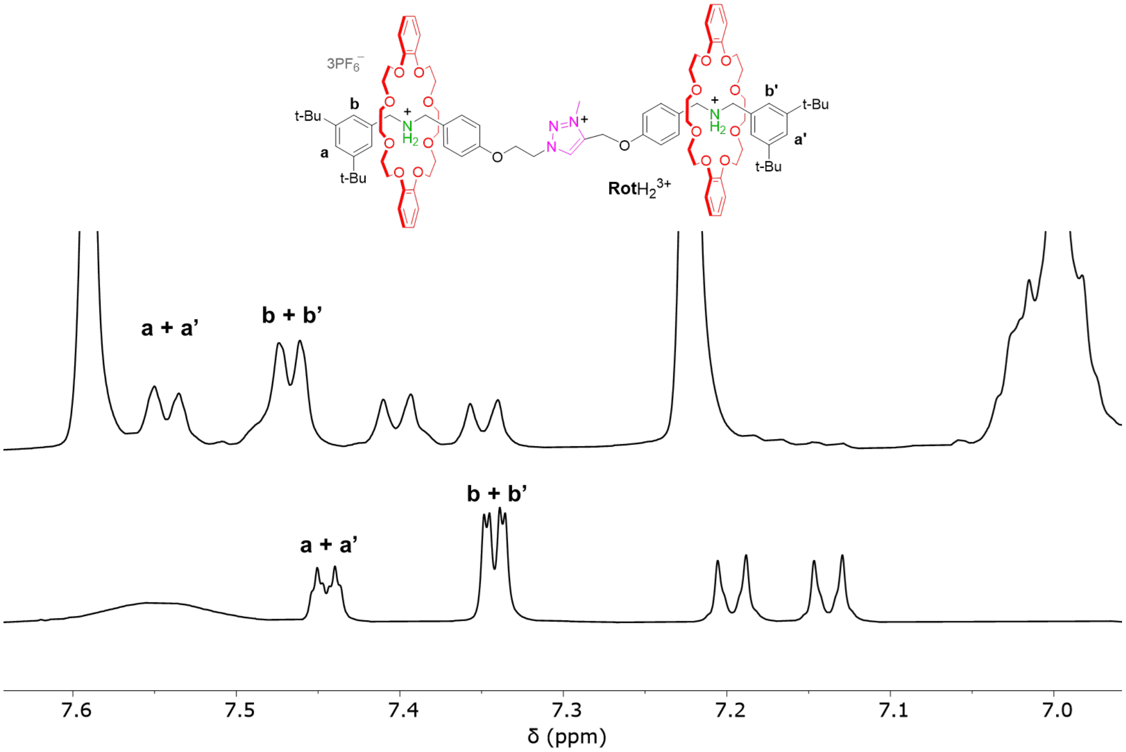

Figure S36. Detail of the ^{1}H NMR spectra of **Rot**H_2^{3+} in Pyridine-d_5 (top) and Acetonitrile-d_3 (bottom); 298 K, 500 MHz.

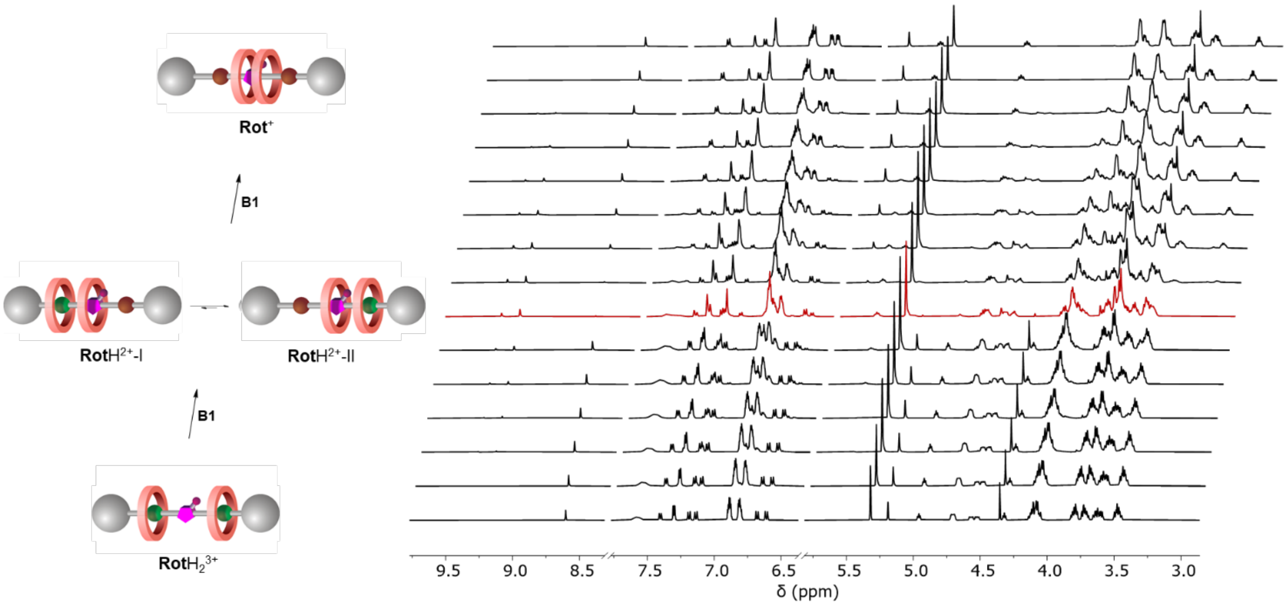

Figure S37. Deprotonation of **Rot**H_2^{3+} by addition of the heterogeneous base **B1** (Methylene Chloride-d_2, 298 K, 500 MHz); the red line indicates the spectrum obtained after addition of 1 equivalent of **B1**.

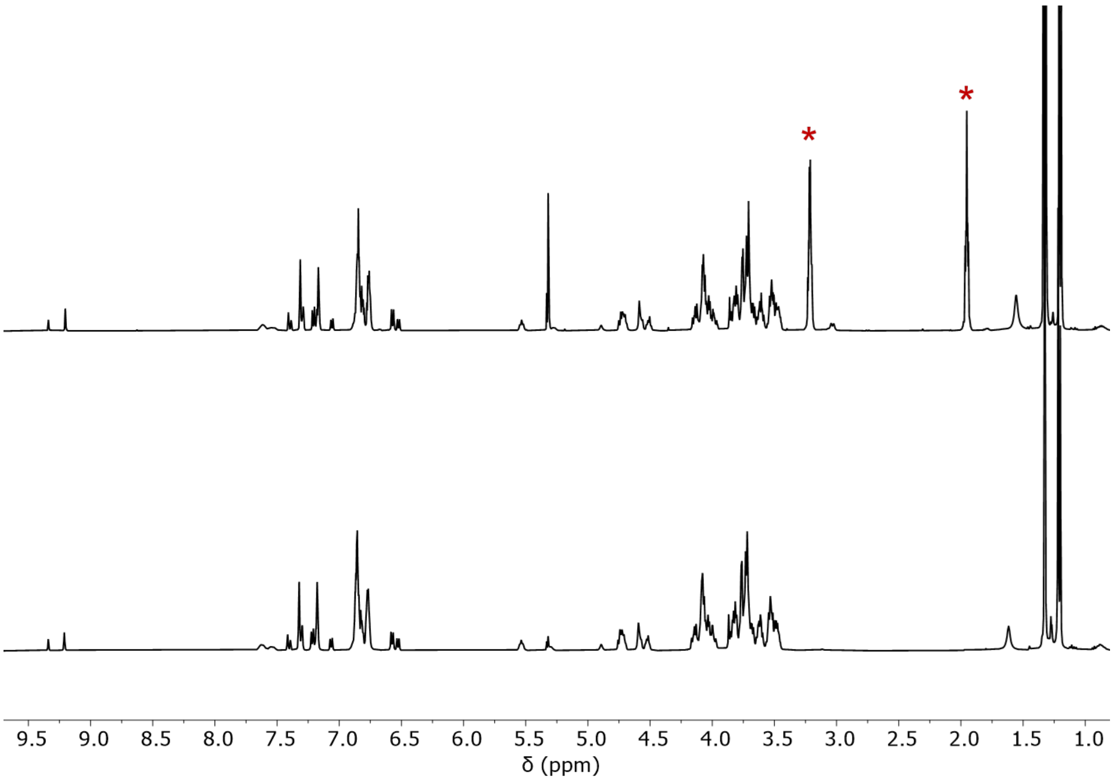

Figure S38. Comparison of the ^{1}H NMR spectra of **Rot**H_2^{3+} upon addition of 1 equivalent of the heterogeneous base **B1** (bottom) and 1 equivalent of the homogeneous base **B2** (top); the peaks marked with red asterisks are related to the conjugated acid [H**B2**][PF_6] (Methylene Chloride-d_2, 298 K, 500 MHz).

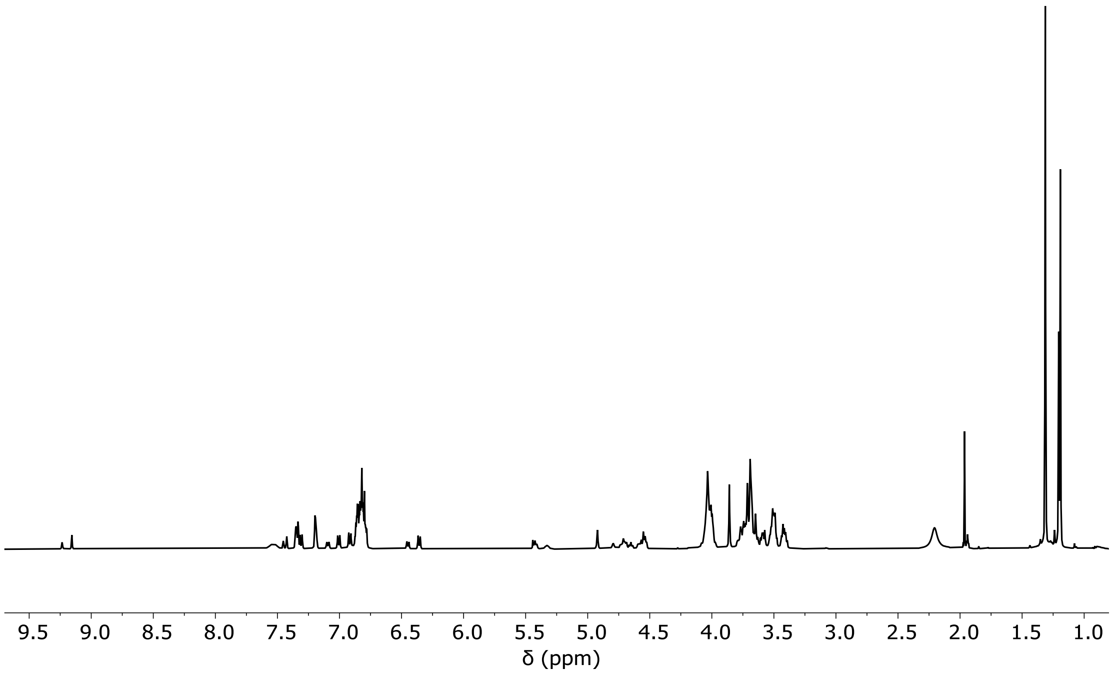

Figure S39. ^{1}H NMR spectrum of the mixture of compounds **Rot**H_2^+-I and **Rot**H_2^+-II (Acetonitrile-d_3, 298 K, 500 MHz).

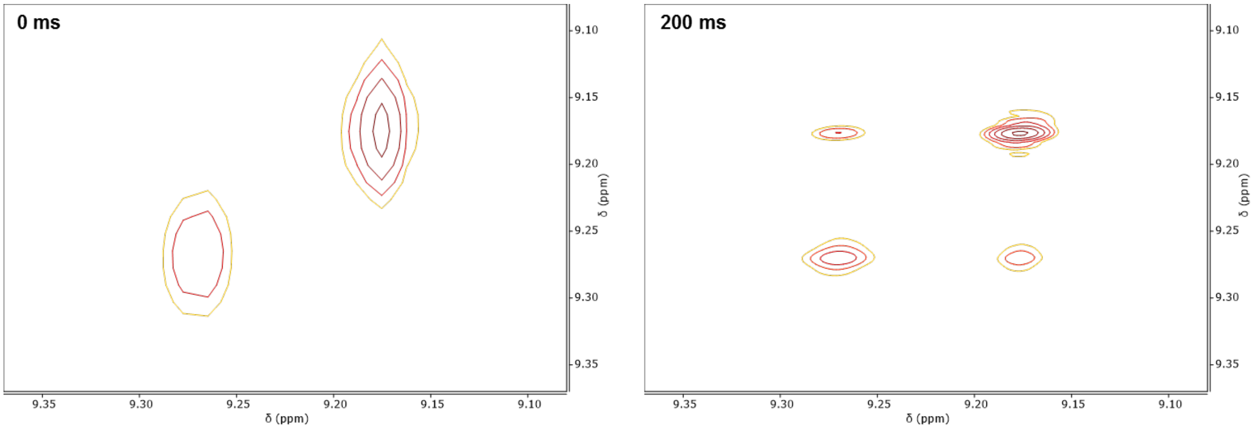

Figure S40. Portion of the ^{1}H-^{1}H EXSY spectrum of the mixture of **Rot**H_2^+-I and **Rot**H_2^+-II at mixing time = 0 ms (left) and at mixing time 200 ms (right); Acetonitrile-d_3, 298 K, 500 MHz.

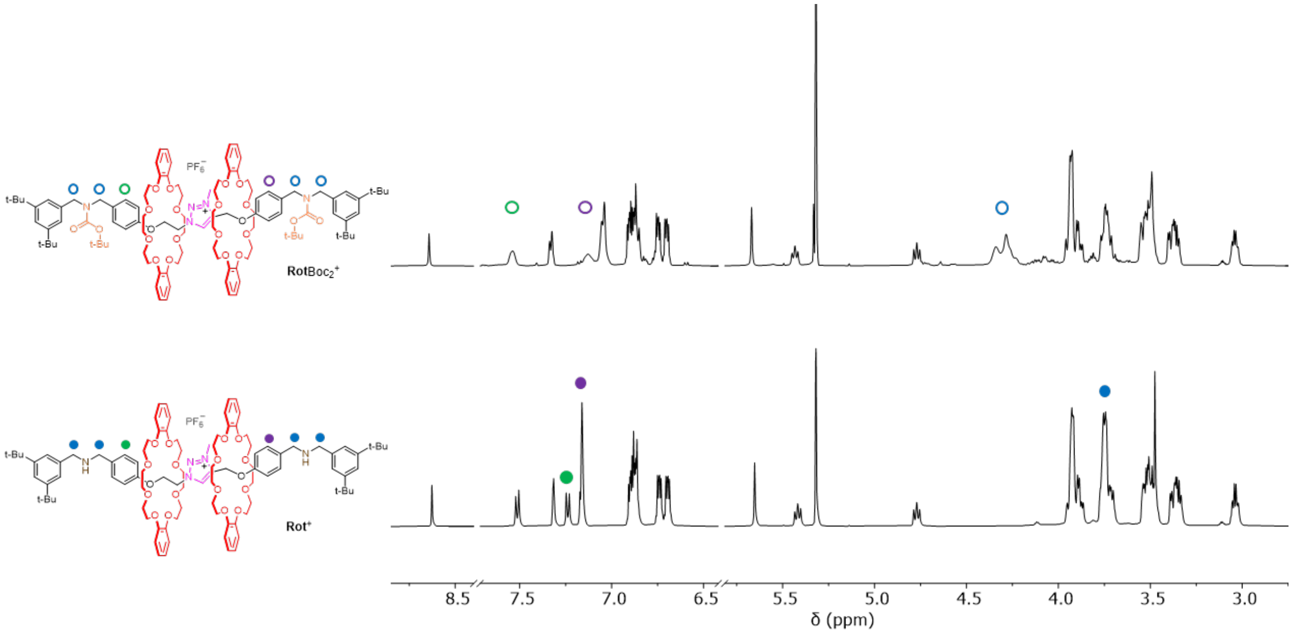

Figure S41. Comparison between the ^{1}H NMR spectra of **Rot**$^+$ and **Rot**Boc_2^+ (Methylene Chloride-d_2, 298 K, 500 MHz).

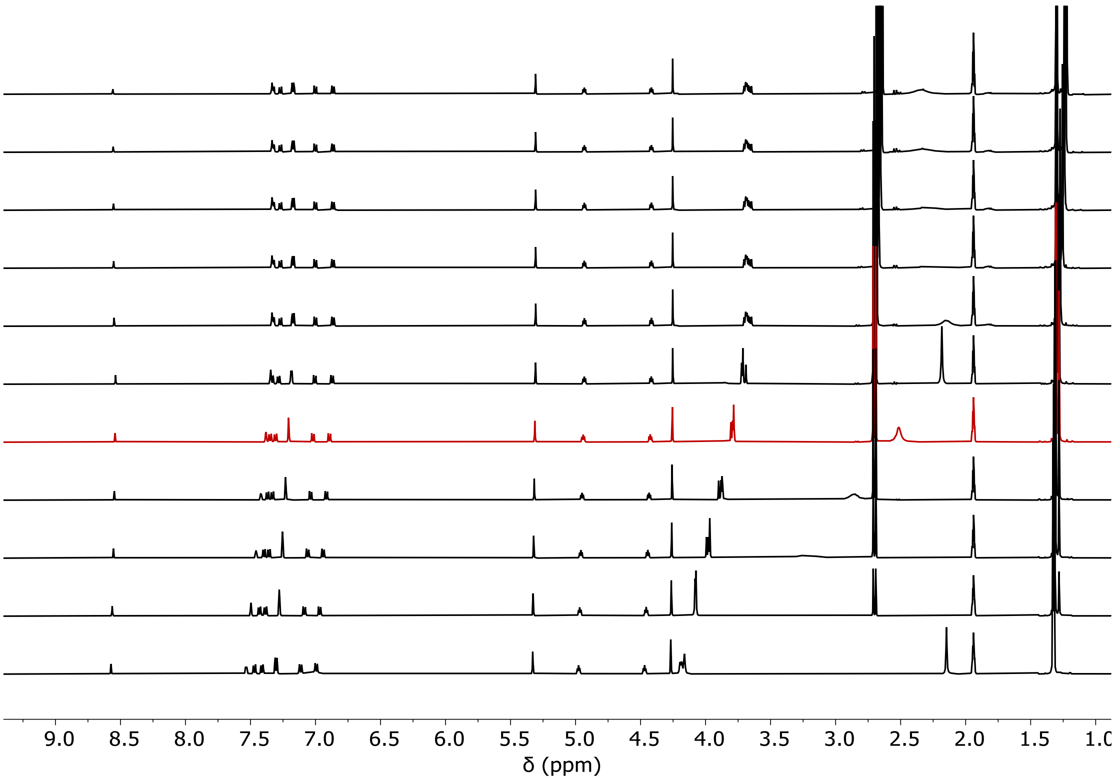

Figure S42. Deprotonation of **9** by addition of the homogeneous base **B2** (Acetonitrile-d_3, 298 K, 500 MHz); the red line indicates the spectrum obtained after addition of 1 equivalent of **B2**.

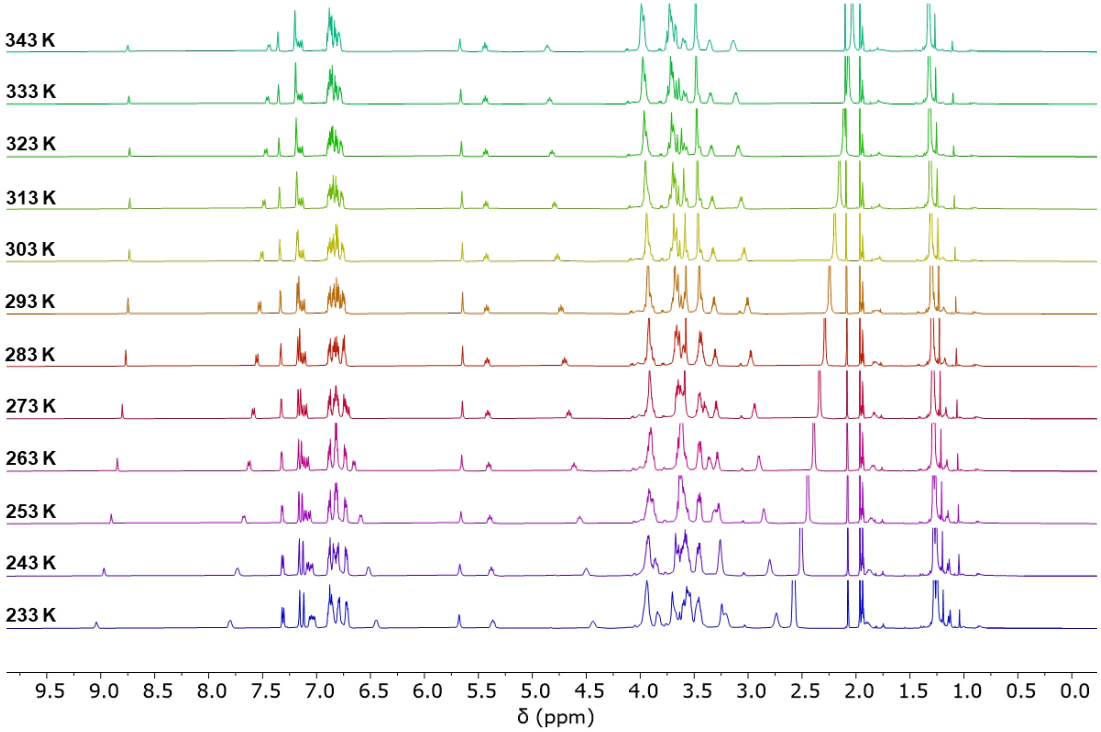

Figure S43. Variable-temperature ^{1}H NMR spectra of **Rot**$^+$ (Acetonitrile-d_3, 500 MHz).

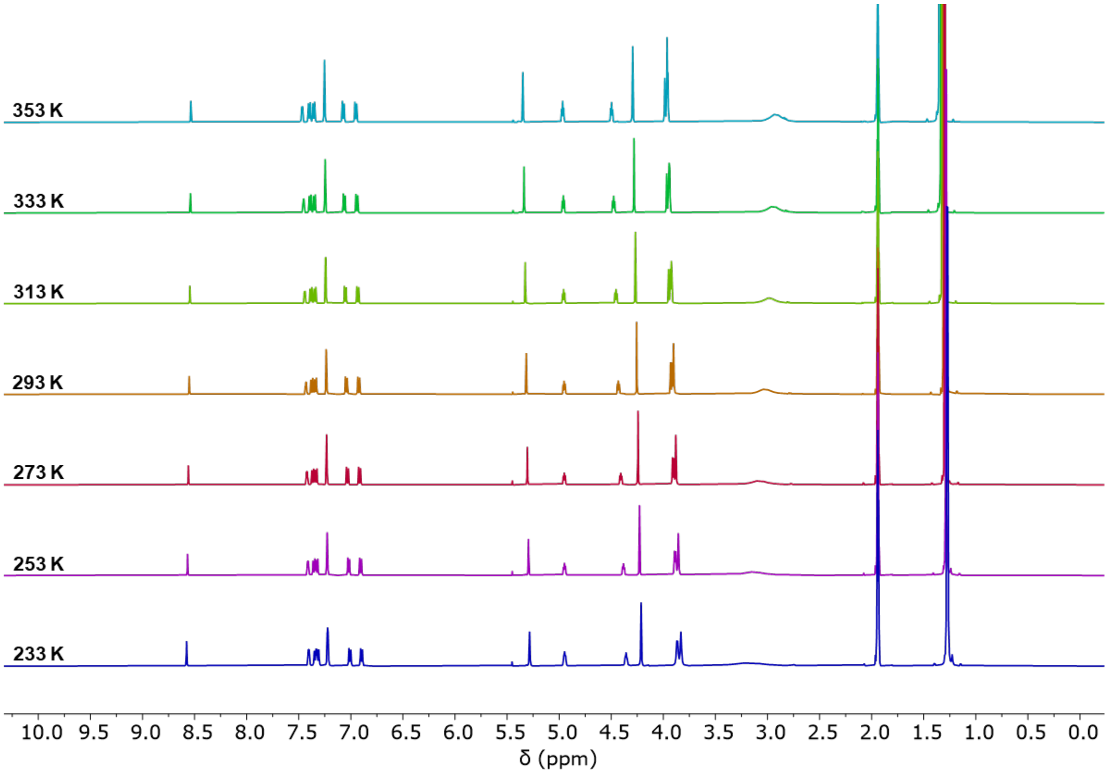

Figure S44. Variable-temperature ^{1}H NMR spectra of **10** (Acetonitrile-d_3, 500 MHz).

Spectrophotometric Data

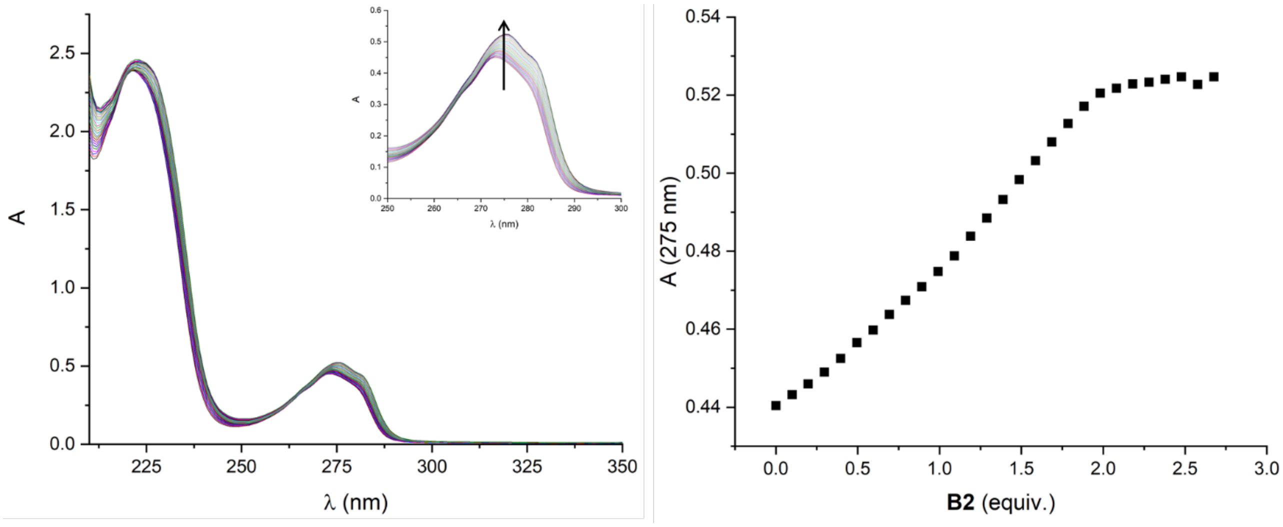

Figure S45. UV-Vis titration of **Rot**H_2^{3+} with **B2** (3.96×10^{-5} M, Acetonitrile).

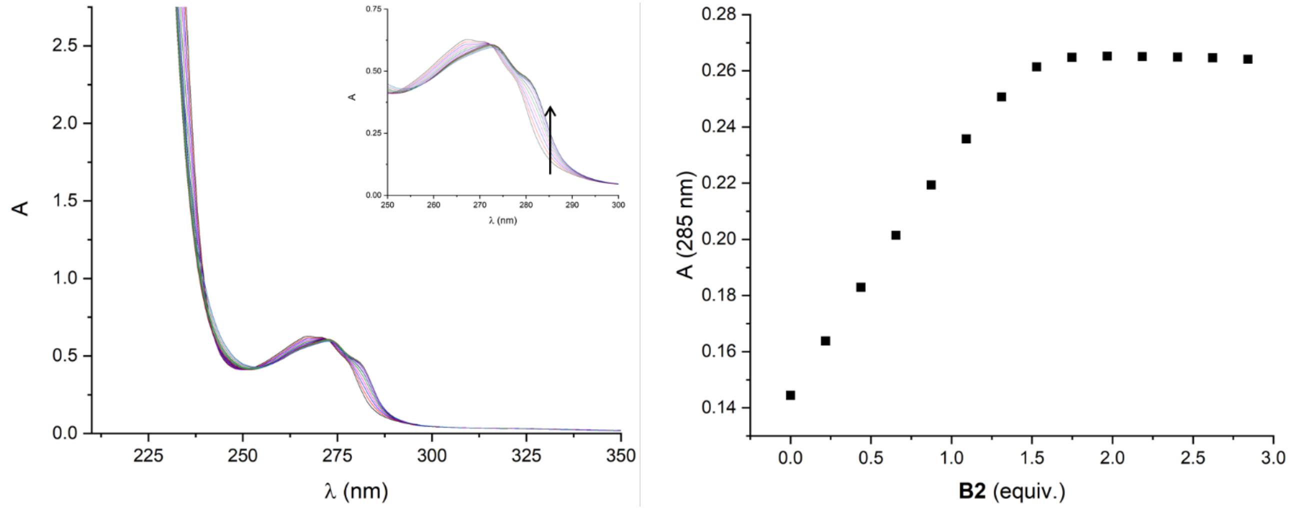

Figure S46. UV-Vis titration of **9** with **triethylamine** (3.13×10^{-5} M, Acetonitrile).

Thermodynamic analysis

The pK_a values of the individual rotaxane species were determined from NMR spectroscopic and UV-visible titration data according to the procedure shown in Figure S47. The only assumption made in the calculation is that **Rot**H^{2+}-I and **Rot**H^{2+}-II exhibit the same molar absorption coefficient at the observation wavelength. This is very reasonable because the two forms differ only for the position of the encircled ammonium unit with respect to the triazolium, and the DB24C8-dibenzylammonium interaction does not cause appreciable changes in the UV-visible absorption spectrum.[18]

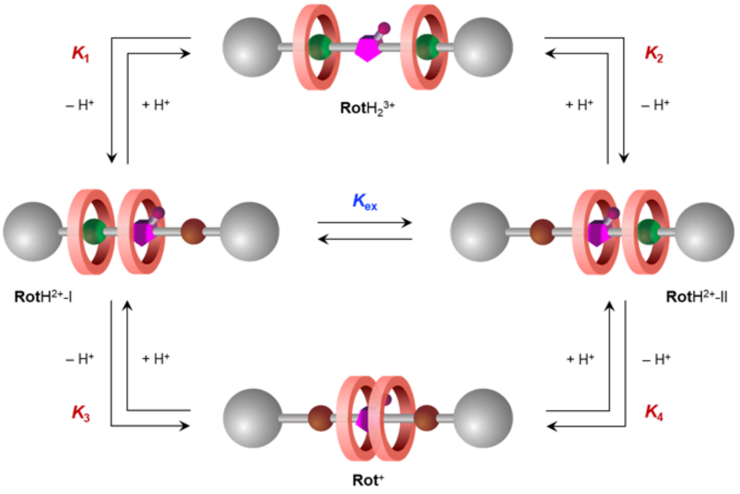

• Network at equilibrium
$$\begin{cases} K_1 \times K_{ex} = K_2 \\ K_3 = K_{ex} \times K_4 \\ K_1 \times K_3 = K_2 \times K_4 \end{cases}$$

• Very similar absorption spectra for **Rot**H^{2+}-I and **Rot**H^{2+}-II ($\varepsilon_I \sim \varepsilon_{II}$)

$$A_{obs} = \varepsilon_I[\mathbf{Rot}H^{2+}\text{-I}] + \varepsilon_{II}[\mathbf{Rot}H^{2+}\text{-II}]$$
$$A_{obs} \approx \varepsilon_{obs}([\mathbf{Rot}H^{2+}\text{-I}] + [\mathbf{Rot}H^{2+}\text{-II}])$$
$$A_{obs} \propto ([\mathbf{Rot}H^{2+}\text{-I}] + [\mathbf{Rot}H^{2+}\text{-II}])$$

$K_{ex} = 2.3$ From NMR spectroscopy

1) Solution of the top half network

• $K_1 = \dfrac{[\mathbf{Rot}H^{2+}\text{-I}][H^+]}{[\mathbf{Rot}H_2^{3+}]}$ • $K_2 = \dfrac{[\mathbf{Rot}H^{2+}\text{-II}][H^+]}{[\mathbf{Rot}H_2^{3+}]}$

• $K'_{obs} = \dfrac{([\mathbf{Rot}H^{2+}\text{-I}]+[\mathbf{Rot}H^{2+}\text{-II}])[H^+]}{[\mathbf{Rot}H_2^{3+}]} = K_1 + K_2$ p$K'_{obs} = 23.0$ From UV-vis titration fitting

$$\begin{cases} K_1 \times K_{ex} = K_2 \\ pK'_{obs} = 23.0 \end{cases} \rightarrow \begin{cases} K_2 = 2.3\, K_1 \\ K_1 + K_2 = 10^{-23.0} \end{cases} \rightarrow \begin{cases} K_2 = 10^{-23.2}\ \text{M} \\ K_1 = 10^{-23.5}\ \text{M} \end{cases}$$

2) Solution of the bottom half network

• $K_3 = \dfrac{[\mathbf{Rot}^+][H^+]}{[\mathbf{Rot}H^{2+}\text{-I}]}$ • $K_4 = \dfrac{[\mathbf{Rot}^+][H^+]}{[\mathbf{Rot}H^{2+}\text{-II}]}$

• $\dfrac{1}{K''_{obs}} = \dfrac{([\mathbf{Rot}H^{2+}\text{-I}]+[\mathbf{Rot}H^{2+}\text{-II}])}{[\mathbf{Rot}^+][H^+]} = \dfrac{1}{K_3} + \dfrac{1}{K_4}$ p$K''_{obs} = 24.7$ From UV-vis titration fitting

$$\begin{cases} K_3 = K_4 \times K_{ex} \\ pK''_{obs} = 24.7 \end{cases} \rightarrow \begin{cases} K_3 = 2.3\, K_4 \\ \dfrac{1}{K_3} + \dfrac{1}{K_4} = 10^{24.7} \end{cases} \rightarrow \begin{cases} K_3 = 10^{-24.2}\ \text{M} \\ K_4 = 10^{-24.5}\ \text{M} \end{cases}$$

Figure S47. Determination of the acid dissociation constants of **Rot**H$_2^{3+}$, **Rot**H^{2+}-I and **Rot**H^{2+}-II.

Computational results

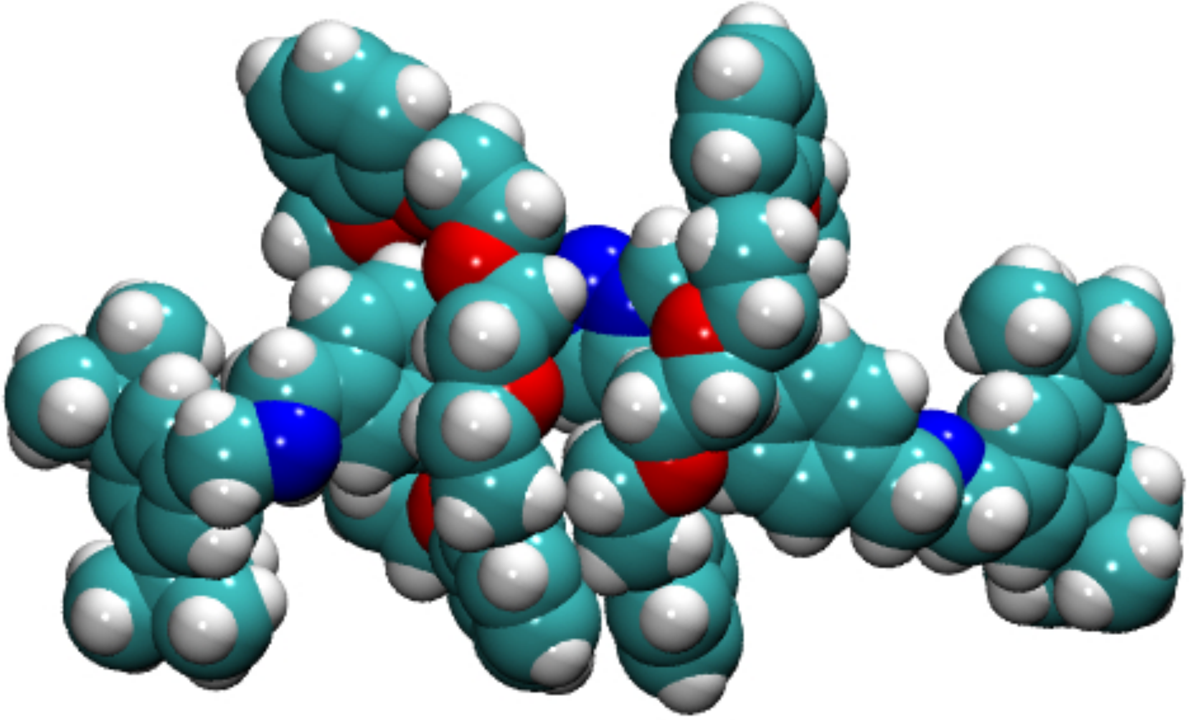

Figure S48. Graphical representation of the starting co-conformation of the **Rot**$^{+}$ monocation for the ab initio MTD runs. Color codes: C=cyan spheres; N=blue spheres; O=red spheres; H=white spheres. The part of the axle containing the ethyl bridge is on the left side of the [3]rotaxane in this representation, while the part of the axle containing the methyl bridge is on the right side. The positively charged Tz station is in the middle of the axle, between the two DB24C8 macrocycles.

The starting co-conformation was identical for the MTD runs, it features the two rings at opposite sides of the Tz station (**Figure S48**). Such co-conformation (including both positions and velocities) represents the zero of our MTD sampling. The considered CV implied the displacement of the oxygen atoms of one ring with respect to the nitrogen atoms of the Tz station (**Figure S48**). As there are two rings, we performed two different MTD runs picking alternatively one of the two rings to transit along the axle. As the initial co-conformation for the MTD samplings was identical (and taken as the zero of the processes) we collapsed the free energy profiles of the different runs in one single profile (Figure 6 in the main text).

Overall, we detected three deep minima in the free energy profile. In order to calculate thermally averaged proton chemical shifts, we sampled some tens of co-conformations extracted from the regions corresponding to the three free energy minima and used such co-conformations for the calculations of the proton NMR data. The chemical shifts computed from the three sampled regions were then averaged.

Optimized geometry of RotH_2^{3+}, **Rot**H^{2+}-I **and Rot**H^{2+}-II

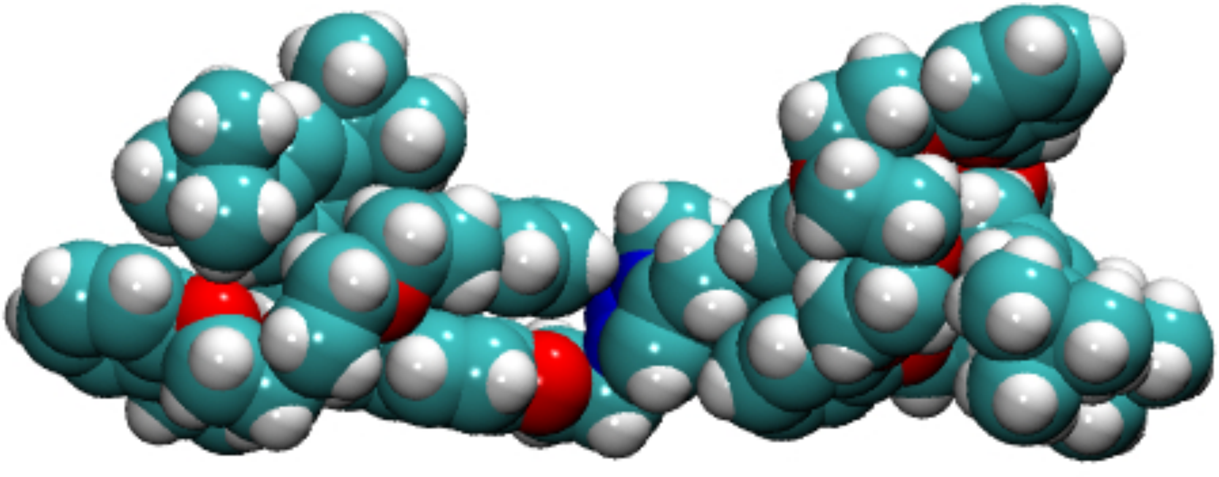

Figure S49. Optimized geometry of **Rot**H_2^{3+}. Color codes as in Figure S48.

In the optimized geometry of parent compound **Rot**H_2^{3+}(Figure S49), each of the two DB24C8 macrocycles are located on a positively charged Am station. Besides the favorable electrostatic interactions of the oxygen atoms of the rings with the positively charged dibenzylammonium moieties, the structure is stabilized by π-π interactions between the phenyl groups of both rings and the aryl stoppers of the axle. This feature is clearly evidenced by the face-to-face arrangement of the aromatic moieties of the rings with the close aryl groups of the axle. Also, strong hydrogen bonds are formed between the $-NH_2^+$ moieties of Am stations and the oxygen atoms of the rings. The hydrogen bond distances are 1.857 and 1.882 Å for the ring on the ethyl side and methyl side, respectively.

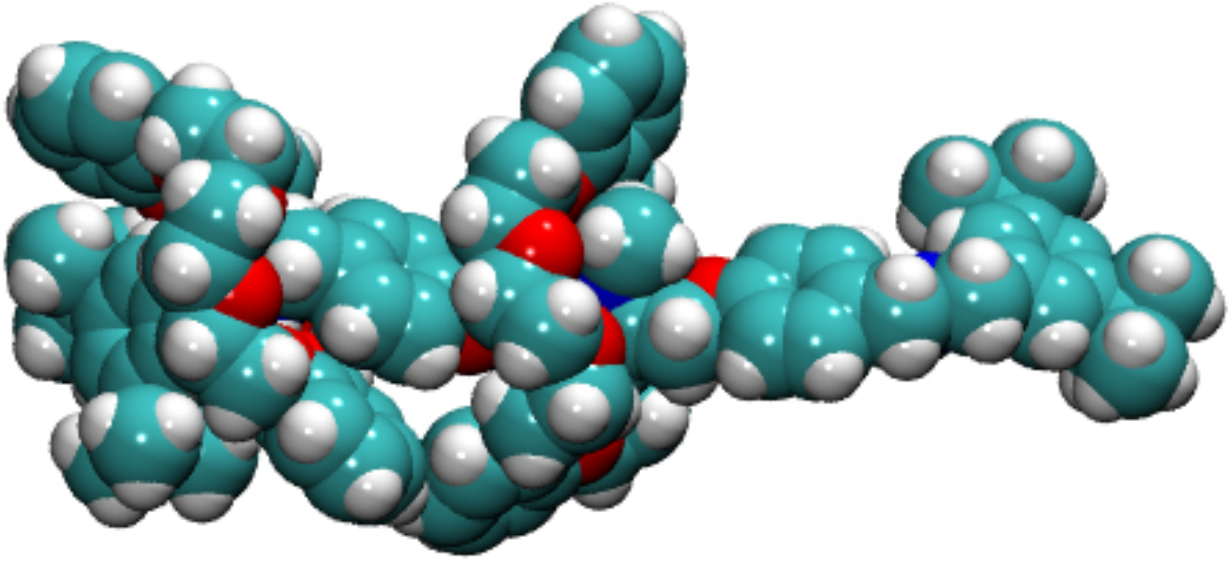

Figure S50. Optimized geometry of **Rot**H^{2+}-I (B2). Color codes as in Figure S48**.**

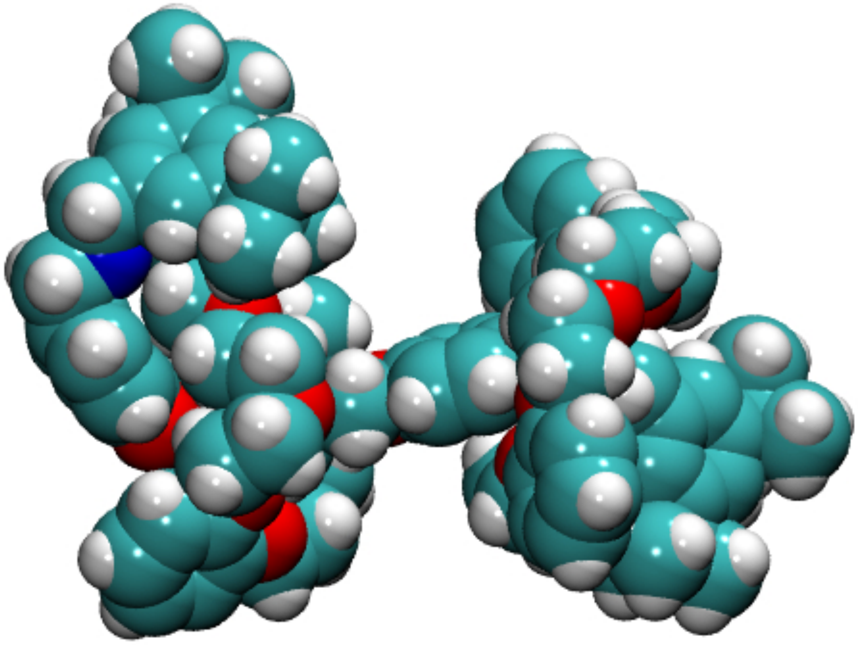

Figure S51. Optimized geometry of **Rot**H^{2+}-II (B1). Color codes as in Figure S48.

The optimized geometries of the dications **Rot**H^{2+}-I and **Rot**H^{2+}-II are reported in Figure S50 and Figure S51, respectively. The structures of these dications are markedly different with respect to the trication, because of the absence of face-to-face π-π interactions of the rings with the aryl moieties of the axle. Also, the two dicationic structures are also significantly different between each other.

Specifically, in **Rot**H^{2+}-I (deprotonation of the Am on the methylene side), the ring which was previously on the methylene side, upon deprotonation has translated along the axle up to the Tz station, and has formed a new hydrogen bond with the Tz proton (distance H(Tz)-O_{ring} = 2.118 Å). The ring on the ethylene side remains bound to its Am station - which is still protonated – via a hydrogen bond interaction (distance H(NH_2^+)-O_{ring} = 1.910 Å). No substantial rearrangement of the methyl side of the axle are noticed, which remains approximately in a linear conformation.

Conversely, in **Rot**H^{2+}-II (deprotonation of the Am on the ethylene side), the ring which was previously on the ethylene side, upon deprotonation of its Am station has migrated to the Tz station and has formed a new hydrogen bond with the Tz proton (distance H(Tz)-O_{ring} = 2.050 Å). The ring on the methylene side remains bound to its Am station - which is still protonated - via a hydrogen bond interaction (distance H(NH_2^+)-O_{ring} = 1.894 Å). Interestingly, this translational movement has been accompanied by a noticeable rearrangement of the ethylene side of the axle, suggesting a greater conformational flexibility with respect to the methylene side of the axle.

The energy difference between the optimized geometries **Rot**H^{2+}-I and **Rot**H^{2+}-II amounted to 1.4 kcal mol^{-1} in favour of the **Rot**H^{2+}-II structure. The slightly greater stability of **Rot**H^{2+}-II may be ascribed to the edge-to-face π-π interaction of the ring on the methylene side of the axle, evidenced in Figure S51, as well as to the stronger hydrogen-bond interactions between the rings and the axle components.

Simulated NMR spectra

Figure S52 reports the computed ^{1}H-NMR spectra of the parent compound **Rot**H_2^{3+} and of dications **Rot**H^{2+}-I and **Rot**H^{2+}-II, while Figure S53 reports the the computed ^{1}H-NMR spectra for the three free energy minima obtained from the free energy profile, labelled as A, B, C (see also main text). Graphical representations of three co-conformations sampled from the above-mentioned energy minima A, B, C are reported in Figures S54, S55 and S56, respectively.

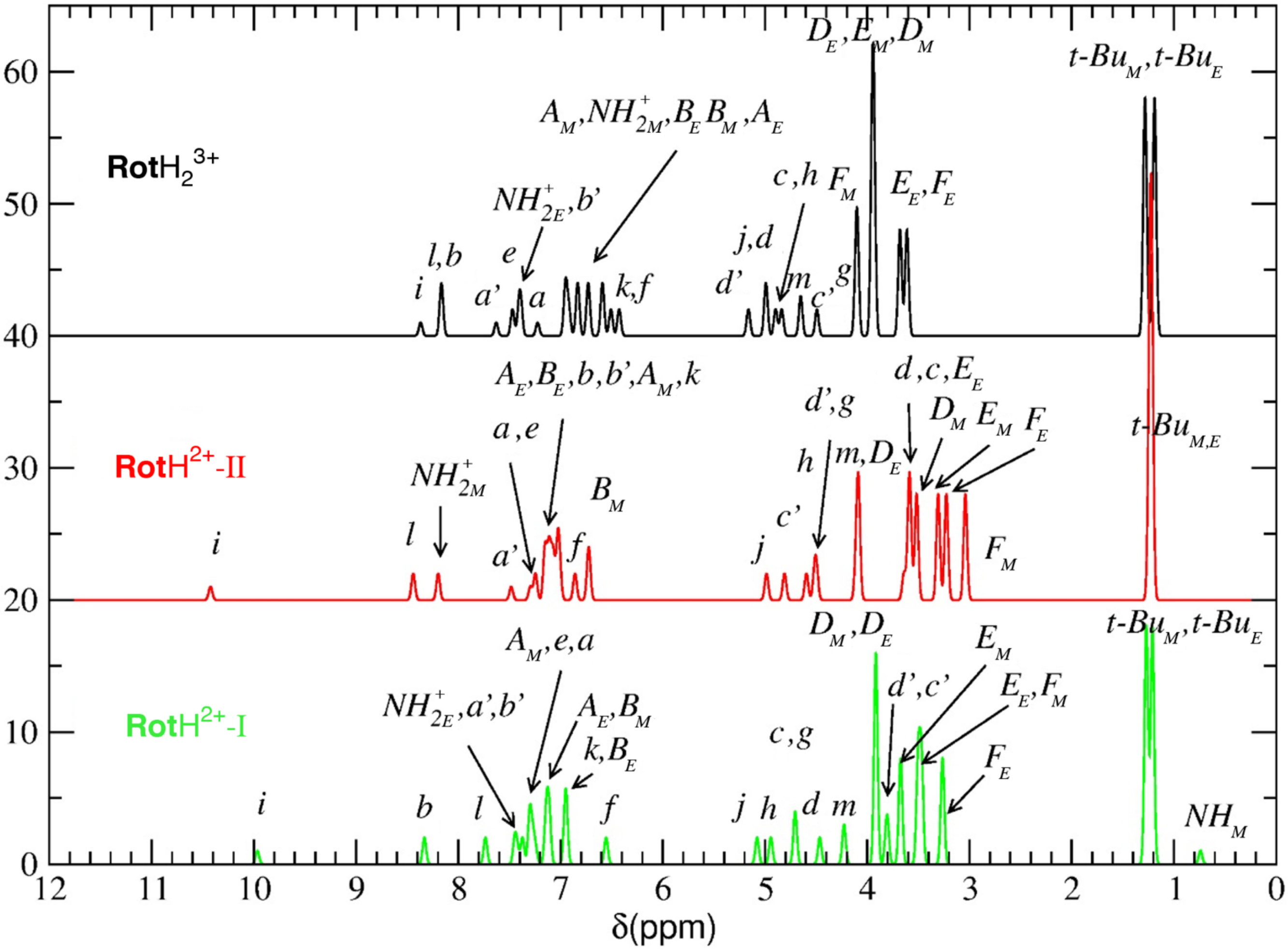

Figure S52. Simulated ^{1}H-NMR spectra of the optimized geometry of **Rot**H_2^{3+} (top panel, black line); **Rot**H^{2+}-II (middle panel, red line); **Rot**H^{2+}-I (bottom panel, green line). Labels are as shown in the structures reported below. Lower case and upper case letters refer respectively to protons of the axle and of the two rings. The subscripts *M*, *E*, refer to protons on the methylene (*M*) or ethylene (*E*) side of the axle, respectively. Proton labelled as *i* corresponds to proton H_{Tz} in the main text.

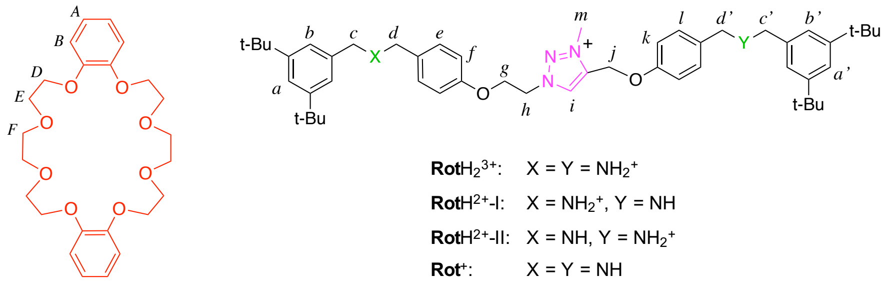

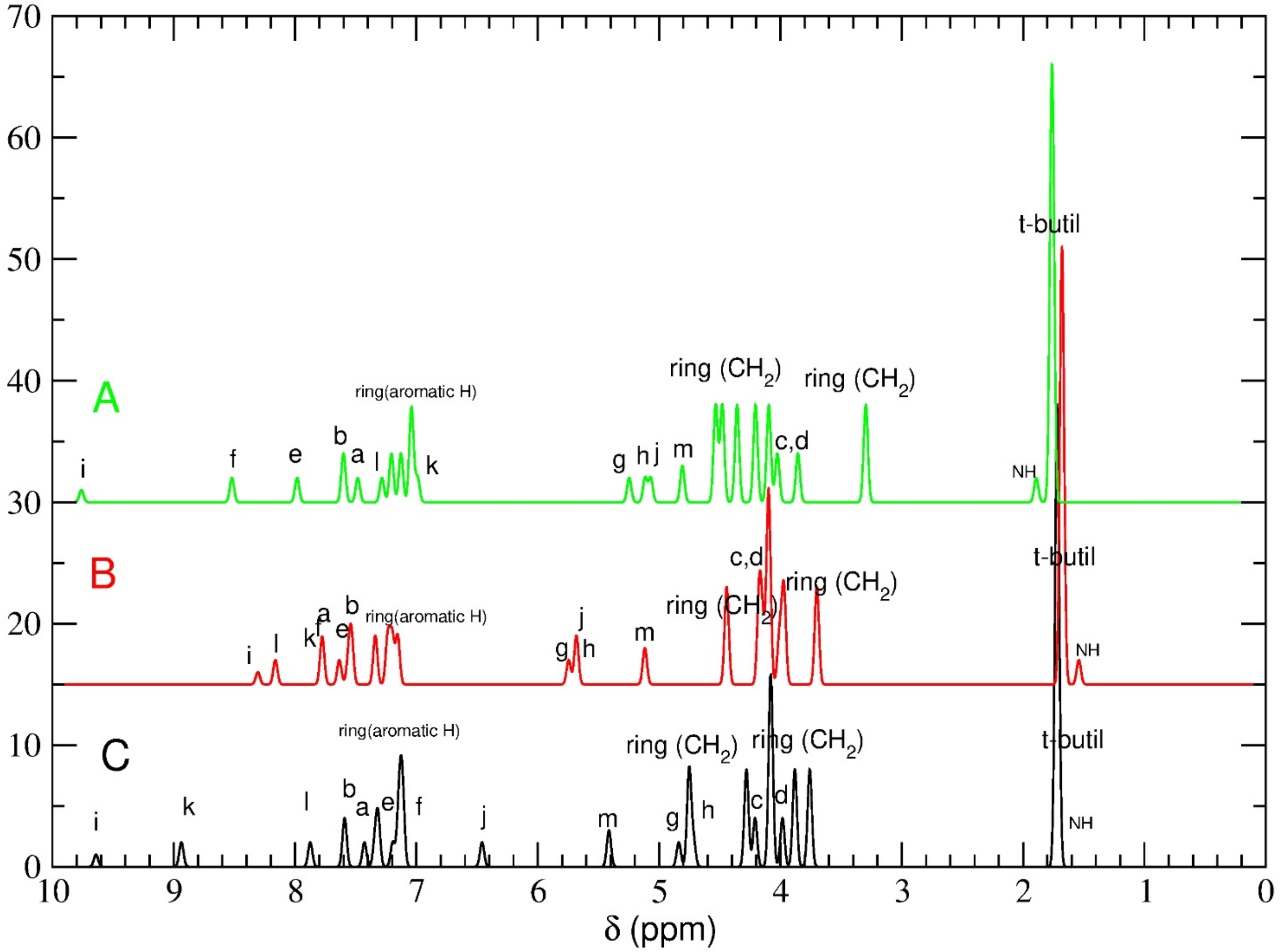

Figure S53. Simulated ^{1}H-NMR spectra for the free energy minima **A** (top panel, green line); **B** (middle panel, red line); **C** (bottom panel, black line). Proton labelled as *i* corresponds to proton H_{Tz} in the main text.

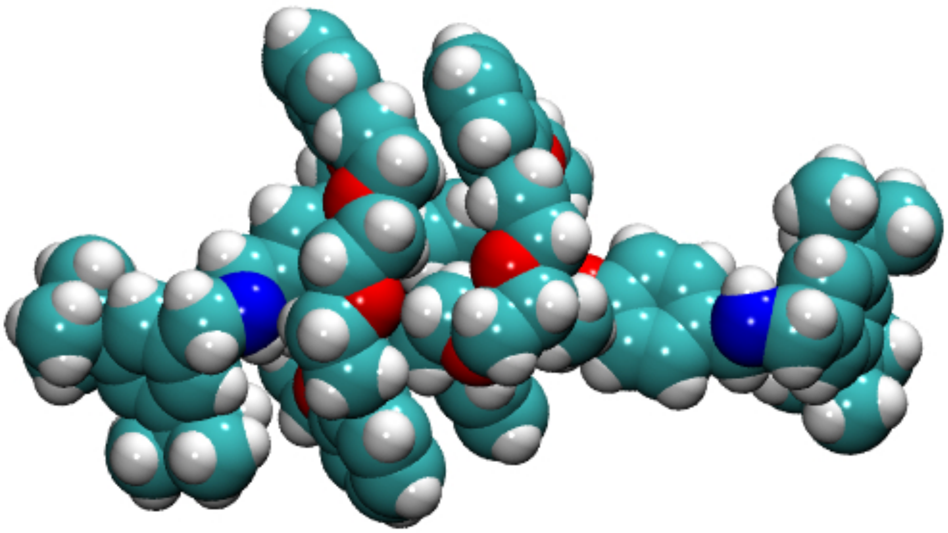

Figure S54. Co-conformation of **Rot**$^+$ sampled from free energy minimum A. Colors as in Figure S48.

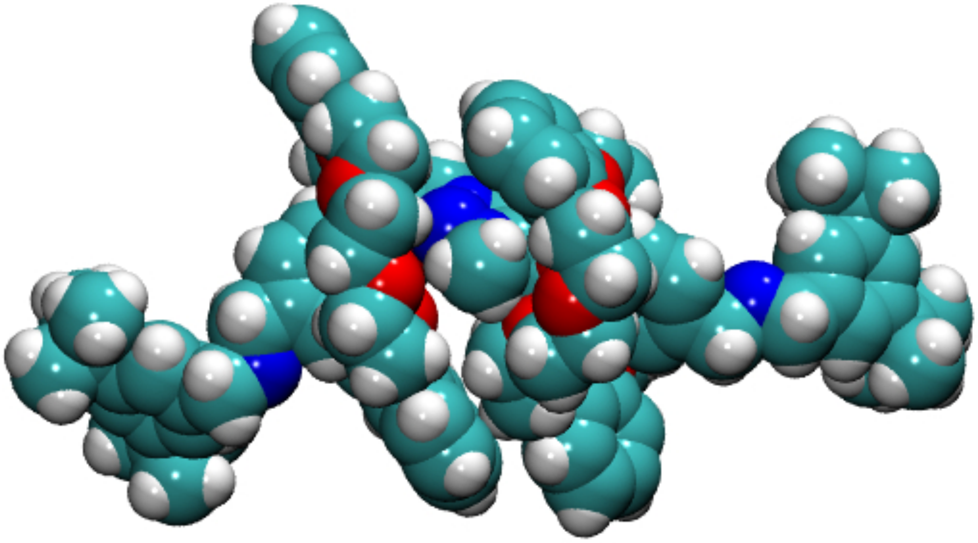

Figure S55. Co-conformation of **Rot**$^{+}$ sampled from free energy minimum B. Colors as in Figure S48.

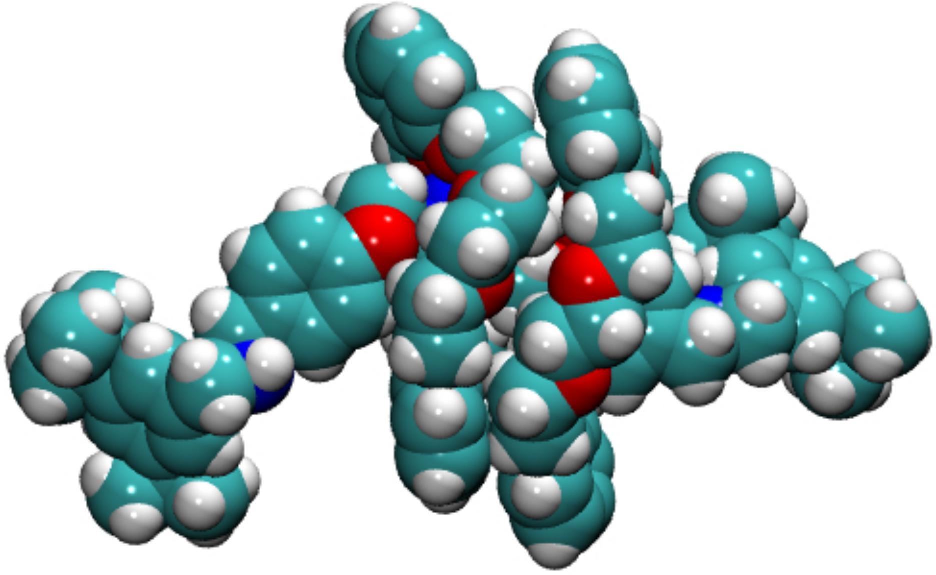

Figure S56. Co-conformation of **Rot**$^{+}$ sampled from free energy minimum C. Colors as in Figure S48.

REFERENCES

(1) Dunning, T. H. Gaussian Basis Sets for Use in Correlated Molecular Calculations. I. The Atoms Boron through Neon and Hydrogen. *J. Chem. Phys.* **1989**, *90*, 1007-1023. https://doi.org/10.1063/1.456153.

(2) Chai, J.-D.; Head-Gordon, M. Long-Range Corrected Hybrid Density Functionals with Damped Atom–Atom Dispersion Corrections. *Phys. Chem. Chem. Phys.* **2008**, *10*, 6615. https://doi.org/10.1039/b810189b.

(3) Gauss, J. Effects of Electron Correlation in the Calculation of Nuclear Magnetic Resonance Chemical Shifts. *J. Chem. Phys.* **1993**, *99*, 3629-3643. https://doi.org/10.1063/1.466161.

(4) Woon, D. E.; Dunning, T. H. Gaussian Basis Sets for Use in Correlated Molecular Calculations. III. The Atoms Aluminum through Argon. *J. Chem. Phys.* **1993**, *98*, 1358-1371. https://doi.org/10.1063/1.464303.

(5) Jain, R.; Bally, T.; Rablen, P. R. Calculating Accurate Proton Chemical Shifts of Organic Molecules with Density Functional Methods and Modest Basis Sets. *J. Org. Chem.* **2009**, *74*, 4017-4023. https://doi.org/10.1021/jo900482q.

(6) Frisch, M. J.; Trucks, G. W.; Schlegel, H. B.; Scuseria, G. E.; Robb, M. A.; Cheeseman, J. R.; Scalmani, G.; Barone, V.; Mennucci, B.; Petersson, G. A.; Nakatsuji, H.; Caricato, M.; Li, X.; Hratchian, H. P.; Izmaylov, A. F.; Bloino, J.; Zheng, G.; Sonnenb, D. J. Gaussian 09, Revision E.01,. Gaussian, Inc.: Wallingford CT 2009.

(7) Tomasi, J.; Mennucci, B.; Cammi, R. Quantum Mechanical Continuum Solvation Models. *Chem. Rev.* **2005**, *105*, 2999-3094.

(8) Car, R.; Parrinello, M. Unified Approach for Molecular Dynamics and Density-Functional Theory. *Phys. Rev. Lett.* **1985**, *55*, 2471-2474. https://doi.org/10.1103/PhysRevLett.55.2471.

(9) Laio, A.; Parrinello, M. Escaping Free-Energy Minima. *Proc. Natl. Acad. Sci. U. S. A.* **2002**, *99*, 12562-12566. https://doi.org/10.1073/pnas.202427399.

(10) Iannuzzi, M.; Laio, A.; Parrinello, M. Efficient Exploration of Reactive Potential Energy Surfaces Using Car-Parrinello Molecular Dynamics. *Phys. Rev. Lett.* **2003**, *90*, 4. https://doi.org/10.1103/PhysRevLett.90.238302.

(11) Perdew, J. P.; Burke, K.; Ernzerhof, M. Generalized Gradient Approximation Made Simple. *Phys. Rev. Lett.* **1996**, *77*, 3865-3868. https://doi.org/10.1103/PhysRevLett.77.3865.

(12) Grimme, S. Semiempirical GGA-Type Density Functional Constructed with a Long-Range Dispersion Correction. *J. Comput. Chem.* **2006**, *27*, 1787-1799. https://doi.org/10.1002/jcc.20495.

(13) Vanderbilt, D. Soft Self-Consistent Pseudopotentials in a Generalized Eigenvalue Formalism. *Phys. Rev. B* **1990**, *41*, 7892-7895. https://doi.org/10.1103/PhysRevB.41.7892.

(14) Nosé, S. A Unified Formulation of the Constant Temperature Molecular Dynamics Methods. *J. Chem. Phys.* **1984**, *81*, 511-519. https://doi.org/10.1063/1.447334.

(15) Hoover, W. G. Canonical Dynamics: Equilibrium Phase-Space Distributions. *Phys. Rev. A* **1985**, *31*, 1695-1697. https://doi.org/10.1103/PhysRevA.31.1695.

(16) Marx, D.; Hutter, J. *Ab Initio Molecular Dynamics*; Cambridge University Press: Cambridge, 2009. https://doi.org/10.1017/CBO9780511609633.

(17) IBM Corp. 1990–2017; MPI für Festkörperforschung Stuttgart 1997–2001. CPMD: Car Parrinello Molecular Dynamics. 2017.

(18) Ashton, P. R.; Ballardini, R.; Balzani, V.; Gomez-Lopez, M.; Lawrence, S. E.; Martinez-Diaz, M. V.; Montalti, M.; Piersanti, A.; Prodi, L. Stoddart, J. F.; Williams, D. J. Hydrogen-Bonded Complexes of Aromatic Crown Ethers with (9-Anthracenyl)methylammonium Derivatives. Supramolecular Photochemistry and Photophysics. pH-Controllable Supramolecular Switching. *J Am. Chem. Soc.* **1997**, *119*, 10641-10651.
